# Molecular mapping of QTLs for yield related traits in recombinant inbred line (RIL) population derived from the popular rice hybrid KRH-2 and their validation through SNP genotyping

**DOI:** 10.1038/s41598-020-70637-3

**Published:** 2020-08-13

**Authors:** Swapnil Ravindra Kulkarni, S. M. Balachandran, K. Ulaganathan, Divya Balakrishnan, M. Praveen, A. S. Hari Prasad, R. A. Fiyaz, P. Senguttuvel, Pragya Sinha, Ravindra R. Kale, G. Rekha, M. B. V. N. Kousik, G. Harika, M. Anila, E. Punniakoti, T. Dilip, S. K. Hajira, K. Pranathi, M. Ayyappa Das, Mastanbee Shaik, K. Chaitra, P. Koteswara Rao, Sunil S. Gangurde, Manish K. Pandey, R. M. Sundaram

**Affiliations:** 1 Crop Improvement Section, ICAR-Indian Institute of Rice Research (ICAR-IIRR), Rajendranagar, Hyderabad, 500030 India; 2 Centre for Plant Molecular Biology (CPMB), Osmania University, Hyderabad, India; 3 International Crops Research Institute for the Semi-Arid Tropics (ICRISAT), Hyderabad, India

**Keywords:** Biotechnology, Genetics, Plant sciences

## Abstract

The study was undertaken to identify the quantitative trait loci (QTLs) governing yield and its related traits using a recombinant inbred line (RIL) population derived from the popular rice hybrid, KRH-2 (IR58025A/KMR3R). A genetic map spanning 294.2 cM was constructed with 126 simple sequence repeats (SSR) loci uniformly distributed across the rice genome. QTL analysis using phenotyping and genotyping information identified a total of 22 QTLs. Of these, five major effect QTLs were identified for the following traits: total grain yield/plant (*qYLD3-1*), panicle weight (*qPW3-1),* plant height (*qPH12-1),* flag leaf width (*qFLW4-1)* and panicle length (*qPL3-1*), explaining 20.23–22.76% of the phenotypic variance with LOD scores range of 6.5–10.59. Few genomic regions controlling several traits (QTL hotspot) were identified on chromosome 3 for total grain yield/plant (*qYLD3-1)* and panicle length (*qPL3-1*). Significant epistatic interactions were also observed for total grain yield per plant (YLD) and panicle length (PL). While most of these QTLs were observed to be co-localized with the previously reported QTL regions, a novel, major QTL associated with panicle length (*qPL3-1*) was also identified. SNP genotyping of selected high and low yielding RILs and their QTL mapping with 1,082 SNPs validated most of the QTLs identified through SSR genotyping. This facilitated the identification of novel major effect QTLs with much better resolution and precision. In-silico analysis of novel QTLs revealed the biological functions of the putative candidate gene (s) associated with selected traits. Most of the high-yielding RILs possessing the major yield related QTLs were identified to be complete restorers, indicating their possible utilization in development of superior rice hybrids.

## Introduction

Rice, a predominant crop caters to the calorific needs of half of the world’s population^1,2^. By 2025, in order to meet the growing demands of global rice consumption, the rice yield must reach 800 to 900 million tonnes^3^ which are significantly higher than the present level of rice production of 600 million tonnes^4^. Achieving the projected increase in the rice yield amidst diminishing natural and agricultural resources remains a formidable challenge. Though future challenges cannot be overseen, 50% of the global rice production has been achieved by the adoption of novel rice cultivars^5^. Further, the yield of modern rice cultivars has reached a plateau since several decades due to the narrow genetic diversity among the breeding lines^6^. Significant narrow genetic diversity of only 10%-20% in these modern rice cultivars is a result of modern breeding and domestication^5^. One of the plausible options to break the yield plateau in modern rice cultivars in a short duration is through relying on hybrid rice technology and rice hybrids are known to have a considerable yield advantage over the inbred lines^7^. Nevertheless, lesser scale of adoption of this technology by Indian farmers is due to higher seed costs and lower levels of heterosis than the projected yield advantage and poor cooking quality^8^. For more than two decades, improvement in the yield of modern cultivars has been achieved with marker-assisted selection (MAS) strategy^9,10^. Molecular approaches such as quantitative trait loci mapping can be relied in order to increase the yield and its stability in rice^11–14^. QTL mapping, a method that identifies the number, position and effect of loci on the expression of specific traits^15^ has been extensively used for the identification of novel QTLs in rice by various research groups^16^. This problem was addressed using a combination of conventional and molecular breeding approaches such as the marker assisted selection (MAS) which led to the identification of yield enhancing gene(s) or QTLs that produced new rice varieties with higher and consistent yields^16,18–20^. The rice yield is generally dependent on some of the important agro-morphological parameters namely number of productive tillers, number of filled grains per panicles and number of spikelets present per panicle^21^. The lesser yield levels in rice cultivars are due to the smaller panicles in rice resulted from the loss of beneficial alleles producing unproductive tillers^17^. One of the easiest ways to enhance the grain number is through the introduction of genes that control the high grain number genes or quantitative trait loci (QTL) such as *Gn1a* and *APO1* into elite rice cultivars^22^. Exploitation of Hap 3 haplotypes^23^ and GS3^24^ for enhancing yield in various rice species through MAS is well documented. Apart from yield, the MAS strategy has also been used in understanding many other crucial and complex agricultural traits such as drought^25^, cold tolerance^26^. Moreover, the MAS strategy has also been used to address biotic stresses^27^ and introgression of five genes that confer resistance to biotic stress into the *japonica* cultivar were well characterized. The characterization of such novel QTLs is not only crucial in understanding the genetic basis of traits' expressions but are also important from breeding perspectives^28^. The QTLs that influence the quantitative traits are influenced by environment which tends to show variation due to genotype × environment (G × E) interaction^29^. Identification of significant G × E interactions is carried out by comparing the QTL effects across various environments. Detection of QTLs in one environment and their absence in another is an indication of G × E effect's predominance whereas the consistent expression of QTLs across various environments is an indicator of their stability. Such consistent QTLs find an important application in marker assisted selection in plant breeding programs^30,31^.

Economically important traits such as yield are known to demonstrate complex genetic architecture which are polygenic in nature. Such complex interactions are usually affected by genotype × environment (G × E) interaction. Genetic architecture of rice grain yields are often influenced by two crucial traits namely the tiller number (TN) and the morphology of the panicle^32^. Tillering and panicle development in rice share a botanical commonality as manifestation of these traits is associated with apical growth and branching^33^. Therefore, the definite plant architecture and panicle size is largely due to the balance between apical dominance and branching that predominates the genetic architecture of grain yield trait. Pertaining to the tillering number (TN) trait^34^, described the two step developmental process of shoot branching that regulates the number of tillers. The first step is the axillary meristem formation and the second being axillary buds' growth. Three important monoculm genes namely *MOC1*, *MOC2* and *MOC3* controlling the axillary bud formation and its outgrowth have been identified in rice^35^. Molecular characterization of these three genes *MOC1* (*Os06g40780*), *MOC2* (*Os01g64660*) and *MOC3* (*Os04g56780)/TAB1/OsWUS* was delineated as reported by earlier studies^36–38^. Apart from monoculm genes, the tillering dwarf genes namely, D3 (*Os06g06050*)^39^, d27 (*Os11g0587000*, IRGSP-1.0)^40^, *HTD2/D88/D14* (*Os03g10620*)^41^ were observed to be effectively associated with the tiller numbers. Genes that regulate the plant hormone pathway namely *OsCKX2* (*Os01g10110*)^42^, *MIT3* (*Os11g36440*)^43^ and *OsPIN1* (*Os02g50960*)^44^ were observed to be involved in TN regulation. Negative regulator of axillary bud growth was identified to be an amino acid transporter gene, *OsAAP3* (*Os06g36180*)^45^ and gene *OsHAP2E* (*Os03g29760*) positively regulated the TN by increasing photosynthesis^46^. The genome wide association study (GWAS) associated with tiller number variations (LATNs) in rice identified four candidate genes *Os07g28890*, *Os11g15130*, *Os01g28690* and *Os05g32120* which might have a role in tiller number variation and are awaiting validation^35^. Another study used a combination of association studies and pedigree-based analysis for deciphering the genetic architecture of yield and grain quality using a MAGIC population^47^. This group identified pleiotropic interactions between grain yield (GYLD) and days-to-flowering (DTF) which was corroborated with co-localization of GYLD and DTF with *qDF3*/*OsMADS50*, flowering activator genes on chromosome 3.

To date, though the products of MAS had a significant impact in farmers' fields, the gap between identification of useful genes-QTLs and their potential use in breeding programs through MAS needs to be reduced. In recent years, significant efforts were made to reduce this gap by deploying novel technologies like next-generation sequencing (NGS) and single-nucleotide polymorphism (SNP) genotyping^48^. The present study was carried out with an objective to map novel genomic regions for the yield and its allied parameters with recombinant inbred line (RIL) population derived from an elite hybrid KRH-2 with the help of SSR and SNP markers.

## Materials and methods

### Plant material

#### Development of recombinant inbred line (RIL) population

Karnataka Rice Hybrid-2 (KRH-2) derived from the cross IR58025A × KMR-3R is a medium duration hybrid, with long-bold grain type with high yield potential and was developed by Zonal Agricultural Research Station (ZARS), Mandya, Karnataka. KRH-2 and its parents were used as the experimental materials. In dry season (*Rabi*) 2014, using the three line hybrid system, initial crosses were made between cytoplasmic male sterile (CMS) line, IR58025A and its maintainer line, IR58025B. Then, the IR58025A line was crossed with KMR-3R to produce the KRH-2 hybrid at Indian Institute of Rice Research, Hyderabad, India. Therefore, IR58025A was used as the female parent and all the RILs developed were iso-cytoplasmic lines. For the purpose of agro-morphological evaluation of the RIL population and its parents, IR58025B was used since IR58025A could not produce fertile seeds. The confirmed (true) F_1_ hybrid produced in dry season (*Rabi*) 2014 was self-pollinated to produce F_2_ seeds in wet season (*Kharif*) 2014. The F_2_ progenies were advanced till F_8_ generation through single seed descent (SSD) method^15^.

#### Phenotyping for yield and it’s allied traits

A total of 105 RILs derived from KRH-2 were used as mapping population for identification of yield related QTLs. 105 RILs along with the parents and hybrid KRH-2 were evaluated using randomized complete block design (RCBD) (each plot was of half acre in experimental field and 5 plants data was recorded from each line from each replication and the data was used for pooled analysis) and further statistical analysis. In each replication, five middle plants of each RIL entry were considered for phenotyping. Therefore, from two replications, ten plants were considered for agro-morphological evaluation. Phenotyping was recorded for three seasons viz., wet season 2016 (F_6_ generation), dry season 2017–2018 (F_7_ generation) and wet season 2017 (F_8_ generation). Twelve yield attributing traits were recorded using standard protocols^49^ from five plants of each RIL along with parents. Twelve yield related traits, viz., days to 50% flowering (DFF), total grain yield per plant (YLD), total number of grains per panicle (GP), fertile grains per panicle (FGP), 1,000 grain weight (TGW), panicle weight (PW), plant height (PH), panicle length (PL), flag leaf length (FLL), flag leaf width (FLW), productive tillers (PT), biomass (BM) were recorded in each of the three seasons mentioned above and the mean of the data collected was considered for analysis. Traits were recorded as follows: days to fifty percent flowering (DFF) was recorded as the number of days for initiation of flowering in 50% of plants from the date of sowing. Total grain yield/plant was measured by weighing the harvested grains of a single plant and was recorded in grams (g). Total number of grains per panicle (GP) was counted as a sum of filled and unfilled grains per panicle of a single plant. Fertile grains per panicle (FGP) were determined by counting the total number of filled grains per panicle of a single plant. Test (1,000) grain weight (TGW) was recorded as the weight of 1,000 filled grains per plant and was recorded in grams (g). Panicle weight (PW) was observed by taking the weight of the main panicle per plant and was measured in grams (g). Plant height (PH) was recorded at maturity stage and the height of a single plant was measured from the soil surface till the panicle tip of the main tiller. The trait was recorded in centimeters (cm). Panicle length (PL) was measured at the ripening stage from the neck of the panicle to the tip (excluding awn) and recorded in centimeters (cm). Flag leaf length (FLL) of the main tiller was measured at the beginning of anthesis and measured in centimeters (cm). Flag leaf width (FLW) of the main tiller of a single plant was measured at the beginning of anthesis and measured in centimeters (cm). Number of productive tillers (NPT) was determined at the harvest stage by counting the number of panicle bearing tillers per plant. Biomass (BM) was measured as the dry shoot–root weight per plant and was measured in centimeters (cm).

### Statistical analysis

The mean of the three seasons’ data (wet season 2016 (F_6_ generation), dry season 2017–2018 (F_7_ generation) and wet season 2017 (F_8_ generation) was considered for the statistical analysis. Frequency distribution histograms for all traits were generated using Statistical Tool for Agricultural Research (STAR) software v2.0.1, IRRI. Descriptive statistics or inferential statistical analysis was computed using SAS version 9.3 (SAS Institute Inc., Cary, NC, USA). Pr (predictor) value was estimated which is the p-value (probability value) associated with the F statistic of a given predictor or source (and the traits were considered as source which affected the outcome of QTL mapping). Various parameters of descriptive statistics namely standard deviation (SD), W value, predictor value (Pr), coefficient of variation in percentage (CV%), probability value (p-value), skewness and kurtosis were computed using SAS version 9.3 (SAS Institute Inc., Cary, NC, USA). Pooled combined analysis of variance (ANOVA) of three seasons’ agro-morphological data, genotypic-phenotypic correlation analysis and heritability estimates for twelve agro-morphological yield related characters under study was also done using SAS version 9.3 (SAS Institute Inc., Cary, NC, USA). Broad sense heritability was calculated as per the formula described in described in Ref.^50^.

$${\text{Broad sense heritability }}\left( {H^{2} } \right) = \frac{{\sigma^{{2}} {\text{G}}}}{{\sigma^{{2}} {\text{P}}}}$$

where σ^2^ G is the total genotypic variance and σ^2^ P is the total phenotypic variance.

### Genomic DNA extraction and genotyping of the RIL population

The genomic DNA was extracted from fresh and tender leaves of 105 RILs (F_6_ generation) using CTAB MiniPrep method^51^. The quality and quantity of DNA for each sample was checked on 0.8% agarose gel. DNA samples (40 ng) were amplified in 15 µl reaction volumes containing 1X PCR buffer [10 mM Tris–HCl (pH 8.3), 50 mM KCl, 1.5 mM MgCl_2_, 0.01% (v/v) gelatin], 0.2 mM of each dNTPs, 5 pmol of each primer and 1U of Taq polymerase (Bangalore Genei, India). The PCR condition for all primers was same at all stages such as 5 min at 94 °C of initial denaturation, 35 cycles of 30 s at 94 °C (Denaturation), 30 s at 55 °C ± 2 °C (Annealing), 1 min at 72 °C (Extension) and 5 min at 72 °C for final extension. The amplification products were size fractioned in a 4% agarose gel (Sigma, USA) in a Protean II gel casting and electrophoresis apparatus (BioRad, USA), stained in 0.5 µg/ml ethidium bromide, visualized under ultraviolet light and was documented in a gel documentation system (Alpha Innotech, USA). A total of 1,904 genomic SSRs^52^ (https://www.gramene.org) were screened for parental polymorphism survey between IR58025A and KMR-3R. Among the 1,904 SSR markers tested for parental polymorphism, 132 markers were identified as polymorphic among the parents and used for genotyping the population. Six markers namely RM13616 (chromosome 2), RM6997 (chromosome 4), RM17377 (chromosome 4), RM18614 (chromosome 5), RM19291 (chromosome 6), RM23861 (chromosome 9) were identified with segregation distortion using segregation distortion loci (SDL) mapping function of ICIM. These six distorted markers (4.5% of the total 132 polymorphic markers) were not used in further linkage map construction and QTL mapping. The remaining 126 SSRs with no segregation distortion were only used for the QTL mapping. Those fragments which had unambiguous and clear amplification were considered for scoring with 50 bp ladder. A score of 1 was assigned to each marker for the presence of specific allele and a score of 0 for its absence. A binary data matrix was thus generated for all alleles at each locus of 126 polymorphic markers.

### Construction of SSR and SNP based genetic linkage map

JoinMap software v. 4.0^53^ was used for construction of genetic linkage map. The markers with maximum missing data were filtered to maintain the quality of map. Distorted markers which failed in chi-square test were also removed to avoid the noise during map construction. After stringent filtration, a genetic map was constructed using high quality genotyping data generated based on analysis using 126 SSR markers among the 105 RILs. Kosambi map function was used to convert the recombination frequency into map distance (cM).

A total of 1,882 filtered SNPs were analyzed for their association with 12 agro-morphological traits under study using the TASSEL GBS pipeline^54^ among the 12 high and 12 low yielding RILs (two extreme yielding groups of the RIL population). Further, a total of 800 redundant-filtered SNPs were observed which were omitted. The data matrix of filtered and non-redundant SNP allelic changes among the 12 high and 12 low yielding RILs generated through genome wide association studies (GWAS) consisted of homozygotic biallelic transitions-transversions along with heterozygotic biallelic transitions-transversions. All homozygotic biallelic nucleotides were given a letter code of A and the heterozygotic bialleic nucleotides were replaced with letter B. The missing nucleotides were represented with an asterisk (*) symbol. The data matrix thus generated with A and B letter codes was used for the construction of linkage QTL map using the inclusive composite interval mapping (ICIM) method with 1,082 SNPs and 24 selected RILs.

### Linkage QTL map construction and QTL mapping

Inclusive composite interval mapping method (ICIM) was used for the construction of SSR and SNP based linkage QTL map using QTL IciMapping software ver. 4.0.1^55,56^ (https://www.isbreeding.net). For both QTL mapping, the Kosambi map function was used and the number of permutations for the determination of significance levels (at p = 0.05) was set to 1,000 and the LOD threshold was set to 2.5. At significant LOD peak (i.e., at greater than or equal to 2.5), QTL mapping in terms of QTL effects namely the log-likelihood ratio (LOD), additive effect of the identified loci and phenotypic variation explained (PVE) were estimated. The LOD test statistic used was − 2ln (L_0_/L_1_), where L_0_/L_1_ is the ratio of the likelihood under the null hypothesis (indicating the absence of QTL) and the alternative hypothesis (indicating the presence of QTL)^57^. Those QTLs whose phenotypic variation percentage (PVE%) was more than 20% were termed as major effect QTLs^58^. QTL nomenclature was followed as described by Ref.^59^. Identification of QTL hotspots was done manually as described in Ref.^4^ but with slight modifications. The genomic regions linked to QTL was searched for QTL hotspots in a sliding window size of 20 cM and the regions with two or more than two co-locating QTLs in each window region was identified. Construction of linkage map, QTL mapping, identification of digenic (epistatic) interactions between a pair of SSR-SNP marker loci and QTL by environment interaction in the population using the MET functionality were determined using QTL IciMapping software ver. 4.0.1^55,56^. SSR and SNP markers based QTLs with quantitative epistatic interactions (QEIs) with LOD thresholds of 3.0 were detected. The window size was set at 10 cM and in order to determine the cofactors, stepwise regression analysis was used. QTL main effects were estimated using the maximum-likelihood estimation method. For the 12 traits under study, the LOD threshold was determined at the experiment-wise significance level of 0.05 by computing 1,000 permutations^60^ and the LOD threshold ranged from 2.5–3.3. SSR and SNP markers based QTLs identified from this study were compared to those identified in previous studies using the Gramene QTL database (https://archive.gramene.org/qtl/) and QTARO database (https://qtaro.abr.affrc.go.jp/qtab/table#as_table:21:undefined:undefined). Further, the QTLs were termed as novel if the observed marker intervals did not overlap significantly with marker intervals reported in earlier studies^4^. For in silico analysis, the putative candidate gene (s) were identified with respect to common and novel QTLs detected among 105 RILs (analyzed with SSRs) and also 24 selected RILs (analyzed with SNPs) using the Rice Annotation Project Database (RAP-DB) (https://rapdb.dna.affrc.go.jp/) and putative functions of candidate gene (s) located in the genomic regions spanning the QTLs associated with the traits were identified.

### Genotyping-by-sequencing (GBS) for SNP genotyping of the selected RILs

On the basis of total grain yield/plant (YLD) for three consecutive seasons, 12 high and 12 low yielding RILs were selected in F_7_ generation for genotyping-by-sequencing (GBS)^61^ to identify single nucleotide polymorphism (SNP) variants. For this, 10 ng DNA from each selected RIL was digested using the restriction enzyme *Pst*I endonuclease which cuts at the site: CTGCA/G and generates the fragments with 3′ cohesive ends and yield four base pair cohesive ends. T4 ligase enzyme was used to ligate the digested samples with uniquely barcoded adapters. DNA from each RIL was digested and ligated with individual adapters, and then, equal proportion from each sample was pooled to construct the libraries. These libraries were then amplified and purified to remove excess adapters. The DNA libraries were then sequenced on HiSeq 2500 platform (IlluminaInc, San Diego, CA, USA) to generate genome-wide sequence reads.

### SNP calling and filtering

Sequenced raw reads were generated for each sample in the form of FASTQ format and imported in TASSEL GBS pipeline implemented in TASSEL Version 5.2.0^54^, Os-Nipponbare-Reference-IRGSP-1.0^62^ (https://rice.plantbiology.msu.edu/) was used as a reference genome for mapping the reads. The barcode information was used for de-multiplexing the reads. The sequence reads which passed quality filtering criteria were mapped on draft genome sequence of rice using BWA (Burrows-Wheeler Alignment) tool^63^. The mapped reads were exported in the form of Sequence Alignment Map (SAM) file and processed for SNP calling and genotyping. Total of 10.3 Gb data was generated for 24 RILs with minimum coverage of 0.05X. Generated raw reads for each individual recombinant inbred line were processed for SNP calling in TASSEL. Total of 1, 882 SNPs were generated using low-throughput genotyping by sequencing platform non Illumina Hiseq 2500. The SNPs with minor allele frequency (MAF) of ≤ 0.3 were filtered out to avoid the noise during association analysis. The SNPs which are having more than 50% of missing data (across all the chromosomes) were filtered out. The remaining SNPs (which are reasonably spread across the entire rice genome), which are having very low percentage of missing data were imputed in FSFHap implemented in TASSEL V 5.2.0. The total missing data in the analyzed SNP was only to an extent of 8.34%. After completion of GWAS, 1,882 SNPs (spread across all chromosomes) showed association with yield and allied traits. Hence, the SNPs which were not associated with the traits and which were redundant were filtered out again for fine tuning of analysis. Therefore, finally after stringent filtration, 1,082 SNPs were used for the construction of genetic map.

### Genome-wide marker-trait association analysis using SNP markers

Association mapping analysis was performed with TASSEL V5.2.0 software^54^ using General Linear Model (GLM) method. For GLM, the model with no control for population structure and relatedness was performed^64^. The general equations for GLM is y = Xa + e where, ‘y’ is vector for phenotypes; ‘a’ is the vector of marker fixed effects, and e is the vector of residuals. ‘X’ denotes the genotypes at the marker. Marker alleles with P-values ≤ 0.001 in model was stated significantly associated SNPs with yield component traits^65^. Significantly associated SNPs with major effect QTLs (identified through SSR markers) was checked with the reported QTL/gene information in QTARO database (https://qtaro.abr.affrc.go.jp/qtab/table#as_table:21:undefined:undefined) based on the SNP’s physical position for knowing the implications of identified SNP loci in governing various traits other than the associated trait.

### Percent missing data among the SSR-EST derived SSRs and SNP markers

For determining the percent of missing data using the SSR markers, total number of data-points that were supposed to be amplified with 126 SSRs in RIL population was determined based on the number of genotypes under study. Among them, the number of data-points that did not show any amplification was noted. The percent of missing data was calculated as ratio of the number of unamplified data-points to that of total number of data-points expected to amplify. The value thus obtained was multiplied with 100 and was expressed in percentage. A similar method was followed for determining the percentage of missing data with SNP markers.

### Identification of efficient restorers among the RILs

Apart from the utility of the RIL population in QTL mapping and in order to identify efficient restorer lines among the 105 RILs, genomic DNA was extracted from fresh and tender leaves of RIL population along with IR58025A, IR58025B, KMR-3R and KRH-2 by following the protocol^51^ and amplified using the functional markers, RMS-SF21-5 and RMS-PPR9-1, which are specific for the major fertility restorer genes, *Rf3* locus and *Rf4* locus, respectively^66^. A set of 12 high-yielding RILs which were semi-tall (plant height of 110 ± 5 cm) and tall (plant height of 125 ± 5 cm) with respect to plant stature were selected for test-crossing for analysis of their fertility restoration. These lines were validated for their fertility restoration potential (%) through test crossing with the WA-CMS line, IR58025A as per the crossing methodology^67^ in dry season 2018–2019. The novel F_1_ hybrids derived from these test crosses were raised in the wet season of 2018 and assessed for twelve yield attributing traits (viz., DFF, YLD, GP, FGP, TGW, PW, PH, PL, FLL, FLW, PT, BM) were recorded using standard protocols^49^ from five healthy plants of each of the hybrid along with their parents at IIRR, Hyderabad. The lines were categorized as potential complete restorers if the F_1_’s fertility percentage was more than 70%^68^. Selection efficiency of functional markers for *Rf3* and *Rf4* loci was analyzed with a set of above mentioned 12 high-yielding RILs which were identified as complete and partial restorers (through test cross).

## Results

### Statistical analysis on trait performance of RILs

**Figure 1:**
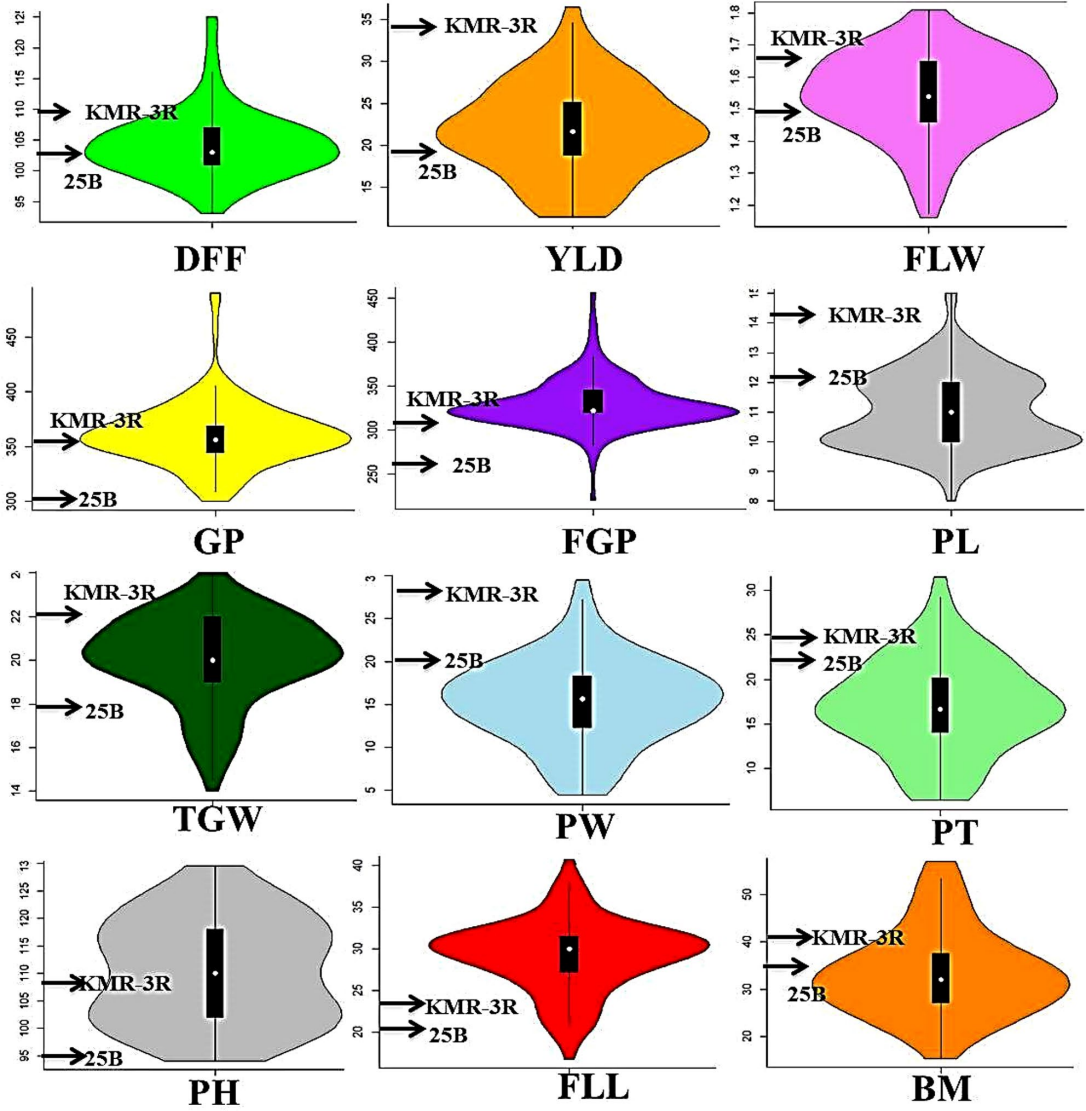
Frequency distribution violin plots of phenotypic data observed in RIL population for twelve important yield related traits. The values of parents, IR58025B (25B) and KMR-3R is indicated with arrows for traits days to fifty percent flowering (DFF), total grain yield per plant (YLD, g), total number of grains per panicle (GP), fertile grains per panicle (FGP), 1,000 grain weight (TGW), panicle weight (PW), plant height (PH), panicle length (PL), flag leaf length (FLL), flag leaf width (FLW), productive tillers (PT), biomass (BM). The X-axis represents the traits and the Y-axis constitutes the range of values for frequency distribution for every trait. R package version 0.3.4; https://cran.r-project.org/web/packages/vioplot/.

Frequency distribution diagrams and histograms of all 12 traits indicate their normal distribution across three seasons at one location (Fig. 1, Supplementary Fig. 1). A wide range of higher phenotypic variation (Supplementary Table 1) was observed among 105 RILs for all the traits.

**Table 1:** Putative major effect QTLs for agronomic traits in 105 recombinant inbred line (RIL) population derived from the cross of IR58025A × KMR-3R (KRH-2).

Trait^a^	QTL^b^	Flanking markers		Chr	Start position (cM)	End position (cM)	QTL size (cM)	LOD peak^c^	PVE(%)^d^	Add^e^	RSq^f^
YLD	*qYLD3-1*^ g^	RM517	RM15679	3	113.51	141.48	27.97	10.59	22.76	− 6.44	32.92
PL	*qPL3-1*^ g^	RM517	RM15679	3	113.51	141.48	27.97	10.47	22.70	− 6.14	31.57
PW	*qPW3-1*	RM15679	JGT03-26.8	3	141.48	144.51	3.03	8.25	20.81	− 5.59	35.01
FLW	*qFLW4-1*	RM6909	RM252	4	68.47	69.94	1.47	6.50	20.23	− 0.13	18.26
PH	*qPH12-1*	RM27404	RM28275	12	0	32.80	32.8	7.01	22.54	− 5.82	30.36

^a^*YLD *total grain yield/plant(g), *PW* panicle weight (g), *PH* plant height (cm), *FLW* flag leaf width (cm), *PL* panicle length (cm).

^b^Designation of the QTLs is in accordance with the rules recommended by Ref.^22^.

^c^Logarithm of the odds ratio (LOD) score of ≥ 2.5 was set as threshold for this data.

^d^Total phenotypic variance (PVE%) in percentage explained by the QTL.

^e^Additive effect-Negative additive effect value indicates the direction of favorable allele from donor parent, KMR-3R, that increased the trait value.

^f^Phenotypic variation explained by the final regression model.

^g^QTL hotspots on chr 3 for total grain yield/plant (g) and panicle length (cm).

High broad-sense heritability (*H*^2^) was observed for all the traits with a range of 92.66–98.44% except biomass (38.99%), in the RIL population (Supplementary Table 1). The combined Analysis of Variance (ANOVA) demonstrated high significant differences among the RILs for all the traits under study (viz., DFF, YLD, GP, FGP, TGW, PW, PH, PL, FLL, FLW, PT, BM) as shown in Supplementary Table 2. The statistical variation as descriptive statistics in agro-morphological data is presented in Supplementary Table 3. Phenotypic-genotypic correlation between total grain yield/plant (YLD) and its associated traits are presented in Supplementary Table 4. A very strong and positive phenotypic correlation was observed between total grain yield/plant (YLD) and panicle weight (PW) (r = 0.89) and plant height (PH) (r = 0.35) at 1% level of significance. Similarly, the traits total grains/panicle (GP), fertile grains/panicle (FGP), test grain weight (TGW), flag leaf length (FLL), flag leaf width (FLW), number of productive tillers (PT), panicle length (PL) showed the positive and significant phenotypic correlation at 5% level of significance with total grain yield/plant (YLD) whereas negative correlation was observed between YLD and DFF (r = − 0.24 (phenotypically) (r = − 0.49 (genotypically). As compared to phenotypic correlation, the genotypic correlation was very strong and positive between YLD and most of the traits namely TGW (r = 0.41), PW (r = 0.85), PH (r = 0.62), FLL (r = 0.52), FLW (r = 0.44), PT (r = 0.42), PL (r = 0.85) were strongly correlated at 1% level of significance. Trait GP showed a positive correlation (r = 0.21) with YLD at 5% level of significance. Correlation was observed to be positive between YLD and BM, r = 0.15 (genotypic) and r = 0.22 (phenotypic) only. Significant and positive correlation coefficients were observed between the trait YLD and for most of its important allied components namely PW, GP, FGP, PT and PL. Moreover, the correlation between the agro-morphological traits in subset population consisting of 24 selected RILs is presented in Supplementary Table 5. Positive and highly significant correlation was observed between the traits, YLD and GP (r = 0.27), FGP (r = 0.33), PW (r = 0.83), FLW (r = 0.56), PT (r = 0.39) and PL (r = 0.88) at 1% level of significance whereas TGW (r = 0.43) was observed to be positively correlated with YLD trait at 5% level of significance. Negative correlation was observed between YLD and DFF (r = -0.51) at 5% level of significance.

**Table 2:** SNP allelic changes identified in major effect QTLs.

Major effect QTLs identified in 105 RILs						p-value	R^2^ value (%)	High yielding RILs						Low yielding RILs					
QTL name	Flanking SSR markers	SSR Marker position (Mb)	Name of the SNP marker	SNP marker position (Mb)	SNP allelic change			RIL-1 and 2	RIL-3 and 4	RIL-5 and 6	RIL-7 and 8	RIL-9 and 10	RIL-11 and 12	RIL-13 and 14	RIL-15 and 16	RIL-17 and 18	RIL-19 and 20	RIL-21 and 22	RIL-23 and 24
*qYLD3-1*	RM517-RM15679	6.1–26.8	S3_27516811	27.51	A/G	0.02	40.3	AA	AA	AA	AA	AA	AA	GG	GG	GG	GG	GG	GG
*qPW3-1*	RM15679-JGT03-26.8	26.8	S3_27516811	27.51	A/G	0.14	20	AA	AA	AA	AA	AA	AA	GG	GG	GG	GG	GG	GG
*qPL3-1*	RM517-RM15679	6.1–26.8	S3_27516811	27.51	A/G	0.02	40.3	AA	AA	AA	AA	AA	AA	GG	GG	GG	GG	GG	GG
*qFLW4-1*	RM6909-RM252	32–24	S4_24002530	24	C/T	0.02	50.7	CC	CC	CC	CC	NN	CC	TT	TT	TT	TT	TT	TT
*qPH12-1*	JGT12-0.2-RM28275	0.2–19.7	S12_14867709	14.86	G/A	0.04	44.8	GG	GG	GG	GG	NN	GG	AA	AA	AA	AA	AA	AA

*N* any nucleotide, *YLD* total grain yield/plant(g), *PW* panicle weight (g), *PH* plant height (cm), *FLW* flag leaf width (cm), *PL* panicle length (cm), *R*^2^* value (%)* the cumulative phenotypic variance (%).

### QTL mapping using SSR markers

Using 126 hyper-variable SSR markers (Supplementary Table 6), a total of 22 major and minor effect QTLs were identified with 105 RILs for all the traits except for the traits flag leaf length (FLL) and number of productive tillers (PT) (Table 1 and Supplementary Table 7). Collinearity between the genetic (cM) and physical maps (bp) of all chromosomes is shown in Supplementary Fig. 3. A total of five major effect QTLs were identified for the traits viz., total grain yield/plant (YLD), panicle weight (PW), panicle length (PL), flag leaf width (FLW) and plant height (PH) on chromosomes 3, 3, 3, 4 and 12, respectively. The logarithm of the odds ratio (LOD) peak/score values of these QTLs were in the range of 6.50 to 10.59 whereas the phenotypic variation under the influence of these QTLs was in the range of 20.23–22.76%. A QTL was considered as major effect when the phenotypic variation explained in percentage (PVE%) was more than 20%^58^ (Table 1). The details of the major QTLs are presented below.

#### Total grain yield/plant (YLD)

**Figure 2:**
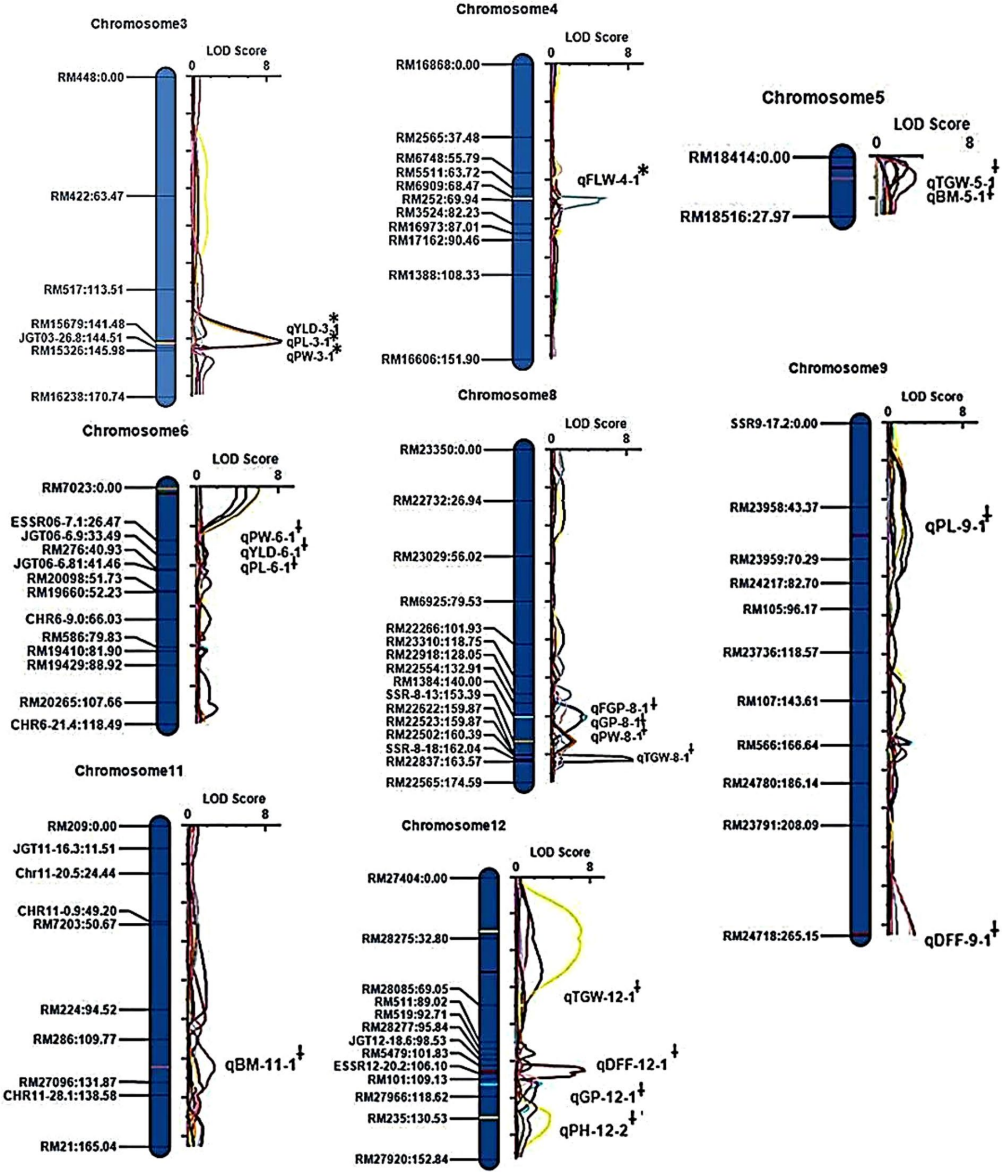
QTL-marker linkage map of selected chromosomes along with the identified QTLs when QTL mapped with 105 RILs and 126 SSR markers. The names and position of markers in centi morgan (cM) are given on left side and LOD scores with peaks are presented on right side of chromosomes. Major effect QTLs are denoted with asterisk (*) and minor effect QTLs are denoted with Ɨ symbol.

The major QTL, *qYLD3-1*, was identified with LOD score of 10.59 and showed a percentage of phenotypic variation (PVE%) of 22.76%. The size of this QTL was 27.97 centi Morgan (cM) within flanking hyper-variable SSR markers, RM517 (6.13 Mb) and RM15679 (26.87 Mb). Among the two markers, the closest associated marker to the QTL was RM15679. This QTL may have augmented trait value of grain yield/plant by 6.44 g as the favorable allele was from KMR-3R (Table 1, Fig. 2, Supplementary Fig. 2 (I)). Cumulative effect (RSq value) of major and minor effect QTLs is presented in Table 1, Supplementary Table 5. The nearest SNP marker named S3_27516811, associated with, *qYLD3-1*, was identified at 27.51 Mb with p-value of 0.02. The SNP allelic change underlying this QTL among the high and low yielding groups was a transition from adenine (A) nucleotide base to guanine (G), respectively (Table 2).

#### Panicle length (PL)

A major QTL, *qPL3-1,* which was 27.97 cM in size and flanked by the SSR markers, RM517 (6.13 Mb) and RM15679 (26.87 Mb) was identified. The closest associated marker to this QTL was RM15679. This QTL showed a LOD of 10.47 and the PVE% was 22.70. As the additive effect (Add) value was computed to be − 6.14, the trait enhancing allele was from KMR-3R, and contributed in increasing the panicle length by 6.14 cm over IR58025A (Table 1, Fig. 2, Supplementary Fig. 2 (I)). The closest SNP marker, S3_27516811, associated with this QTL was identified at 27.51 Mb with lowest p-value of 0.02. Genetic transition from adenine (A) to guanine (G) was the SNP allelic change underlying PL trait QTL as shown in Table 2.

#### Panicle weight (PW)

*qPW3-1*, a major effect QTL for panicle weight trait was identified with LOD score of 8.25 and PVE% of 20.81. The size of this QTL was 3.03 cM and the associated with flanking hyper-variable SSR markers were RM15679 (26.87 Mb) and JGT03-26.8 (26.8 Mb). Additive effect (Add) of *qPW3-1* was − 5.59 demonstrating the direction of favorable allele from KMR-3R (Table 1, Fig. 2, Supplementary Fig. 2 (II)). The nearest SNP marker, S3_27516811 was associated with this QTL with a lowest p-value of 0.14 and was identified at 27.51 Mb with genetic transition from adenine (A) to guanine (G) SNP allelic change (Table 2).

#### Flag leaf width (FLW)

A 1.47 cM sized major QTL for flag leaf width (FLW), *qFLW4-1,* was identified between hyper-variable SSR markers, RM6909 (32.09 Mb) and RM252 (24.02 Mb). The LOD peak was at 6.50 with PVE% of 20.23%. Interestingly, the allele for trait, flag leaf width was observed to be inherited from IR58025A (Table 1, Fig. 2, Supplementary Fig. 2 (III)). The closest associated SNP marker was S4_24002530 at 24 Mb with lowest p-value 0.02. The SNP allelic change observed was a genetic transition from cytosine (C) to thymine (T) among the high and low yielding RILs, respectively (Table 2).

#### Panicle weight (PW)

*qPW3-1*, a major effect QTL for panicle weight trait was identified with LOD score of 8.25 and PVE% of 20.81. The size of this QTL was 3.03 cM and the associated with flanking hyper-variable SSR markers were RM15679 (26.87 Mb) and JGT03-26.8 (26.8 Mb). Additive effect (Add) of *qPW3-1* was − 5.59 demonstrating the direction of favorable allele from KMR-3R (Table 1, Fig. 2, Supplementary Fig. 2 (II)). The nearest SNP marker, S3_27516811 was associated with this QTL with a lowest p-value of 0.14 and was identified at 27.51 Mb with genetic transition from adenine (A) to guanine (G) SNP allelic change (Table 2).

#### Flag leaf width (FLW)

A 1.47 cM sized major QTL for flag leaf width (FLW), *qFLW4-1,* was identified between hyper-variable SSR markers, RM6909 (32.09 Mb) and RM252 (24.02 Mb). The LOD peak was at 6.50 with PVE% of 20.23%. Interestingly, the allele for trait, flag leaf width was observed to be inherited from IR58025A (Table 1, Fig. 2, Supplementary Fig. 2 (III)).The closest associated SNP marker was S4_24002530 at 24 Mb with lowest p-value 0.02. The SNP allelic change observed was a genetic transition from cytosine (C) to thymine (T) among the high and low yielding RILs, respectively (Table 2).

#### Plant height (PH)

Between the flanking SSR markers, RM27404 (0.20 Mb) and RM28275 (19.47 Mb), a major QTL, *qPH12-1*, of 32.80 cM size, was identified on chromosome 12 for trait plant height with LOD peak value of 7.01, and PVE% value of 22.54%. RM28275 was the nearest associated marker with this QTL. The direction of the favorable allele was identified to be inherited from KMR-3R, with additive effect value of − 5.82 (Table 1, Fig. 2, Supplementary Fig. 2 (IV)). The nearest associated SNP marker was S12_14867709 at 14.86 Mb with lowest p-value of 0.04. Guanine (G) to Adenine (A) was the SNP allelic change identified with this QTL among the high and low yielding RILs (Table 2).

A total of 17 minor effect QTLs were identified for various traits. A brief account of these QTLs is presented herewith. One minor effect QTL each for the traits, total grain yield/plant (*qYLD6-1*), fertile grains per panicle (*qFGP8-1)*, plant height (*qPH12-2*), flag leaf width (*qFLW4-2*) trait were identified. Also, two QTLs were identified for each of the listed traits: days to fifty percent flowering (*qDFF9-1* and *qDFF12-1*), total grains per plant (*qGP8-1* and *qGP12-1*), panicle weight (*qPW6-1* and *qPW8-1*), panicle length (*qPL6-1* and *qPL9-1*), biomass (*qBM5-1* and *qBM11-1*). Three QTLs for trait test grain weight (*qTGW5-1*, *qTGW8-1*, *qTGW12-1*) were identified.

Details of these minor effect QTLs is presented in Supplementary Table 7. The details of co-localization of these minor effect QTLs identified in this study with previously reported QTLs are presented in Supplementary Table 8. The details of individual SNP allelic changes underlying the minor effect QTLs is presented in Supplementary Table 16. A brief account on the involvement of identified SNP loci associated with minor effect QTLs in regulating different traits is presented in Supplementary Table 17.

Epistatic interactions for YLD, PL traits among chromosomes 3, 4, 5, 7 are shown in Supplementary Fig. 4 (I) and (II). Three digenic (epistatic) interactions for two traits namely total grain yield/plant (YLD) and panicle length (PL) were observed. Major effect YLD epistatic interactions on chromosome 3 were observed between two pairs of flanking markers RM517-RM15679 and RM21992-RM22171. In this interaction, the PVE% was 20.16%, with LOD peak value of 7.38 and cumulative effect of this QTL interaction was 32.90%. The additive by additive effect value of the interaction was observed to be − 5.36 (as shown in Supplementary Table 9. Secondly, two major effect epistatic interactions were observed on chromosome 3 for panicle length (PL). The epistatic interaction 1 was observed between two pairs of markers namely RM448-RM422 and RM7023-ESSR06-7.1, between chromosomes 3 and 6, respectively. The PVE of this interaction was observed to be 20.38%, the additive by additive interaction was recorded as 2.26. Another epistatic interaction was observed between two pairs of RM markers namely RM517-RM15679 and RM21992-RM22171 between chromosomes 3 and 7, respectively. The percentage of phenotypic variation explained (PVE%) was 29.73 with negative additive by additive effect value of 4.04. The cumulative effect (RSq) of both epistatic interactions on the trait value was 31.57% (Supplementary Table 9).

Supplementary Table 10 describes the QTL × environment interaction in three consecutive seasons namely wet season 2016, dry season 2017 and wet season 2017 for all the major-minor effect QTLs. Parameters namely LOD (AbyE)-LOD score for additive by environment effects; RSq-Phenotypic variation explained by the final regression model, PVE(AbyE)-Phenotypic variation explained by additive by environment effect at the current scanning position, AbyE_01, 02 and 03-Additive by environment effect were observed to determine the genotype × environment interactions. Pertaining to the major effect QTLs, the LOD (AbyE), PVE (AbyE) and AbyE were estimated across three seasons and was observed as zero. There was no significant variation in the observed RSq values for all the major effect QTLs. Similar effects were also observed with minor effect QTLs.

### QTL mapping using SNP genotyping

Using 1,082 non-redundant-filtered SNP markers (Supplementary Table 19) and 24 selected RILs (i.e. 12 high yielding RILs and 12 low yielding RILs), a total of 26 major and minor effect QTLs were identified for all the traits except for GP, FLW, PT and BM (Supplementary Table 11, Supplementary Fig. 5). Also, eight novel QTLs were identified for the traits YLD, DFF, PW, PL, and FLL. A total of ten major effect QTLs identified are presented below.

Total grain yield/plant (YLD): A major QTL, *qYLD2-1* of size 2.87 Mb and flanked by the SNP marker interval S2_5359418 (5.35 Mb) and S2_8229921 (8.22 Mb) with an LOD score of 10.82 and PVE% of 30.75% was identified. The additive effect of this QTL indicated its direction of inheritance from IR58025A. The cumulative phenotypic variance (RSq) of major-minor effect YLD QTLs on the trait explained up to 81.56%.

#### Test grain weight (TGW)

A 0.16 Mb sized, QTL,*qTGW11-1,* located between the SNP markers, S11_1125465 (1.12 Mb) and S11_965658 (0.69 Mb) was identified on chromosome 11 with LOD score of 3.36, PVE% of 29.47% and additive effect value of 2.57 indicating its direction from IR58025A. The cumulative phenotypic variance (RSq) of this QTL on TGW trait was up to 74.56%.

#### Panicle weight (PW)

Two major effect QTLs namely, *qPW2-1* and *qPW10-1* were identified. *qPW2-1*, flanked between SNP markers S2_5359418 (5.35 Mb)-S2_8229921 (8.22 Mb) on chromosome 2, was observed to have an LOD score of 6.49, PVE% of 52.88% and additive effect value of 6.21 demonstrating its inheritance from IR58025A with an RSq value of 74.35%. *qPW10-1*, a 11.07 Mb sized QTL, was identified on chromosome 10 in between SNP markers, S10_20693837 (20.69 Mb) and S10_9627559 (9.62 Mb). The details of this QTL are as follows: LOD score (5.97), PVE% (31.77%), additive effect value (-4.63) indicating its direction from KMR-3R. The cumulative effect of both the QTLs on the trait (RSq value) was observed to contribute up to RSq (74.35%).

#### Panicle length (PL)

Two major effect QTLs, namely, *qPL2-1* and *qPL10-1* were identified for PL trait. *qPL2-1*, identified on chromosome 2, was observed to be flanked by the SNP markers S2_5359418 (5.35 Mb)-S2_8229921 (8.22 Mb) with an LOD score of 17.81, PVE%-61.75% and additive effect value of 8.06 indicating its direction of inheritance from IR58025A. The QTL *qPL10-1* QTL was identified flanking in between the SNP markers, S10_20693837 (20.6 Mb), S10_9627559 (9.6 Mb). This QTL of size 11.07 Mb was observed with an LOD score of 14.45, with PVE% of 29.94% and additive effect value of − 7.10 indicating its direction of inheritance from KMR-3R. The cumulative effect of all the major and minor effect QTLs (RSq value) was observed to contribute up to 96.74%.

#### Plant height (PH)

A 2.72 Mb sized QTL, *qPH2-1* was identified on chromosome 2, flanked the SNP markers, S2_5359418 (5.35 Mb) and S2_8229921 (8.22 Mb) with an LOD score of 2.72, phenotypic variance explained in percentage up to 42.71%, additive effect value of 4.49 indicating its direction from IR58025A with an RSq value of 68.69%.

#### Flag leaf length (FLL)

A major QTL for FLL trait, *qFLL6-1*, identified on chromosome 6, was observed to have a LOD score of 2.86 and was flanked by the SNP markers, S6_87110 (0.08 Mb) and S6_56355 (0.05 Mb). The PVE% of this QTL was observed to be 42.38% with an additive effect value of 2.81 indicating its direction from IR58025A. The RSq value of this QTL was observed up to 39.66%.

A correlation was observed between SNP allelic changes observed in 12 high and 12 low yielding RILs (identified through GBS) and major effect QTLs identified using 126 SSR markers in 105 RILs.

#### Fertile grains per plant (FGP)

Two major effect QTLs for FGP trait, namely, *qFGP5-1* and *qFGP5-2* were identified through mapping using SNP markers. *qFGP5-1* was flanked in between the SNP markers, S5_29807077 (29.8 Mb) and S5_19215973 (19.21 Mb) with LOD score of 2.51, PVE% of 35.51% with an additive effect value of − 16.07 indicating its direction from KMR-3R. *qFGP5-2*, flanked in between SNP markers, S5_3361011 (3.36 Mb) and S5_29950375 (29.95 Mb) with LOD score of 2.80, PVE%-42.22% and additive effect value of 16.22 indicating its inheritance from IR58025A. The cumulative effect (RSq value) of both these QTLs on the trait was observed to contribute up to 64.54%.

A total of 15 minor effect QTLs were observed for two traits, YLD and DFF (Supplementary Table 11). Nine QTLs namely, *qYLD1-1, qYLD2-2, qYLD2-3, qYLD3-1, qYLD4-1, qYLD6-1, qYLD8-1, qYLD10-1, qYLD10-2* for trait total grain yield per plant (YLD) were observed. For DFF trait, a total of six QTLs namely, *qDFF2-1, qDFF3-1, qDFF3-2, qDFF4-1, qDFF6-1, qDFF6-2* among which *qDFF2-1* and *qDFF4-1* were novel QTLs. For panicle length trait, a novel QTL, *qPL3-1*, was identified.

A total of six major effect epistatic interactions were observed for traits: test grain weight, panicle weight, flag leaf length and flag leaf width (Supplementary Table 13, Supplementary Fig. 6 (I)–(II)). For the trait TGW, two epistatic interactions were observed whose LOD scores were 5.17 and 5.75, with PVE% of 25.06% and 25.52%, respectively. The cumulative effect of these interactions of the trait was observed up to 61.48% (RSq value). For the trait panicle weight, two interactions were observed with LOD scores of 5.01 and 5.09. The PVE% of these interactions was 43.52% and 29.36%, respectively with RSq value explaining up to 74.35% of the phenotypic variance. For trait flag leaf length, epistatic interaction was observed in between chromosome 6 and chromosome 12 with an LOD score of 5.34 whose phenotypic variance was explained up to 38.84% with an RSq value of 39.66%. For trait, flag leaf width, an epistatic interaction was observed between chromosome 5 and chromosome 12 with an LOD score of 6.93, with phenotypic variance accounting up to 32.73% and RSq value of 52.86%.

### Consistent QTLs with SSR and SNP markers

Two QTLs namely *qYLD3-1, qPL3-1* were detected in common for two traits namely total grain yield per plant and panicle length, respectively, with 105 RILs-126 SSRs and 24 selected RILs-1, 082 SNPs (Supplementary Table 12). Based on mapping with SSR markers the *qYLD3-1*, originally was identified to be of 20.74 Mb but through SNP marker-based mapping, the interval was narrowed down to 7.34 Mb. Negative additive effect was observed for this QTL through mapping with both SSR and SNP markers. High cumulative phenotypic variation (RSq value) of 81.65% was observed, when the QTL mapped with SNP markers, whereas with SSR markers, the RSq value was 32.92%. The size of *qPL3-1* which was originally of 20.74 Mb when mapped with SSR markers was narrowed down to 0.81 Mb, when mapped with SNP markers. As observed with total grain per plant QTL, negative additive effect was observed for *qPL3-1* with very high cumulative phenotypic variation of 96.74% in the mapping exercise with SNP markers, while it was only 31.57% when mapped with SSR markers.

### In-silico analysis for putative candidate gene (s) identification

**Figure 3:**
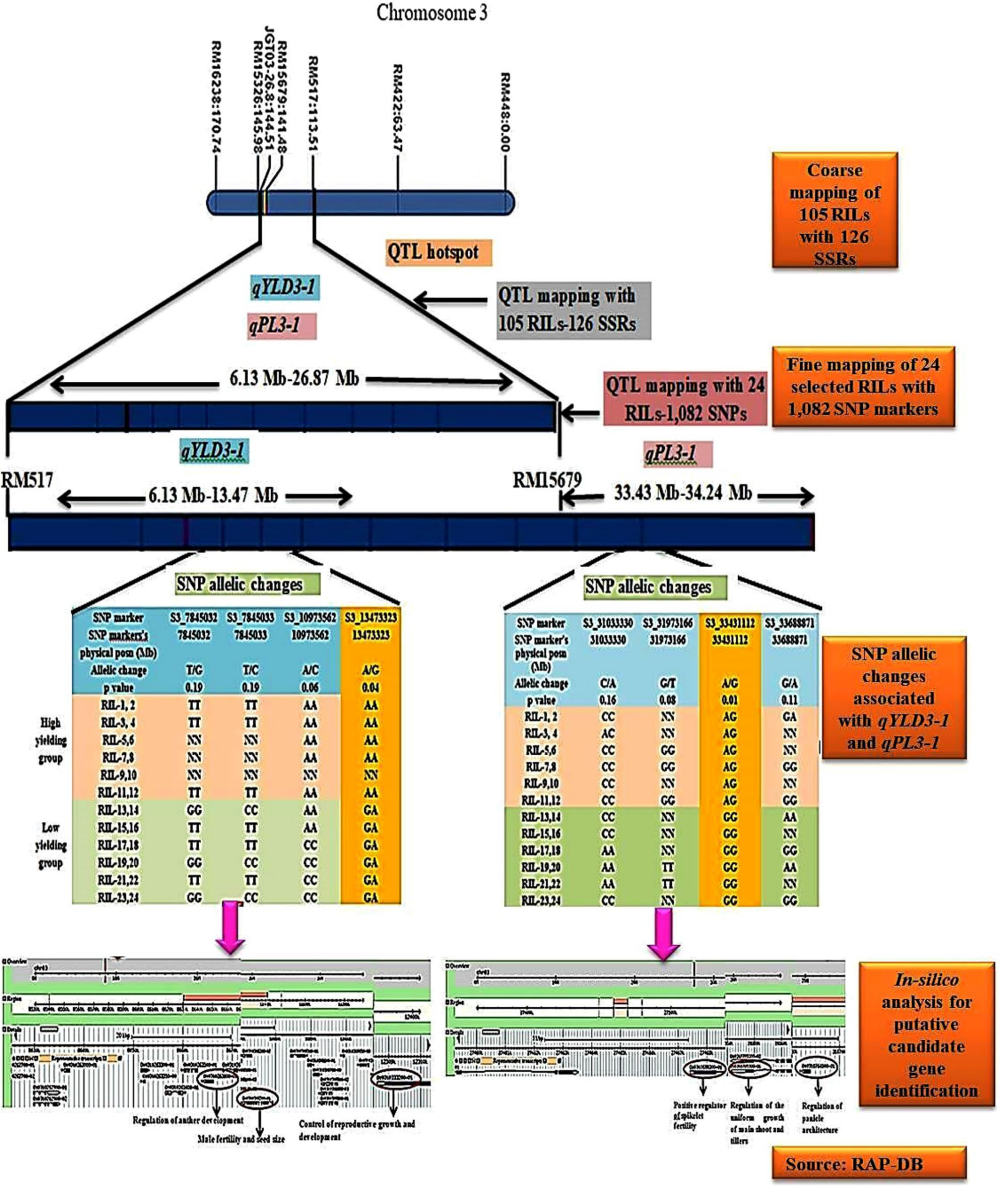
Scheme of fine mapping of YLD and PL QTLs on chromosome 3 with 24 selected RILs and 1,082 SNP markers. Significant SNP changes highlighted in yellow.

Supplementary Table 14 lists the putative candidate gene (s) identified within the regions of two commonly detected QTLs. The major *qYLD3-1* was observed to be flanked between SNP markers, S3_6134304 (6.13 Mb) and S3_13473282 (13.47 Mb). The closest and significantly associated SNP marker identified with *qYLD3-1* was S3_13473282 with lowest probability (p-value) of 0.01. Adenine (A) to Guanine (G) biallelic transitions among both the high and low yielding groups were observed with SNP marker, S3_13473282 (Fig. 3). Based on the physical positions of annotated genes between 6.13 and 13.47 Mb, a total of 1,228 genes were observed to be located in this interval. Out of them, three putative candidate genes, which are possibly associated with YLD trait, were identified (Fig. 3, Supplementary Table 14). The second QTL, *qPL3-1*, was observed to be flanked by S3_33431112 (33.43 Mb) and S3_34241104 (34.24 Mb). The closest associated SNP marker with this QTL was S3_33431112 with lowest probability (p-value) of 0.01. Adenine (A) to Guanine (G) biallelic transitions among both the high and low yielding groups (Fig. 3). Within the chromosomal position 33.43–34.19 Mb, a total of 162 annotated genes were observed in RAP-DB’s annotated data base. Among them, three putative candidate genes were identified whose biological functions were possibly associated with the trait, panicle length (Fig. 3, Supplementary Table 14).

Supplementary Table 15 details the list of putative candidate genes within the novel QTL regions identified using 105 RILs-126 SSR markers and 24 selected RILs-1,082 SNP markers.

### Percent of missing data in amplification with SSR and SNP markers

A total of 13,230 data-points were amplified with 126 SSR markers spread across all chromosomes in RIL population of 105 individuals. Out of them, 417 data-points were ambiguous and hence they were considered as missing. Therefore, percent of missing data-points with SSR markers was 3.15% [(417/13,230) × 100]. Concerning the SNP markers which were identified among the parents and the 24 selected RILs, a total of 25,968 filtered and non-redundant data-points were identified through low-throughput next-generation sequencing in Illumina platform and which were polymorphic between the parents. Among them, 2,166 data-points did not show any amplification. Therefore, percent of missing data with SNP markers was 8.34%.

### Presence of the major fertility restorer genes, *Rf3* and *Rf4* among the RIL population

The 12 RILs which were selected based on yield and plant height (i.e. 12 high yielding RILs; see Supplementary Table 1), were analyzed with the functional markers specific for *Rf3* (RMS-SF21-5) and *Rf4* (RMS-PPR9-1) genes. Among them, six RILs had alleles specific for both *Rf3*and *Rf4* genes and other six RILs had alleles specific only for *Rf3* gene. Test cross data (Supplementary Table 18) of high yielding RILs with IR58025A confirmed that all the RILs which were high yielding and possessed *Rf3* and *Rf4* alleles were complete restorers (viz., RIL-1, RIL-4, RIL-6, RIL-8, RIL-9, RIL-12) indicating that these lines may be useful in hybrid rice breeding. Selection efficiency of *Rf4* and *Rf3* markers, used in this study, in terms of major restorer lines identification was estimated to be 81.8% and 50%, respectively.

## Discussion

In the immediate future, diminishing natural resources coupled with burgeoning population would pose a severe food shortage in many rice growing countries across the world including India. This necessitates the scaling-up of rice production at least by 40%. Large-scale adoption of hybrid rice technology has the potential to enhance rice production and productivity. Till date, even though a total of 105 rice hybrids have been developed and commercially released for cultivation in India, hybrid rice occupies less than 3 million hectares out of 44 million hectares under rice cultivation^69^. The reasons for such a low-scale adoption of hybrid rice technology in India and concluded that the policy constraints, diminished facilitation for the commercialization of hybrid rice in the Indian market, lower magnitude of heterosis and technical challenges being the prime causes^70^. A modest yield advantage of 5–10% of rice hybrids over the best varieties has been identified as one of the primary reasons for its slow spread in the country^71,72^ opined that to make hybrid rice popular among the Indian farmers, the yield of the rice hybrids was expected to be at least 20–30% higher than the popular varieties. The projected increase in heterosis could be possible by resorting to the state-of-the art biotechnological tools along with the traditional breeding techniques^73^.

Agronomically important traits such as the yield are inherited quantitatively and follow a complex pattern of interaction. Such complex interactions and their mode of inheritance can be understood through the information on the quantitative trait loci (QTL) which influence the trait expression along with the cumulative contribution of these loci to the trait expression. Moreover, the epistatic or pleiotropic effects among the loci under various environmental variations are crucial for the employment of QTL for crop improvement^4^. Enhancement of heterosis levels in rice through development of potential hybrids depends on selection of diverse parents (i.e., the maintainers and restorers)^74^. One of the feasible options available for restorer diversification and selection is the development of segregating and immortal populations namely recombinant inbred lines (RIL), near isogenic lines (NIL), iso-cytoplasmic restorers (ICR) from popular hybrids^4,75^. Among them, development of RILs from elite, high yielding hybrids and identification of promising restorers would be profitable as these lines are iso-cytoplasmic to the hybrid and are therefore known to inherit full-complement of fertility restorer (*Rf*) genes for fertility restoration^75^. The present study was carried out with an objective to map novel genomic regions for the yield and its allied parameters in a set of recombinant inbred line (RIL) population derived from an elite hybrid KRH-2, with the help of SSR markers and their validation through SNP markers, aiming at restorer diversification.

While developing the RIL population derived from the hybrid, KRH-2, enough care has been taken to select seeds from each F_2_ plant and similarly the population was advanced to F_8_ generation. We observed that less than 3% of the population present in F_2_ generation was sterile (as presence of the major fertility restorer genes, *Rf3* and *Rf4* and few other minor loci associated with fertility restoration in the male parent, KMR3R (^101^Sheeba et al. (2009) and hence we could advance most of the F_2_ lines to F_8_ generation. The final set of RILs used in the present study showed nearly normal distribution for most of the agro-morphological characters studied (Supplementary Fig. 1). Additionally, the population used in this study was initially developed for two purposes viz., (1) development of iso-cytoplasmic restorer lines from the elite hybrid KRH-2, and (2) molecular mapping of yield and yield related traits in the hybrid KRH-2. Statistical analysis of RIL population for trait performance indicated that the choice of collection of the three seasons' mean data for QTL mapping (with a p value of ≤ 0.05) was appropriate for identification of major and minor effect QTLs. The W value was observed to approach a value of one showing the higher degree of uniformity among the plants of each. The p-value is usually analyzed with regard to the alpha (α) value which was 0.1 in this study. If the p-value was observed to be less than the alpha value (α), then the null hypothesis stating that the predictor has no effect on the outcome of the variable is rejected. In our study, as the p-value and Pr value for all the traits were observed to be less than 0.1, this demonstrated that all agro-morphological traits (called as sources or predictors, here) statistically affected the outcome of the QTL mapping with SSR markers. The skewness values for all the traits were in between the range − 1.96 and + 1.96, the distribution of the data is therefore observed to be univariate^76,77^. Kurtosis values for all the traits were observed to be less than three, indicating that the dataset is showing a normal distribution. Estimation of genetic and non-genetic factors in crop improvement is essential for efficient selective breeding and reliable estimate for increasing breeding value^78^. Broad sense heritability (*H*^2^) values for all the traits except for biomass (BM) indicated the possibility of improvement of most of the traits through selective breeding^79^.

Reference^4^ reported that one of the major drawbacks associated with QTL mapping experiments was the lack of consistency and reliability of identified QTLs when these experiments are repeated across environments with the same population. This limitation can be overcome by following stringent criteria as suggested by Ref.^80^ which include determination of an empirical significant threshold value for each experiment, considering the data from multiple replicates and/or across various environments with the same population and deployment of near-isogenic lines (NILs) for QTL confirmation. Such consistent and robust QTLs are quite important for envisaging future rice breeding programs. Taking these points into consideration, in the present study, we have collected data from multiple replicates and across different seasons in order to identify consistent QTLs. In our study, we observed a very low percentage (4.5%) of segregation distortion among the SSR markers. It might be as the RIL population was iso-cytoplasmic in nature (i.e. with WA-CMS conferring cytoplasm), some amount of segregation distortion is expected as only those breeding lines possessing one or more of the major or minor fertility restorer genes which will set seed in each generation of selfing. Population size of 105 RILs used in our study for QTL mapping of typical quantitative traits can be considered as optimum, even though not ideal, as previously reported by research groups^81–85^ successfully mapped yield related quantitative traits using a population size similar to the one used in our study. Limited number of 24 samples which consisted of 12 high and 12 low yielding RILs representing the two extreme groups of phenotypes was selected for SNP genotyping. Remaining RILs were placed within these groups. The results obtained through SNP based QTL mapping with a sub-set mapping population of 24 individuals and SSR based QTL mapping with all the 105 RILs were in congruence with each other for few of the major effect QTLs discovered. Two major effect QTLs namely *qYLD3-1* and *qPL3-1* were observed to be consistent when mapped with SSR and SNP markers. This is possibly due to two reasons (i) lesser number of SSR markers deployed for mapping the entire population as most of the SSR markers screened for parental polymorphism testing were monomorphic and (ii) smaller size of sub-set mapping population used for SNP-based QTL mapping i.e., only 24 selected RILs was SNP genotyped due to limited resources. Even though the sub-set population size was a limited; we were able to map the major effect QTLs with better resolution using SNP markers to find the causative SNP variations.

In order to identify the genomic regions that govern yield and its related traits across various population sets in rice, the QTLs identified in this study were compared with other earlier studies. It was observed that out of the 22 identified QTLs with SSR markers, 19 were reported and mapped previously in the same genomic regions indicating the consistency of these QTLs across various populations of *Oryza* sp. The remaining three QTLs, namely, *qPL3-1*, *qPW6-1*and *qPW8-1*, are indeed novel.

At least two QTLs were detected for each trait, except for flag leaf length (FLL) and number of productive tillers (PT). The phenotypic variation (PVE%) of all identified QTLs was in the range of 5.08% (for trait biomass (BM)) to 22.76% (for trait total grain yield/plant (YLD)) at 0.05 level of significance indicating the reliability of the identified QTLs. Also, the LOD peak values for the identified QTLs were in the range of 2.53 (for trait panicle length (PL) to 10.59 (for trait grain yield/plant (YLD). Majority (54.45%) of the identified QTLs were observed to increase the trait value due to the presence of favorable allele from KMR-3R as compared to the other parent, IR58025A. This observation demonstrates the importance of existence of significant variation among the two parental lines and parental allelic distribution for reliable QTL mapping^4^. A total of 12 major and minor effect QTLs, which were identified for traits such as total grain yield/plant, panicle weight, plant height, panicle length, days to fifty percent flowering, total number of grains per panicle, fertile grains per panicle, test grain weight and biomass, were contributed by KMR-3R. Similarly, a total of 10 major and minor effect QTLs for traits namely flag leaf width, days to fifty percent flowering, total grain yield/plant, test grain weight, panicle weight, plant height and biomass were contributed by IR58025A. In some of the high-yielding RILs (RIL-1, RIL-4, RIL-6, RIL-8, RIL-9, RIL-12), it was observed that there were many favorable QTLs from KMR-3R (*qYLD3-1, qPL3-1, qPW3-1, qFLW4-1, qPH12-1, qDFF9-1, qGP8-1, qGP12-1, qFGP8-1, qTGW5-1, qTGW12-1, qPW8-1, qPH12-2, qPL9-1, qBM11-1*) and a few from IR58025A (*qDFF12-1, qYLD6-1, qTGW8-1, qPW6-1, qFLW4-2, qPL6-1, qBM5-1*), indicating complementary action of alleles from both the parents.

Co-localization of many major effect QTLs with previously reported QTLs was observed in this study. For e.g., *qYLD3-1* was identified between 6.13–26.87 Mb, flanked by RM517-RM15679 and co-localized with grain weight/plant (Y) QTL reported by Ref.^86^. *qYLD3-1*, identified in this study, was also reported earlier by Ref.^82^ with the name GYPa1 (grain yield per plant) when QTL mapping was done with BC_2_F_2_ population derived from the cross between a highly inbred population of wild rice *Oryza glumaepatula*, RS-16 and high yielding elite inbred line *Oryza sativa*, BG90-2. Marker intervals of major effect QTL for panicle length (PL) trait, *qPL3-1*, did not overlap with any of the earlier reported QTLs and was considered as novel. Major effect QTL for panicle weight (PW) trait, *qPW3-1*, (26.8 Mb) identified in this study was reported earlier by Ref.^87^ with QTL named qGWP-3a (for trait weight of grain panicle). We identified the major effect QTL, *qFLW4-1*, for the trait, flag leaf width (FLW), flanked by the markers, RM6909-RM252 (32.0–24.02 Mb), co-localized with QTL, QFlw4 reported by Ref.^88^ flanked by the markers RM255-RM349 (30.9–32.7 Mb). *qPH12-1*(0.2–19.4 Mb), the major effect QTL for plant height (PH) trait discovered in our study was co-localized with an earlier reported QTL^89^. Among the minor effect QTLs, *qPW6-1* and *qPW8-1*, did not co-localize with earlier studies and were identified to be novel.

Nearing zero values of the parameters that effect the QTL × E interaction namely the LOD (AbyE), PVE (AbyE) and AbyE in both major and minor effect QTLs demonstrated no significant effect of the environment on the expression of QTLs across three consecutive seasons at one location. Non-significant differences among the RSq values of the QTLs also demonstrate a similar observation. The environmental variation was very limited as 3 environments under this study constituted in the same location and any effect was only due to seasonal differences.

Some interesting observations were made in the present study of QTL mapping. It was observed that 50% of the QTLs identified with SNPs had a better resolution than those identified with SSRs. Two QTLs namely *qYLD3-1* and *qPL3-1* which were detected commonly with both the types of markers were observed to have an improved resolution, when QTL mapping was done with 24 selected RILs-1,082 SNPs. Also, the percentage of major effect QTLs identified with SNP markers was 44% which was 26% more than the major effect QTLs detected with SSRs. Moreover, 59% of the QTLs showed a better resolution than SSR mapped QTLs and the sizes of SNP mapped QTLs was observed to be in the range 0.03–5.78 Mb. Further, an increment of 9% in the novel QTL detection with 1,082 SNPs was observed vis-à-vis SSRs. A total of eight novel QTLs with SNPs were detected for the following traits: total grain yield/plant, days to fifty percent flowering, panicle weight, panicle length, flag leaf length whose resolution was in the range 0.03–2.87 Mb. This could be due to deployment of a sub-set mapping population from the RILs (i.e. 12 high-yielding and 12 low-yielding) and also because of deployment of larger number markers in SNP mapping as compared to mapping using SSR markers.

In QTL mapping with 1,082 SNPs, a total of five major effect QTLs were observed to co-localize with earlier studies, the details of which are present herewith. In this study, *qTGW11-1* (1.12–0.96 Mb), a major effect QTL identified overlapped with an earlier reported by Ref.^90^ (0.47–2.84 Mb). *qPW2-1,* panicle weight QTL (5.35–8.22 Mb) identified in this study was observed to localize very closely with the *qGWP-2* (weight of grain panicle QTL) (4.41–5.20 Mb) reported by Ref.^87^. As *qPW2-1* did not overlap with *qGWP-2* hence *qPW2-1* was identified to be a novel QTL. Whereas the panicle length QTL (5.35–8.22 Mb) *qPL2-1*, was observed to overlap with *qPL2-1* (4.40–11.38 Mb) reported by^4^ though the resolution of QTL in our study was better with the help of 1,082 SNP markers. The QTL for plant height trait (5.35–8.22 Mb) *qPH2-1* co-localized with QTL *ph2.1* (4.63–35.77 Mb) reported by Ref.^91^ and the resolution of *qPH2-1* was better when QTL mapped with 1,082 SNP markers. Lastly, *qFGP5-1* (29.80–19.21 Mb) and *qFGP5-2* (3.36–29.95 Mb) were observed to co-localize with qFG5-1, QTL for filled grains per panicle (FG) reported by Ref.^83^. Co-localization of minor effect QTLs identified with 24 selected RILs and 1,082 SNPs is presented in Supplementary Table 11.

### SSR and SNP based QTL hotspots

QTL hotspots, the genomic region of a chromosome harboring QTLs associated with multiple traits, were identified with the major and minor effect SSR and SNP based QTLs. A region on chromosome 3, sized 27.97 cM and flanked by SSR markers, RM517 at 113.51 cM and right SSR RM15679 at 141.48 cM was identified to control two traits namely, total grain yield/plant (YLD) and panicle length (PL). This QTL hotspot region was earlier reported by Ref.^4^ as they identified a 50.2 cM chromosomal region flanked by markers, RM7 (92.9 cM) and GNMS1140 (143.1 cM) and was known to govern two traits namely, panicles per plant (*qPPP3-1* located at 142.9 cM) and panicle weight (*qPL3-3* located at 112.9 cM).

Among the minor effect QTLs, three QTL hotspots were identified on chromosomes 5, 6 and 8 as shown in Supplementary Table 7. A 27.97 cM sized QTL region on chromosome 5, flanked by RM18414 and RM18516 was observed to control the expression of two traits namely, test (1,000) grain weight (TGW) and biomass (BM). This QTL hotspot was reported earlier by Ref.^92^ flanking between marker intervals wd5002636 (2.5 cM)-id5001470 (19.5 cM) which harbored QTLs for early vigor, early uniform emergence, shoot length, shoot fresh weight, shoot dry weight, and total dry weight. A second region identified on chromosome 6 in between the regions flanked by RM7023 and ESSR06-7.1 was observed to govern three traits namely, total grain yield/plant (YLD), panicle weight (PW) and panicle length (PL) and was identified to be novel. Another novel QTL hotspot region on chromosome 8 was identified in between the flanking markers, RM1384 (140 cM) and SSR-8–13 (153.39 cM) which was observed to control the expression of two traits namely, total grains/panicle (GP) and panicle weight (PW).

Similarly, QTL hotspots were also identified in the mapping using SNP markers. A QTL hotspot of 2.87 Mb size on chromosome 2 flanked between SNP markers, S2_5359418 (5.35 Mb) and S2_8229921 (8.22 Mb) controlling four traits namely total grain yield/plant (*qYLD2-1*), panicle weight (*qPW2-1*), panicle length (*qPL2-1*) and plant height (*qPH2-1*) was identified. Various groups identified the QTLs for the above-mentioned traits and the QTL regions reported in those studies overlapped with the ones those reported here. For example, Ref.^87^ identified a QTL region for panicle weight trait on chromosome 2 between 4.41 and 5.20 Mb and which was in the vicinity of *qPW2-1* identified in this study. Reference^4^ identified a QTL on chromosome 2 for panicle length trait between 4.40 and 11.38 Mb and *qPL2-1* (5.35–8.22 Mb), however, QTL identified in this study was observed to have a better resolution. *qPH2-1* identified with better resolution overlapped with the QTL for plant height reported by Ref.^91^ which was 31.14 Mb in size. *qYLD2-1* is identified as a novel QTL. Second QTL hotspot region was identified on chromosome 4 between the region, 7.95 Mb (flanked by S4_7956048 SNP marker)-6.25 Mb (flanked by S4_6251080 SNP marker), which controlled two traits namely, total grain yield per plant and days to fifty percent flowering. Though *qDFF4-1* was reported to be novel, *qYLD4-1* was resolved to 6.54 Mb by Ref.^81^. Third QTL hotspot identified between 20.69 and 9.62 Mb region of chromosome 10 was observed to control two traits namely panicle weight (*qPW10-1*) and panicle length (*qPL10-1*). This hotspot is reported to be novel. No QTL hotspot regions were identified in common with SSR and SNP markers.

Based on the lowest p-value and QTL table in QTARO database, an account of involvement of identified SNP loci associated with major effect QTLs in regulating different traits other than the primary trait with which it is associated was analyzed. Three major effect QTLs namely, *qYLD3-1* (for trait total grain yield/plant), *qPW3-1* (for trait panicle weight), *qPL3-1* (for trait panicle length) had common SNP identified at 27.51 Mb. From QTARO database, it was known that this SNP was a part of an earlier reported QTL, QFlw3 (27.49–31.49 Mb)^93^, which had a role in diverse functions such as grains per panicle and carbon isotope discrimination (under drought tolerance) and chlorophyll content. The appropriateness of SNPS3_27516811 associating with total grain yield/plant in our study was realized from the fact that the earlier reported QTL, QFlw3, influenced yield/plant to a major extent. For the major effect QTL, *qFLW4-1* (for trait flag leaf width), a strongly associated SNP was identified at 24 Mb (SNP marker-S4_24002530). Genomic region between 19.92 and 29.15 Mb of chromosome 4 which harbored the identified SNPalong with earlier reported gene (s)/QTL(s)^94^ was associated with following traits: leaf chlorophyll content at flowering (SPAD)(controlled by the QTL, *qCCFJ-4*), boron soil tolerance (soil stress tolerance, S-9 gene), d11 gene associated with dwarfism, *qPH1-4-1*-QTL associated with plant height at 35 DAT, QTLs *qChla4-1* and *qChlab4-1* were observed to regulate the content of chlorophyll a and total chlorophyll content, respectively, in shoots-seedlings. It was interesting to note that SNP, S4_24002530, co-localized with an earlier reported gene *or11* governing leaf character. The SNP associated with *qPH12-1* (SNP marker-S12_14867709), a major effect QTL for plant height was identified at 14.86 Mb. An earlier reported QTL, *qtl12.1* (9.89–17.75 Mb)^89^ co-localizing with the identified SNP, was known to play a significant role in drought tolerance and was reported to control the following traits: harvest index, panicle number, drought response index, grain yield, biomass yield. SNP, S12_14867709, was observed to co-localize with a QTL for height, *qtl12.1*, and this corroborated our observations.

### In-silico identification of putative candidate gene (s) underlying the major QTLs

In-silico analysis and putative candidate gene (s) identification for the commonly identified QTLs namely *qYLD3-1* and *qPL3-1* with SSR and SNP markers revealed three putative candidate genes which possibly may be associated with yield related traits. Pertaining to the *qYLD3-1*, the first putative gene, *Os03t0263600-01*, identified at 8.66 Mb was atypical strictosidine synthase protein that played an important role in regulation of anther development and pollen wall formation. The second putative candidate gene, *Os03t0308200-01*, observed at 10.97 Mb was a predicted protein that regulated the male fertility and seed size. The third putative candidate gene, *Os03t0333200-01* identified between 12.30 and 12.31 Mb was a receptor like kinase that controlled the reproductive growth and development of the rice plant. Putative candidate genes which may be closely associated with *qPL3-1* include the first gene; *Os03t0809900-01* at 33.89 Mb was core protein subunit of exon junction complex (EJC) that regulated the embryonic organogenesis and development. The second gene, *Os03t0815700-01* at 34.19 Mb was identified to be a K Homology domain containing protein, nuclear RNA/DNA binding protein of the STAR (Signal Transduction and Activation of RNA) family that regulated flowering time control. Though slightly upstream of the physical position of *qPL3-1*, third candidate gene, *Os03t0764900-01* (31.66 Mb), a DOF transcription factor, was identified to play a very crucial role by directly regulating the panicle architecture. Therefore, the candidate gene (s) associated with each of the QTL were identified not only within the significantly associated SNP marker region but also within the vicinity of the SNP markers physical chromosomal position.

### Genetic architecture of novel YLD related QTLs

Genetic architecture of rice yield is considerably influenced by the number of productive tillers (PT) and panicle morphology. The morphology of panicle encompasses important aspects such as panicle length (PL) and panicle weight (PW). Though days to fifty percent flowering (DFF) is usually observed to be negatively correlated with grain yield (YLD), its contribution in genetic architecture of YLD cannot be over-ruled. In our study, putative candidate gene (s) associated with above mentioned traits and those which may influence the genetic architecture of total grain yield per plant (YLD) were identified and is presented. The details of putative candidate genes which were identified in common to the QTLs identified with 105 RILs-126 SSRs and 24 selected RILs-1,082 SNPs is presented in the above section. Novel QTL *qPW6-1*, which was identified with 105 RILs-126 SSRs dataset, had three putative candidate genes. *Os06t0234100-01* (6.90–6.98 Mb), a peptidase S1C, HrtA/DegP2/Q/S family protein. *Os06t0234150-00* (6.90–6.98 Mb), a non-protein coding transcript; *Os06t0234100-02* (6.90–6.98 Mb) was observed to be similar to DEGP9 (DEGP PROTEASE 9); serine-type peptidase/ trypsin. Though a definite role of these candidate genes is yet to be identified, they may have a biological function in influencing the PW trait. The biological functions of putative candidate genes in novel QTLs identified in our study with 24 selected RILs-1,082 SNPs is described. *qDFF2-1* was identified with two putative candidate genes, *Os02t0150800-01* and *Os02t0152500-01*. *Os02t0150800-01* (2.79–2.80 Mb), F-box protein with a LOV domain and consecutive Kelch repeats was identified to influence circadian clock associated-component and *Os02t0152500-01* (2.87–2.88 Mb)(chromatin remodeling factor) was identified to have a role in positive regulator of flowering. Putative candidate gene, *Os04t0213100-01* (7.55 Mb) associated with *qDFF4-1* is a hemopexin fold protein regulating anther development. *Os10t0478000-01* (17.88 Mb), a putative candidate gene associated with *qPW10-1* is a transcriptional regulator and has a role in the regulation of inflorescence development. *qPL10-1*, had three putative candidate genes. *Os10t0478200-01* (17.91 Mb) is a NAD-dependent cytosolic malate dehydrogenase (CMDH) regulating the starch synthesis and seed development. The second putative candidate gene, *Os10t0498600-01* (19–19.01 Mb) is a member of pre-mRNA processing (Prp1) family that regulates the starch biosynthesis. The third putative candidate gene, *Os10t0508100-01* (19.46 Mb) is a DUF641 domain containing protein regulating the rate of grain-filling. The first putative candidate gene, *Os03t0809900-01* (33.89 Mb) identified with *qPL3-1*, is a core subunit of exon junction complex (EJC) and regulates embryonic organogenesis and development. *Os03t0815700-01* (34.19 Mb) is a K Homology domain containing protein controlling the flowering time. Therefore, as biological functions of these putative candidate genes are relatable to the novel QTLs identified for their respective traits, it is speculated that they may have a role in the expression of the trait (s) and hence influencing the genetic architecture of YLD trait.

As the QTL mapping results obtained from analysis in the sub-set mapping population (consisting of 24 selected individuals) and the larger RIL population were similar in this study, it will be desirable in future to analyze a small sub-set population (consisting of phenotype extremes) with high throughput markers (like SNPs), rather than analysis with larger sized populations with low throughput markers like SSRs. This is further exemplified by the fact that the resolution of the major effect QTLs was also better in mapping analysis using SNP markers, even though the population consisted of only 24 individuals. This observation was further corroborated with the correlation study among the agro-morphological traits in sub-set population. This also demonstrated the feasibility of SNP allelic change identification among the major effect QTLs.

### Identification of efficient restorers among the RIL population

Over the years, commercial production of hybrid rice in most of the Asian countries including India is extensively based on wild-abortive cytoplasmic male sterility system, which is popularly known as WA-CMS system^95,96^ or three-line system. Many tropical rice growing countries including India have prioritized their hybrid rice production in order to meet the challenge of feeding its growing population^66^. As rice hybrids have recorded a yield advantage of 15–20% over the most popular commercial inbred lines^97^, adoption of the hybrid rice technology is one of the realistic options in order to achieve national food security in India. Though large-scale adoption of hybrid rice in India remains a challenge^8^, nevertheless, using the WA-CMS system, at least 106 hybrids have been developed, released and recommended for commercial cultivation^69^. Identification and characterization of restorer lines for the presence of major fertility restoration genes/loci (for their effective fertility restoration capability) is crucial. Among the 17 fertility restoration loci identified so far, presence of two dominant genes, *Rf3* (located on chromosome 1) and *Rf4* (located on chromosome 10), are known to regulate fertility restoration of WA cytoplasm^98,99^ to a significant extent and hence are considered as major genes controlling the trait. To a larger extent, the choice of CMS lines to be used in hybrid rice breeding program is dependent on the availability of appropriate restorer lines. Improvement in the yields of the hybrid seeds due to the deployment of taller restorers is well-known. These restorers which are comparatively taller than the CMS lines ensure adequate pollination and such restorers are crucial for hybrid rice improvement^75^. As pointed out by Ref.^87^, one of the important parameters for yield determination in rice was the plant height which was evident from the fact that the yield of semi-tall plants was better than the dwarf plants. The traditional method of restorer line characterization involved tedious test-crosses with CMS lines and agro-morphological evaluation of derived F_1_ hybrids for spikelet and pollen fertility. The time and effort in restorer characterization can be drastically reduced with molecular mapping of the fertility restoration genes^100,101^. Moreover, molecular markers specific for *Rf3* and *Rf4* genes can facilitate introgression of these genes into elite backgrounds along with identification of genetic impurities in hybrid seed lots^102,103^. Contribution of wild abortive (WA)-type CMS-based hybrids to the global rice cultivation is significant. Therefore, an extensive investigation was carried out on major loci, namely, the *Rf3* and *Rf4* which govern the fertility restoration trait^104,105^. Identification of complete/partial restorers from promising breeding lines with unknown restoration ability can be known with molecular markers with 80–85% efficiency without the need of tedious test-crosses^106^.

The selection efficiency of *Rf4* and *Rf3* markers, used in this study, was estimated to be 81.8% and 50%, respectively, these observations imply that a complete restorer would essentially possess *Rf4* gene in combination with *Rf3* gene or *Rf4* alone and also, presence of *Rf3* gene alone might be a partial restorer. These findings were supported by earlier groups who concluded the fertility restoration trait was controlled largely by *Rf4* gene and not by *Rf3*. Agro-morphological evaluation of F_1_s derived from the crosses between high-yielding and semi-tall to tall RILs with IR58025A, suggests that RIL-1, RIL-4, RIL-6, RIL-8, RIL-9, RIL-12 were complete restorers and were having both *Rf3* and *Rf4* genes. Our observations are in accordance with those of Refs.^66,101^ and this was also validated through test crossing. Moreover, our results also demonstrate a large variation in the pollen fertility among the selected high-yielding RILs. This indicates that during the course of development of RIL population, the probable genetic interaction of fertility restorer genes with modifiers^107–110^ may have resulted in different genomic backgrounds of RILs (with respect to fertility restoration). Such interactions would have resulted in the development of RILs which may not inherit the complete complement of fertility restorer genes and thus may remain as partial restorers (RIL-2, RIL-3, RIL-5, RIL-7, RIL-10, and RIL-11) as observed in our study. Though the selection efficiency of markers specific for *Rf4* and *Rf3* loci in 12 high-yielding RILs was 81.80% and 50%, respectively, the precision with which these functional markers identified complete and partial restorers among the high yielding RILs was further corroborated with test-cross data. In our study, this observation indicates no inter-dependence of selection efficiency of markers on the number of samples analyzed. Through our earlier studies, Sheeba et al.^101^, Pranathi et al.^66^ we have already validated the selection efficiency of these markers.

## Conclusion

This study identified several major and minor effect QTLs from both male and female parents of KRH-2, which co-localized with QTLs, discovered in earlier studies, indicate that the genomic regions underlying the QTLs could be possible hotspots for yield heterosis associated traits. Further, a novel major effect robust QTL for panicle length trait along with two novel minor effect QTLs with conspicuous cumulative phenotypic variance have also been identified using SSR markers. Four novel major effect QTLs for total grain yield per plant, panicle weight, panicle length and flag leaf length were identified with SNP markers. The SNP allelic changes associated with each of the novel and reported QTLs provided an insight into the genes underlying the agro-morphological traits. Through marker assisted backcross breeding, novel QTLs and QTL hotspots could be introgressed into different genetic backgrounds so as to overcome yield barriers, ensure food security and a deeper understanding on the complex genetic interactions among the QTLs for trait enhancement. Through transformation studies in rice, a confirmation on role of identified putative candidate gene (s) with the associated agro-morphological trait could be taken up. It is expected that high yielding rice hybrids could be developed in the future using the selected RILs harboring the major QTLs.

## Supplementary information

Supplementary file 1

## Abbreviations

QTL: Quantitative trait loci
RIL: Recombinant inbred line
SSR: Simple sequence repeats
ICIM: Inclusive composite interval mapping
SNP: Single nucleotide polymorphism
GWAS: Genome wide association mapping
GBS: Genotyping-by-sequencing
KRH-2: Karnataka rice hybrid-2
RARS: Regional Agricultural Research Station
KMR-3R: Karnataka Mandya rice-3R
SSD: Single seed descent
DFF: Days to fifty percent flowering (DFF)
YLD: Total grain yield per plant
GP: Total number of grains per panicle
FGP: Fertile grains per panicle
TGW: Test (thousand) grain weight
PW: Panicle weight
PH: Plant height
PL: Panicle length
FLL: Flag leaf length
FLW: Flag leaf width
PT: Productive tillers
BM: Biomass
CV%: Coefficient of variation in percentage
STAR: Statistical Tool for Agricultural Research (STAR) software version 2.0.1
ANOVA: Analysis of variance
DNA: Deoxy ribose nucleic acid
CTAB: Cetyl trimethyl ammonium bromide
PCR: Polymerase chain reaction
TrisHCl: Tris aminomethane hydrochloric acid
KCl: Potassium chloride
mM: Milli molar
MgCl_2_: Magnesium chloride
dNTP: Deoxyribonucleotide triphosphate
pmol: Pico mole
pH: Potential of hydrogen
ng: Nano gram
U: Unit
v/v: Volume/volume
°C: Degree centigrade
EST: Expressed sequence tags
LOD: Log-likelihood ratio
PVE: Phenotypic variation explained
cM: Centi Morgan
SAM file: Sequence alignment map file
MAF: Minor allele frequency
GLM method: General linear model method
WA-CMS line: Wild abortive-cytoplasmic male sterile line
