# Supplementary material for: Molecular mapping of QTLs for yield related traits in recombinant inbred line (RIL) population derived from the popular rice hybrid KRH-2 and their validation through SNP genotyping
Source: Sci Rep. 2020 Aug 13;10:13695. doi: 10.1038/s41598-020-70637-3 (PMC7427098; doi:10.1038/s41598-020-70637-3)
Supplement: Supplementary file 1 [file 41598_2020_70637_MOESM1_ESM.docx]

**Molecular mapping of QTLs for yield related traits in recombinant inbred line (RIL) population derived from the popular rice hybrid KRH-2 and their validation through SNP genotyping**

Swapnil Ravindra Kulkarni^1*^, Balachandran SM^1*#^, Ulaganathan K^2^, Divya Balakrishnan^1^, Praveen M^1^, Hari Prasad AS^1^, Fiyaz RA^1^, Senguttuvel P^1^, Pragya Sinha^1^, Ravindra R Kale^1^, Rekha G^1^, Kousik MBVN^1^, Harika G^1^, Anila M^1^, Punniakoti E^1^, Dilip T^1^, Hajira SK^1^, Pranathi K^1^, Ayyappa Das M^1^, Mastanbee Shaik^1^, Chaitra K^1^, Koteswara Rao P^1^, Sunil S Gangurde^3^, Manish K Pandey^3^ and Sundaram RM^1*#^

^1^ICAR-Indian Institute of Rice Research (ICAR-IIRR), Hyderabad, India

^2^Centre for Plant Molecular Biology (CPMB), Osmania University, Hyderabad, India

^3^International Crops Research Institute for the Semi-Arid Tropics (ICRISAT), Hyderabad, India

**# Corresponding authors:**

Dr. RM Sundaram, Principal Scientist (Biotechnology), Crop Improvement Section, ICAR-Indian Institute of Rice Research, Rajendranagar, Hyderabad-500030, India

Email ID: rms_28@rediff.com, Phone (Off): 91-040-24591226; Fax: 91-040-24591217

Dr. SM Balachandran, Principal Scientist (Biotechnology) & Head, Crop Improvement Section, ICAR-Indian Institute of Rice Research, Rajendranagar, Hyderabad-500030, India. Email ID: balasena@yahoo.com, Phone (Off): 91-040-24591228; Fax: 91-040-24591217

* These authors contributed equally

**Acknowledgement**

Swapnil is grateful to DST INSPIRE Fellowship Division, New Delhi for providing the financial assistance (Grant # DST/INSPIRE Fellowship/2013/1146) and to the Director, ICAR-IIRR for providing the infrastructural facilities for carrying out this research work. Praveen is thankful to Department of Science and Technology, Government of India for providing funding support through the SERB-NPDF Scheme (Grant: PDF/2016/000374).

**Supplementary Table 1:** Three seasons’ mean agro-morphological data of 105 recombinant inbred lines (RILs) derived from the cross of IR58025A× KMR-3R

| Material | *Rf3/Rf4* status | DFF±SE | YLD±SE | GP±SE | FGP±SE | TGW±SE | PW±SE | PH±SE | FLL±SE | FLW±SE | PT±SE | PL±SE | BM±SE |
| --- | --- | --- | --- | --- | --- | --- | --- | --- | --- | --- | --- | --- | --- |
| IR58025B (Parent 1) | *rf3rf3/ rf4rf4* | 102±0.23 | 18.7±0.11 | 289±0.96 | 251±0.15 | 14.6±0.98 | 23.69±0.43 | 120.4±0.98 | 27.2±0.55 | 1.32±0.12 | 14.4±0.36 | 22.8±0.96 | 47.98±0.13 |
| KMR-3R (Parent 2) | *Rf3Rf3/Rf4Rf4* | 110±0.15 | 24.2±0.74 | 320±0.34 | 306±0.33 | 18±0.77 | 27.56±0.12 | 101.8±0.13 | 32.5±0.45 | 1.46±0.33 | 16.8±0.37 | 24.8±0.56 | 52.36±0.11 |
| KRH-2 (Hybrid) | *Rf3Rf3/Rf4Rf4* | 106±0.36 | 27.33±0.63 | 348±0.44 | 312±0.13 | 22±0.36 | 29.33±0.41 | 93.00±0.89 | 33.00±0.33 | 1.2±0.48 | 20.3±0.75 | 26.4±0.45 | 54.99±0.99 |
| Akshayadhan (Check) | *Rf3Rf3/Rf4Rf4* | 129±0.12 | 20.83±0.42 | 335±0.30 | 309±0.97 | 20±0.15 | 29.76±0.36 | 119.2±0.53 | 33±0.36 | 1.42±0.77 | 16.6±0.25 | 26.1±0.12 | 51.23±0.45 |
| Varadhan (Check) | *Rf3Rf3/Rf4Rf4* | 112±0.15 | 22.03±0.13 | 327±0.12 | 311±0.12 | 22.03±0.11 | 28.13±0.45 | 114±0.12 | 32.2±0.12 | 1.62±0.99 | 19±0.45 | 24±0.11 | 53.69±0.23 |
| RIL-1 | *Rf3Rf3/Rf4Rf4* | 102±0.32 | 36.48±0.36 | 356±0.12 | 320±0.11 | 22.2±0.12 | 29.48±0.56 | 110±0.11 | 30.66±0.22 | 1.52±0.11 | 17±0.23 | 23.86±0.23 | 23.24±0.45 |
| RIL-2 | *Rf3Rf3/ rf4rf4* | 98±0.12 | 32.62±0.45 | 369±0.66 | 316±0.33 | 23±0.33 | 25.62±0.23 | 110±0.68 | 30.66±0.12 | 1.63±0.42 | 18±0.11 | 25.03±0.12 | 22.00±0.21 |
| RIL-3 | *Rf3Rf3/ rf4rf4* | 98±0.45 | 30.12±0.12 | 345±0.47 | 323±0.69 | 22±0.98 | 23.12±0.65 | 121±0.14 | 30.96±0.36 | 1.62±0.33 | 20±0.93 | 23.33±0.33 | 31.96±0.37 |
| RIL-4 | *Rf3Rf3/Rf4Rf4* | 99±0.33 | 29.4±0.33 | 344±0.33 | 322±0.75 | 20±0.36 | 22.4±0.45 | 124±0.78 | 31.86±0.15 | 1.56±0.14 | 19±0.12 | 24.75±0.45 | 37.00±0.45 |
| RIL-5 | *Rf3Rf3/ rf4rf4* | 106±0.26 | 28.92±0.31 | 355±0.44 | 320±0.22 | 24±0.45 | 18.99±0.23 | 117±0.15 | 29.66±0.97 | 1.6±0.66 | 19±0.32 | 23.9±0.36 | 28.87±0.12 |
| RIL-6 | *Rf3Rf3/Rf4Rf4* | 98±0.18 | 28.36±0.12 | 345±0.12 | 322±0.14 | 22±0.66 | 21.36±0.33 | 119±0.23 | 31.33±0.65 | 1.58±0.44 | 22±0.33 | 25±0.65 | 33.88±0.35 |
| RIL-7 | *Rf3Rf3/ rf4rf4* | 102±0.89 | 28.28±0.39 | 399±0.36 | 356±0.36 | 20±0.78 | 15.63±0.45 | 116±0.89 | 32.6±0.32 | 1.73±0.33 | 15±0.12 | 23.9±0.47 | 26.48±0.11 |
| RIL-8 | *Rf3Rf3/Rf4Rf4* | 102±0.11 | 27.9±0.12 | 347±0.11 | 320±0.89 | 21±0.37 | 18.77±0.33 | 121±0.75 | 30.73±0.36 | 1.66±0.47 | 16±0.78 | 24.6±0.37 | 36.66±0.65 |
| RIL-9 | *Rf3Rf3/Rf4Rf4* | 107±0.88 | 27.86±0.49 | 345±0.36 | 320±0.78 | 21±0.18 | 20.86±0.11 | 116±0.45 | 30.16±0.58 | 1.56±0.83 | 16±0.33 | 24.73±0.89 | 19.9±0.98 |
| RIL-10 | *Rf3Rf3/ rf4rf4* | 99±0.78 | 27.8±0.82 | 369±0.15 | 345±0.33 | 23±0.96 | 20.8±0.32 | 118±0.11 | 29.16±0.69 | 1.45±0.12 | 22±0.64 | 24.06±0.45 | 49.4±0.99 |
| RIL-11 | *Rf3Rf3/ rf4rf4* | 101±0.77 | 27.88±0.70 | 345±0.37 | 322±0.12 | 16±0.45 | 20.88±1.22 | 117±0.36 | 28.87±0.45 | 1.71±0.36 | 15±0.79 | 26.47±0.69 | 38.82±0.78 |
| RIL-12 | *Rf3Rf3/Rf4Rf4* | 93±0.88 | 23.22±0.39 | 355±0.96 | 321±0.37 | 17±0.33 | 16.22±0.36 | 114±0.15 | 22±1.22 | 1.3±0.22 | 15±0.84 | 22.3±0.45 | 24.86±0.66 |
| RIL-13 | *Rf3Rf3/Rf4Rf4* | 108±0.44 | 13.26±0.33 | 356±0.45 | 304±0.11 | 19±0.12 | 16.26±0.32 | 107±0.35 | 24.9±0.36 | 1.81±0.45 | 22±0.31 | 25.9±0.12 | 48.4±0.45 |
| RIL-14 | *Rf3Rf3/Rf4Rf4* | 103±0.78 | 13.1±0.12 | 367±0.33 | 322±0.39 | 20±0.36 | 16.1±0.16 | 99±0.43 | 25.85±0.66 | 1.39±0.12 | 23±0.34 | 23.53±0.56 | 25.66±0.03 |
| RIL-15 | *Rf3Rf3/ rf4rf4* | 118±0.77 | 13.04±0.96 | 356±0.11 | 321±0.45 | 19±0.45 | 16.04±0.33 | 98±0.11 | 30.73±0.45 | 1.24±0.33 | 24±0.85 | 25.13±0.36 | 27.84±0.12 |
| RIL-16 | *Rf3Rf3/ rf4rf4* | 111±0.68 | 12.64±0.45 | 355±0.36 | 322±0.62 | 19±0.78 | 15.64±0.96 | 99±0.99 | 35.46±0.67 | 1.41±0.21 | 21±0.69 | 25.23±0.45 | 36.98±0.32 |
| RIL-17 | *Rf3Rf3/ rf4rf4* | 102±0.31 | 12.56±0.33 | 378±0.42 | 326±0.78 | 18±0.63 | 18.53±0.12 | 94±0.87 | 28.4±0.12 | 1.38±0.66 | 29±0.12 | 24.6±0.69 | 44.46±0.12 |
| RIL-18 | *Rf3Rf3/ rf4rf4* | 124±0.22 | 12.35±0.47 | 346±0.31 | 325±0.09 | 18±0.42 | 14.56±0.36 | 95±0.65 | 24.1±0.43 | 1.67±0.32 | 13±0.64 | 25.06±0.45 | 50.83±0.69 |
| RIL-19 | *rf3rf3/ Rf4Rf4* | 120±0.15 | 11.7±0.69 | 356±0.12 | 340±0.39 | 22±0.41 | 14.7±0.74 | 93±0.12 | 29.13±0.78 | 1.47±0.14 | 22±0.13 | 22.47±0.36 | 39.1±0.77 |
| RIL-20 | *Rf3Rf3/Rf4Rf4* | 99±0.44 | 11.6±0.77 | 399±0.40 | 359±0.44 | 20±0.05 | 15.22±0.33 | 101±0.88 | 16.72±0.32 | 1.26±0.47 | 18±0.66 | 25.6±0.78 | 31.6±0.33 |
| RIL-21 | *Rf3Rf3/Rf4Rf4* | 97±0.12 | 11.54±0.33 | 322±0.39 | 314±0.11 | 14±0.33 | 14.54±0.45 | 95.5±0.65 | 26.7±0.12 | 1.16±0.69 | 17±0.74 | 20.73±0.21 | 28.04±0.35 |
| RIL-22 | *Rf3Rf3/Rf4Rf4* | 105±0.89 | 11.4±0.42 | 366±0.11 | 322±0.36 | 23±0.33 | 14.4±0.74 | 92±0.33 | 19.01±0.32 | 1.51±0.65 | 29±0.36 | 25.4±0.17 | 55.28±0.04 |
| RIL-23 | *Rf3Rf3/Rf4Rf4* | 104±0.21 | 10.45±0.55 | 300±0.65 | 280±0.45 | 20±0.78 | 15.22±0.13 | 125±0.44 | 31.5±0.88 | 1.5±0.98 | 10±0.77 | 18.96±0.69 | 36.28±0.45 |
| RIL-24 | *Rf3Rf3/ rf4rf4* | 107±0.15 | 10.01±0.78 | 313±0.26 | 220±0.12 | 20±0.36 | 16.89±0.45 | 115±0.44 | 31.23±0.97 | 1.52±0.45 | 12±0.56 | 20±0.78 | 31.32±0.12 |
| RIL-25 | *Rf3Rf3/Rf4Rf4* | 109±0.23 | 18.03±0.45 | 365±0.11 | 263±0.72 | 20±0.36 | 17.56±0.45 | 120.1±0.36 | 29.53±0.69 | 1.67±0.25 | 15±0.45 | 21.32±0.71 | 54.16±0.69 |

| Supplementary Table 1 (continued) | | | | | | | | | | | | | |
| --- | --- | --- | --- | --- | --- | --- | --- | --- | --- | --- | --- | --- | --- |
| Material | R*f*3/R*f*4 status | DFF±SE | YLD±SE | GP±SE | FGP±SE | TGW±SE | PW±SE | PH±SE | FLL±SE | FLW±SE | PT±SE | PL±SE | BM±SE |
| RIL-26 | *Rf3Rf3/Rf4Rf4* | 107±0.44 | 23.94±0.21 | 345±0.45 | 300±0.36 | 20±0.14 | 20.88±0.36 | 123±0.69 | 31.73±0.44 | 1.64±0.11 | 11±0.36 | 22.63±0.47 | 44±0.36 |
| RIL-27 | *Rf3Rf3/ rf4rf4* | 102±0.36 | 27.88±0.56 | 456±0.36 | 389±0.45 | 20±0.45 | 17.55±0.23 | 116±0.45 | 28.3±0.36 | 1.47±0.48 | 13±0.79 | 18.45±0.36 | 28.02±0.98 |
| RIL-28 | *Rf3Rf3/Rf4Rf4* | 104±0.11 | 30.79±0.48 | 356±0.25 | 320±0.45 | 19±0.14 | 18.99±0.78 | 129.2±0.36 | 37.5±0.22 | 1.6±0.89 | 15±0.21 | 16±0.45 | 49.62±0.11 |
| RIL-29 | *Rf3Rf3/Rf4Rf4* | 105±0.36 | 21.26±0.55 | 399±0.45 | 378±0.36 | 20±0.55 | 16.26±0.23 | 119±0.11 | 32.3±0.21 | 1.68±0.36 | 10±0.85 | 16.26±0.36 | 40.7±0.23 |
| RIL-30 | *Rf3Rf3/ rf4rf4* | 102±0.22 | 21.69±0.11 | 346±0.65 | 285±0.87 | 21±0.19 | 16.69±0.59 | 119±0.54 | 31.73±0.23 | 1.66±0.85 | 10±0.36 | 16.69±0.33 | 25.87±0.36 |
| RIL-31 | *Rf3Rf3/Rf4Rf4* | 101±0.28 | 25.1±0.36 | 344±0.23 | 312±0.56 | 20±0.12 | 20.56±0.36 | 109.2±0.22 | 27.1±0.45 | 1.52±0.36 | 9±0.88 | 20.1±0.58 | 36.22±0.21 |
| RIL-32 | *Rf3Rf3/Rf4Rf4* | 103±0.37 | 22.52±0.11 | 412±0.75 | 364±0.22 | 22±0.36 | 17.52±0.33 | 121.5±0.45 | 27.7±0.36 | 1.45±0.11 | 11±0.25 | 17.52±0.45 | 50.46±0.32 |
| RIL-33 | *Rf3Rf3/Rf4Rf4* | 106±0.36 | 27.89±0.45 | 488±0.11 | 456±0.15 | 20±0.77 | 21.36±0.66 | 121±0.32 | 29±0.12 | 1.46±0.36 | 12±0.33 | 22.89±0.12 | 37.62±0.18 |
| RIL-34 | *Rf3Rf3/Rf4Rf4* | 104±0.23 | 20±0.22 | 378±0.45 | 320±0.36 | 22±0.12 | 17.88±0.36 | 123±0.78 | 30.56±0.38 | 1.43±0.15 | 10±0.34 | 15±0.12 | 27.32±0.89 |
| RIL-35 | *rf3rf3/ Rf4Rf4* | 96±0.12 | 24.58±0.15 | 490±0.66 | 425±0.11 | 17±0.14 | 15.68±0.28 | 116±0.68 | 23.5±0.47 | 1.33±0.34 | 11±0.32 | 19.58±0.11 | 23±0.32 |
| RIL-36 | *rf3rf3/ Rf4Rf4* | 100±0.23 | 23.26±0.11 | 386±0.45 | 356±0.48 | 19±0.12 | 18.56±0.37 | 117±0.21 | 31.86±0.82 | 1.51±0.32 | 10±0.11 | 18.26±0.39 | 16.91±0.11 |
| RIL-37 | *rf3rf3/ Rf4Rf4* | 108±0.30 | 18.75±0.22 | 366±0.32 | 345±0.14 | 17±0.21 | 17.89±0.32 | 109±0.28 | 20.06±0.21 | 1.68±0.33 | 11±0.22 | 13.75±0.19 | 43.08±0.27 |
| RIL-38 | *rf3rf3/ rf4rf4* | 111±0.27 | 14.86±0.45 | 345±0.45 | 320±0.66 | 15±0.52 | 12.36±0.44 | 129±0.32 | 37.53±0.31 | 1.64±0.21 | 12±0.33 | 9.86±0.25 | 29.92±0.29 |
| RIL-39 | *Rf3Rf3/ rf4rf4* | 108±0.56 | 25.11±0.58 | 366±0.45 | 345±0.33 | 19±0.35 | 14.56±0.48 | 114±0.98 | 30±1.22 | 1.56±0.77 | 10±0.33 | 20.11±0.37 | 33.72±0.38 |
| RIL-40 | *Rf3Rf3/Rf4Rf4* | 106±0.22 | 29.61±0.35 | 688±0.11 | 362±0.58 | 21±0.87 | 18.29±0.88 | 125±0.80 | 32.36±0.65 | 1.65±0.81 | 10±0.26 | 24.69±0.36 | 25.43±0.33 |
| RIL-41 | *Rf3Rf3/Rf4Rf4* | 103±0.23 | 21.68±0.36 | 399±0.45 | 388±0.81 | 21±0.37 | 16.63±0.23 | 115±0.39 | 40.66±0.42 | 1.4±0.95 | 12±0.36 | 16.68±0.22 | 25.4±0.15 |
| RIL-42 | *Rf3Rf3/Rf4Rf4* | 104±0.52 | 22.26±0.33 | 326±0.87 | 315±0.22 | 22±0.12 | 16.54±0.32 | 121.5±0.24 | 28.45±0.47 | 1.6±0.22 | 10±0.44 | 17.26±0.56 | 32.82±0.20 |
| RIL-43 | *Rf3Rf3/ rf4rf4* | 99±0.22 | 19.51.±0.32 | 388±0.25 | 360±0.56 | 21±0.77 | 16.77±0.85 | 120.5±0.45 | 28.46±0.36 | 1.58±0.28 | 12±0.33 | 14.51±0.32 | 30.32±0.56 |
| RIL-44 | *Rf3Rf3/Rf4Rf4* | 103±0.79 | 20.81±0.37 | 389±0.89 | 320±0.36 | 20±1.03 | 12.36±0.33 | 116.7±0.69 | 30.93±0.37 | 1.4±0.31 | 13±0.33 | 15.81±0.21 | 30.36±0.12 |
| RIL-45 | *Rf3Rf3/Rf4Rf4* | 102±0.32 | 22.36±0.22 | 356±0.78 | 330±0.44 | 19±0.32 | 17.56±0.36 | 123.2±0.45 | 30.76±0.22 | 1.52±0.85 | 12±0.12 | 17.36±0.31 | 33.52±0.15 |
| RIL-46 | *Rf3Rf3/Rf4Rf4* | 103±0.22 | 21.56±0.13 | 345±0.25 | 320±0.37 | 21±0.11 | 18.56±0.31 | 119±0.12 | 28.36±0.11 | 1.51±0.45 | 10±0.69 | 16.56±0.14 | 29.72±0.36 |
| RIL-47 | *Rf3Rf3/Rf4Rf4* | 101±0.21 | 21.79±0.78 | 322±0.12 | 312±0.89 | 20±0.36 | 19.77±0.45 | 120±0.44 | 30.2±0.75 | 1.6±0.12 | 10±0.36 | 16.79±0.12 | 34.78±0.33 |
| RIL-48 | *Rf3Rf3/Rf4Rf4* | 101±0.85 | 22.1±0.12 | 378±0.33 | 369±0.12 | 17±0.38 | 15.1±0.11 | 112±0.12 | 29.46±0.18 | 1.56±0.37 | 11±0.45 | 17.1±0.85 | 45.3±0.14 |
| RIL-49 | *rf3rf3/ rf4rf4* | 101±0.15 | 21.48±0.36 | 369±0.85 | 348±0.15 | 17±0.38 | 14.48±0.85 | 120±0.75 | 27.93±0.88 | 1.5±0.85 | 12±0.42 | 16.48±0.35 | 32.42±0.75 |
| RIL-50 | *Rf3Rf3/Rf4Rf4* | 101±0.21 | 23.58±0.87 | 355±0.11 | 310±0.33 | 22±0.36 | 16.58±0.89 | 115±0.32 | 31.76±0.33 | 1.72±0.57 | 12±0.69 | 18.58±0.42 | 29.48±0.79 |
| RIL-51 | *Rf3Rf3/Rf4Rf4* | 106±0.23 | 21.44±0.85 | 399±0.12 | 366±0.32 | 22±0.12 | 14.44±0.89 | 110±0.37 | 32.5±0.85 | 1.62±0.12 | 11±0.19 | 16.44±0.96 | 30.08±0.36 |
| RIL-52 | *Rf3Rf3/Rf4Rf4* | 110±0.22 | 20.34±0.32 | 350±0.28 | 336±0.36 | 23±0.48 | 13.34±0.85 | 115±0.65 | 31±0.45 | 1.53±0.69 | 11±0.21 | 15.34±0.36 | 34.7±0.22 |
| RIL-53 | *Rf3Rf3/Rf4Rf4* | 111±0.65 | 26.07±0.32 | 389±0.31 | 320±0.37 | 22±0.32 | 19.07±0.21 | 124±0.22 | 30.56±0.21 | 1.66±0.38 | 10±0.65 | 21.07±0.12 | 36.1±0.33 |
| RIL-54 | *Rf3Rf3/Rf4Rf4* | 106±0.33 | 27.28±0.25 | 366±0.21 | 321±0.25 | 19±0.36 | 20.28±0.38 | 115±0.38 | 31.53±0.25 | 1.62±0.73 | 12±1.12 | 22.28±0.22 | 40.16±0.36 |
| RIL-55 | *Rf3Rf3/Rf4Rf4* | 106±0.25 | 20.7±0.34 | 378±0.29 | 356±0.37 | 23±0.33 | 13.7±0.12 | 117±0.33 | 30.93±0.21 | 1.68±0.33 | 10±0.31 | 15.7±0.21 | 45±0.30 |
| RIL-56 | *rf3rf3/ Rf4Rf4* | 102±0.65 | 34.44±0.12 | 355±0.85 | 320±0.25 | 18±0.22 | 27.44±0.99 | 112±1.22 | 30.02±0.75 | 1.48±0.36 | 12±0.21 | 29.44±0.36 | 57±0.31 |
| RIL-57 | *Rf3Rf3/Rf4Rf4* | 107±0.23 | 19.36±0.25 | 312±0.38 | 286±0.38 | 18±0.21 | 12.36±0.21 | 112±0.32 | 33.2±0.32 | 1.64±0.85 | 10±0.19 | 14.36±0.16 | 33.44±0.28 |
| RIL-58 | *Rf3Rf3/Rf4Rf4* | 96±0.35 | 14.78±0.36 | 356±0.39 | 330±0.78 | 22±0.77 | 17.78±0.36 | 109±0.85 | 26.83±0.69 | 1.45±0.15 | 10±0.45 | 9.78±0.85 | 35.22±0.33 |
| RIL-59 | *Rf3Rf3/Rf4Rf4* | 101±0.78 | 27.47±0.33 | 377±0.28 | 356±0.29 | 16±0.12 | 20.47±0.36 | 116±0.75 | 31.35±0.36 | 1.55±0.89 | 11±0.96 | 22.47±0.44 | 34±0.36 |
| RIL-60 | *rf3rf3/ rf4rf4* | 102±0.33 | 24.46±0.45 | 384±0.36 | 325±0.21 | 22±0.69 | 17.46±0.33 | 124±0.75 | 31.23±0.36 | 1.76±0.78 | 12±0.88 | 19.46±0.12 | 37.9±0.96 |
| Supplementary Table 1 (continued) | | | | | | | | | | | | | |
| Material | R*f*3/R*f*4 status | DFF±SE | YLD±SE | GP±SE | FGP±SE | TGW±SE | PW±SE | PH±SE | FLL±SE | FLW±SE | PT±SE | PL±SE | BM±SE |
| RIL-61 | *Rf3Rf3/Rf4Rf4* | 105±0.22 | 26.13±0.12 | 388±0.15 | 369±0.78 | 22±0.36 | 19.13±0.37 | 96±0.21 | 23.65±0.33 | 1.67±0.12 | 13±0.32 | 21.13±0.19 | 35.16±0.38 |
| RIL-62 | *Rf3Rf3/Rf4Rf4* | 99±0.24 | 26.97±0.39 | 356±0.33 | 322±0.48 | 21±0.43 | 19.97±0.22 | 125±0.31 | 29.33±0.75 | 1.6±0.36 | 10±0.12 | 21.97±0.32 | 39.64±0.12 |
| RIL-63 | *rf3rf3/ Rf4Rf4* | 105±0.31 | 22.59±0.32 | 399±0.15 | 356±0.32 | 20±0.31 | 15.59±0.22 | 112.5±0.32 | 22.4±0.12 | 1.67±0.32 | 12±0.22 | 17.59±0.87 | 46.52±0.33 |
| RIL-64 | *Rf3Rf3/Rf4Rf4* | 102±0.23 | 19.81±0.21 | 345±0.89 | 321±0.37 | 22±0.39 | 12.81±0.37 | 129.5±0.69 | 31.6±0.45 | 1.75±0.21 | 10±0.36 | 14.81±0.36 | 32.06±0.58 |
| RIL-65 | *Rf3Rf3/ rf4rf4* | 105±0.11 | 22.72±0.58 | 369±0.45 | 355±0.87 | 22±0.45 | 15.72±0.36 | 102±0.47 | 31±0.88 | 1.74±0.69 | 12±0.48 | 17.72±0.69 | 35.34±0.42 |
| RIL-66 | *Rf3Rf3/Rf4Rf4* | 105±0.32 | 21.25±0.36 | 312±0.42 | 300±0.22 | 20±0.21 | 14.25±0.35 | 109±0.44 | 30±0.58 | 1.7±0.45 | 12±0.88 | 16.25±0.47 | 26.96±0.99 |
| RIL-67 | *rf3rf3/ Rf4Rf4* | 105±1.02 | 21.3±0.47 | 325±0.86 | 300±0.98 | 21±0.02 | 14.3±0.56 | 115.6±0.41 | 28.4±0.75 | 1.61±0.36 | 10±0.47 | 16.3±0.32 | 29.1±0.39 |
| RIL-68 | *Rf3Rf3/Rf4Rf4* | 107±0.32 | 32.42±0.42 | 369±0.43 | 320±0.14 | 20±0.36 | 25.42±0.49 | 105±0.33 | 29.4±0.13 | 1.56±0.14 | 13±0.58 | 27.42±0.36 | 30.8±0.14 |
| RIL-69 | *Rf3Rf3/Rf4Rf4* | 108±0.31 | 17.98±0.45 | 358±0.77 | 345±0.40 | 19±0.31 | 10.98±0.17 | 100±0.36 | 28.26±0.85 | 1.52±0.43 | 12±0.12 | 12.98±0.37 | 22.98±0.96 |
| RIL-70 | *Rf3Rf3/Rf4Rf4* | 101±0.13 | 23.5±0.42 | 346±0.33 | 325±0.47 | 20±0.18 | 16.5±0.36 | 100±0.78 | 30.33±0.69 | 1.53±0.78 | 12±0.47 | 18.5±0.33 | 35.16±0.05 |
| RIL-71 | *rf3rf3/ rf4rf4* | 112±0.23 | 24.34±0.02 | 347±0.14 | 320±0.56 | 18±0.58 | 17.34±0.47 | 101±0.70 | 30.75±0.45 | 1.65±0.36 | 10±0.12 | 19.34±0.25 | 53.7±0.45 |
| RIL-72 | *Rf3Rf3/Rf4Rf4* | 101±0.85 | 21.63±0.36 | 369±0.78 | 321±0.45 | 22±0.89 | 14.63±0.36 | 97±0.47 | 28.66±0.36 | 1.6±0.22 | 10±0.32 | 16.63±0.12 | 29.83±0.56 |
| RIL-73 | *rf3rf3/ Rf4Rf4* | 103±0.36 | 18.39±0.22 | 355±0.37 | 322±0.36 | 17±0.23 | 11.39±0.22 | 100±0.89 | 31.53±0.01 | 1.51±0.56 | 11±0.86 | 13.39±0.49 | 32.4±0.31 |
| RIL-74 | *Rf3Rf3/Rf4Rf4* | 115±0.33 | 20.15±0.37 | 369±0.21 | 355±0.33 | 20±0.15 | 13.15±0.56 | 95±0.31 | 30.13±0.33 | 1.49±0.11 | 12±0.33 | 15.15±0.31 | 31.98±0.98 |
| RIL-75 | *Rf3Rf3/Rf4Rf4* | 102±0.11 | 23.9±0.78 | 378±0.12 | 325±0.36 | 21±0.65 | 16.9±0.77 | 101±0.39 | 29.66±0.12 | 1.67±0.31 | 10±0.74 | 18.9±0.99 | 41.04±1.01 |
| RIL-76 | *rf3rf3/ rf4rf4* | 125±0.12 | 30.18±0.31 | 369±0.55 | 321±0.31 | 15±0.12 | 23.18±0.45 | 97.6±0.36 | 29.7±0.12 | 1.68±0.45 | 13±0.55 | 25.18±0.45 | 39.68±0.32 |
| RIL-77 | *Rf3Rf3/ rf4rf4* | 107±0.11 | 24.14±0.54 | 358±0.47 | 329±0.30 | 22±0.45 | 17.44±0.78 | 103±0.65 | 31.8±0.44 | 1.72±0.33 | 12±0.12 | 19.14±0.85 | 27.32±0.31 |
| RIL-78 | *Rf3Rf3/Rf4Rf4* | 115±0.22 | 18.82±0.14 | 369±0.56 | 322±0.33 | 20±0.21 | 11.82±0.45 | 105±0.33 | 32.1±0.47 | 1.33±0.25 | 10±0.69 | 13.82±0.87 | 24.28±0.14 |
| RIL-79 | *Rf3Rf3/ rf4rf4* | 105±0.41 | 19.68±0.22 | 378±0.32 | 360±0.14 | 22±0.36 | 12.68±0.45 | 99±0.36 | 32.57±0.75 | 1.54±0.36 | 10±0.78 | 14.68±0.44 | 29.84±0.36 |
| RIL-80 | *Rf3Rf3/Rf4Rf4* | 97±0.12 | 21.56±0.32 | 355±0.02 | 322±0.45 | 20±0.56 | 14.56±0.55 | 99±0.31 | 37.9±0.33 | 1.81±0.12 | 9±0.22 | 16.56±0.40 | 30.54±0.12 |
| RIL-81 | *Rf3Rf3/Rf4Rf4* | 101±0.33 | 21.65±0.31 | 321±0.11 | 300±0.25 | 21±0.20 | 14.65±0.45 | 125.8±0.69 | 33.2±0.77 | 1.68±0.75 | 11±0.36 | 16.65±0.23 | 26.16±0.02 |
| RIL-82 | *Rf3Rf3/Rf4Rf4* | 105±0.05 | 19.88±0.55 | 325±0.78 | 312±0.75 | 20±0.36 | 12.88±0.11 | 94±0.33 | 35.45±0.12 | 1.53±0.66 | 10±0.75 | 14.88±0.12 | 27.08±0.56 |
| RIL-83 | *Rf3Rf3/Rf4Rf4* | 107±0.36 | 20.06±0.12 | 355±0.45 | 322±0.55 | 23±0.31 | 13.06±0.25 | 99±0.36 | 32.13±0.44 | 1.56±0.45 | 11±0.89 | 15.06±0.77 | 22.54±0.42 |
| RIL-84 | *Rf3Rf3/Rf4Rf4* | 107±0.30 | 15.6±0.23 | 344±0.12 | 306±0.11 | 21±0.30 | 8.6±0.86 | 99±0.44 | 22±0.23 | 1.65±0.33 | 13±0.22 | 10.6±0.45 | 23.4±0.33 |
| RIL-85 | *Rf3Rf3/Rf4Rf4* | 105±0.12 | 15.93±0.33 | 356±0.78 | 312±0.36 | 19±0.78 | 8.93±0.23 | 100±0.11 | 21.4±0.89 | 1.44±0.65 | 11±0.33 | 10.93±0.30 | 30.62±0.22 |
| RIL-86 | *Rf3Rf3/Rf4Rf4* | 101±0.56 | 14.97±0.55 | 345±0.23 | 322±0.15 | 21±0.86 | 7.97±0.96 | 107.5±0.36 | 25.1±0.01 | 13.38±0.34 | 10±0.12 | 19.97±0.69 | 15.22±0.77 |
| RIL-87 | *Rf3Rf3/Rf4Rf4* | 107±0.33 | 19.05±0.25 | 355±0.31 | 312±0.14 | 16±1.01 | 12.05±0.37 | 98±0.33 | 20.8±0.21 | 1.52±0.31 | 12±0.22 | 14.05±0.55 | 22.16±0.65 |
| RIL-88 | *Rf3Rf3/Rf4Rf4* | 108±0.37 | 14.76±0.27 | 369±0.54 | 355±0.66 | 20±0.14 | 7.76±0.21 | 99±0.56 | 20.6±0.58 | 1.24±0.21 | 10±0.20 | 19.76±0.48 | 17.34±0.15 |
| RIL-89 | *Rf3Rf3/Rf4Rf4* | 103±0.15 | 16.73±0.36 | 345±0.78 | 322±0.89 | 22±0.44 | 9.73±0.33 | 105.4±0.75 | 28.5±0.25 | 1.45±0.14 | 9±0.33 | 11.73±0.47 | 18.5±0.58 |
| RIL-90 | *Rf3Rf3/Rf4Rf4* | 107±0.47 | 19.54±0.58 | 366±0.14 | 330±0.15 | 21±0.77 | 12.54±0.21 | 103.6±0.26 | 29.96±0.25 | 1.46±0.14 | 10±0.83 | 14.54±0.74 | 31.56±0.23 |
| RIL-91 | *Rf3Rf3/Rf4Rf4* | 101±0.89 | 17.64±0.36 | 345±0.21 | 326±0.35 | 20±0.75 | 10.64±0.21 | 101±0.14 | 27.2±0.15 | 1.41±0.58 | 13±0.15 | 12.64±0.36 | 29.76±0.21 |
| RIL-92 | *rf3rf3/ Rf4Rf4* | 102±0.25 | 24.83±0.31 | 347±0.25 | 322±0.87 | 22±0.65 | 17.83±0.23 | 102±0.27 | 25.85±0.36 | 1.52±0.33 | 10±0.88 | 19.83±0.87 | 32.72±0.65 |
| RIL-93 | *rf3rf3/ rf4rf4* | 96±0.36 | 23.08±0.75 | 358±0.46 | 321±0.32 | 19±0.21 | 16.08±0.58 | 105.9±0.33 | 21.16±0.24 | 1.32±0.78 | 10±0.33 | 18.08±0.37 | 21.42±0.65 |
| RIL-94 | *Rf3Rf3/Rf4Rf4* | 97±0.88 | 22±0.96 | 369±0.93 | 321±0.33 | 20±0.34 | 15±0.35 | 101±0.75 | 22.4±0.98 | 1.2±0.96 | 12±0.36 | 17±0.38 | 22.92±0.78 |
| RIL-95 | *Rf3Rf3/Rf4Rf4* | 94±0.21 | 19.26±0.33 | 366±0.66 | 348±0.88 | 16±0.69 | 12.26±0.75 | 103±0.31 | 27.3±0.35 | 1.33±0.39 | 10±0.35 | 14.26±0.75 | 27.14±0.33 |
| Supplementary Table 1 (continued) | | | | | | | | | | | | | |
| Material | R*f*3/R*f*4 status | DFF±SE | YLD±SE | GP±SE | FGP±SE | TGW±SE | PW±SE | PH±SE | FLL±SE | FLW±SE | PT±SE | PL±SE | BM±SE |
| RIL-96 | *Rf3Rf3/ rf4rf4* | 99±0.33 | 27.3±0.21 | 322±0.55 | 310±0.36 | 21±0.88 | 20.3±0.33 | 114.7±0.87 | 25.5±0.69 | 1.46±0.67 | 10±0.36 | 22.3±0.87 | 29.12±0.22 |
| RIL-97 | *Rf3Rf3/Rf4Rf4* | 102±0.39 | 21.22±0.37 | 323±0.68 | 300±0.21 | 21±0.31 | 14.22±0.25 | 112.3±0.36 | 27.8±0.99 | 1.44±1.03 | 12±1.66 | 16.22±1.01 | 30.44±0.39 |
| RIL-98 | *Rf3Rf3/Rf4Rf4* | 105±0.33 | 19.29±0.87 | 331±0.25 | 300±0.56 | 20±0.33 | 12.29±0.58 | 104±0.89 | 35.4±0.85 | 1.53±0.36 | 12±0.33 | 14.29±0.33 | 35.32±0.30 |
| RIL-99 | *Rf3Rf3/Rf4Rf4* | 101±0.32 | 16.95±0.23 | 355±0.36 | 320±0.21 | 20±0.88 | 19.95±0.86 | 104±0.33 | 36.3±0.86 | 1.55±0.34 | 12±0.31 | 11.95±0.54 | 37.36±0.69 |
| RIL-100 | *Rf3Rf3/Rf4Rf4* | 106±0.31 | 19.51±0.69 | 365±0.98 | 322±0.39 | 21±0.75 | 12.51±0.96 | 102±0.98 | 22.8±0.64 | 1.6±0.75 | 10±1.03 | 14.51±0.01 | 45±0.33 |
| RIL-101 | *Rf3Rf3/Rf4Rf4* | 105±0.68 | 14.62±0.64 | 345±0.31 | 321±0.65 | 20±0.69 | 17.62±1.03 | 103±0.69 | 30.53±0.98 | 1.5±0.38 | 11±0.69 | 19±0.36 | 23.3±0.37 |
| RIL-102 | *Rf3Rf3/Rf4Rf4* | 102±0.15 | 25.17±0.55 | 369±0.22 | 322±0.36 | 21±0.22 | 18.17±0.56 | 105±0.37 | 26.5±0.33 | 1.54±0.45 | 10±0.64 | 20.17±0.86 | 37.8±0.33 |
| RIL-103 | *Rf3Rf3/Rf4Rf4* | 100±0.25 | 25.12±0.31 | 356±1.03 | 312±0.78 | 20±0.37 | 18.12±0.69 | 113±0.85 | 26.1±0.12 | 1.54±0.33 | 12±0.31 | 20.12±0.88 | 35.08±0.39 |
| RIL-104 | *Rf3Rf3/Rf4Rf4* | 111±0.36 | 19.07±0.30 | 355±0.11 | 321±0.48 | 20.4±0.12 | 12.07±0.44 | 106±0.31 | 31.7±0.96 | 1.38±0.12 | 13±0.75 | 14.07±0.69 | 25.92±0.35 |
| RIL-105 | *Rf3Rf3/Rf4Rf4* | 108±0.37 | 17.15±0.22 | 369±0.18 | 340±0.39 | 16±0.38 | 10.15±0.66 | 118±0.33 | 36.36±0.36 | 1.65±0.39 | 10±0.33 | 12.15±0.86 | 57.04±0.86 |
|  | Mean | 104.52 | 105.15 | 28.77 | 1.54 | 21.41 | 24.26 | 13.95 | 20.82 | 353.48 | 323.64 | 20.03 | 31.32 |
|  | SD | 5.50 | 8.00 | 4.47 | 0.14 | 3.70 | 1.32 | 3.93 | 4.01 | 19.05 | 15.75 | 1.79 | 8.98 |
|  | Std error | 3.18 | 4.62 | 2.58 | 0.08 | 2.14 | 0.76 | 2.27 | 2.32 | 11.00 | 9.09 | 1.03 | 5.19 |
|  | CV(%) | 5.27 | 7.61 | 15.54 | 8.81 | 17.28 | 5.46 | 28.15 | 19.26 | 5.39 | 4.87 | 8.91 | 28.68 |
|  | Heritability (H^2^)% | 92.66 | 98.44 | 97.42 | 95.12 | 98.23 | 97.78 | 96.47 | 93.45 | 97.48 | 86.55 | 94.58 | 38.99 |

DFF-Days to 50% percent flowering; YLD-Total grain yield/plant(g); GP-Total grains/panicle; FGP-Fertile grains/panicle; TGW-Test (1000) grain weight (g); PW-Panicle weight (g); PH-Plant height (cm);FLL-Flag leaf length (cm); FLW-Flag leaf width (cm); PT-Productive tillers; PL-Panicle length (cm); BM-Biomass (g), SE-Standard Error, SD-Standard Deviation, CV%-Coefficient of Variation (%).

| **Supplementary Table 2**: Pooled analysis of variance (ANOVA) for yield and yield related traits in rice | | | | | | | | | | | | |
| --- | --- | --- | --- | --- | --- | --- | --- | --- | --- | --- | --- | --- |
| Source of variation | DFF | FGP | GP | PH | PL | PT | PW | TGW | BM | FLL | FLW | YLD |
| Year | 1824.9** | 10037.45** | 17512.77** | 56285.03** | 16670.18** | 8916.55** | 7080.8** | 400.14** | 16906.02** | 5979.45** | 8480.56** | 21909.17** |
| REP (year) | 132.06** | 23.07** | 9.54** | 15.46** | 11.25** | 13.22** | 4.39** | 21.28** | 1NS | 94.13** | 8.75** | 51.36** |
| Gen | 1290.98** | 103.19** | 56.09** | 538.38** | 31.31** | 81.54** | 231.47** | 45.32** | 655.05** | 247.23** | 9.07** | 294.51** |
| Year * Gen | 1.68** | 1.70** | 2.28** | 5.65** | 1.59** | 3.16** | 3.81** | 1* | 14.36** | 0.98* | 1.47** | 12.95** |

** - Significant at 1%, * - Significant at 5%, NS - Non Significant; F value and its significance provided in the table ; DFF-Days to 50% percent flowering; FGP-Fertile grains/panicle; GP-Total grains/panicle; PH-Plant height (cm); PL-Panicle length; PT-Number of productive tillers; PW-Panicle weight (g); TGW-Test (1,000) grain weight (g); BM-Biomass (g); FLL-Flag leaf length (cm); FLW-Flag leaf width (cm); YLD-Total grain yield/plant (g); REP-Replication; Gen-Genotype; Year * Gen-Year * Genotype.

**Supplementary Table 3:** Descriptive statistics table for 12 agronomical traits in 105 RILs

|  | Std. Dev | W value | Pr value | C.V(%) | Mean | P-value | Skewness | Kurtosis | Range |
| --- | --- | --- | --- | --- | --- | --- | --- | --- | --- |
| DFF | 5.45 | 0.92 | 0.00 | 2.03 | 104.26 | 0.00 | 1.22 | 2.86 | 93-125 |
| YLD | 5.37 | 0.97 | 0.09 | 2.95 | 21.83 | 0.04 | 1.22 | 2.86 | 11.4-36.48 |
| GP | 9.79 | 0.87 | 0.00 | 6.48 | 360.04 | 0.00 | 1.56 | 1.49 | 300-490 |
| FGP | 3.63 | 0.88 | 0.08 | 1.08 | 327.05 | 0.00 | 0.89 | 1.80 | 220-456 |
| TGW | 2.18 | 0.92 | 0.00 | 3.53 | 20.02 | 0.00 | 0.80 | 0.41 | 14-24 |
| PW | 5.11 | 0.98 | 0.00 | 4.16 | 15.49 | 0.09 | 0.02 | 0.13 | 14.4-29.48 |
| PH | 9.9 | 0.94 | 0.00 | 4.50 | 109.98 | 0.00 | 0.12 | -1.11 | 94-129.5 |
| FLL | 4.37 | 0.96 | 0.00 | 1.46 | 29.06 | 0.00 | -0.42 | 0.62 | 16.72-40.66 |
| FLW | 0.13 | 0.97 | 0.00 | 0.92 | 1.54 | 0.05 | -0.54 | 0.18 | 1.16-1.81 |
| PT | 1.32 | 0.88 | 0.00 | 6.43 | 10.99 | 0.00 | 0.58 | 0.28 | 8-15 |
| PL | 5.31 | 0.98 | 0.00 | 3.78 | 17.02 | 0.07 | 0.09 | -0.13 | 16.4-31.48 |
| BM | 9.06 | 0.95 | 0.90 | 4.71 | 33.47 | 0.00 | 0.65 | 0.16 | 15.22-57.04 |

DFF-Days to 50% percent flowering; YLD-Total grain yield/plant(g); GP-Total grains/panicle; FGP-Fertile grains/panicle; TGW-Test (1000) grain weight (g); PW-Panicle weight (g); PH-Plant height (cm);FLL-Flag leaf length (cm); FLW-Flag leaf width (cm); PT-Productive tillers; PL-Panicle length (cm); BM-Biomass (g)

Std. Dev-Standard deviation; W value-Kendall's coefficient of concordance, as the W values of all the traits are observed to be nearing 1, this indicates the uniformity among the replications considered for data recording; Pr value-Predictor value; C.V(%)-Coefficient of variation in percentage; P-value-as statistical P value is typically ≤ 0.05, this indicates the significance of the data used for statistical analysis; Skewness- as the values for skewness are in between -1.96 and +1.96, the distribution of the data is therefore observed to be univariate; Kurtosis- As the values of kurtosis are less than 3, the dataset is observed to follow a normal distribution. Value ranges-of the each trait observations is indicated.

**Supplementary Table 4:**  Phenotypic correlation (upper diagonal)-Genotypic correlation (lower diagonal) among 105 recombinant inbred line (RIL) population among agro-morphological traits

|  | DFF | YLD | GP | FGP | TGW | PW | PH | FLL | FLW | PT | PL | BM |
| --- | --- | --- | --- | --- | --- | --- | --- | --- | --- | --- | --- | --- |
| DFF | 1 | -0.24 | -0.05 | -0.07 | -0.08 | -0.2 | -0.15 | 0.12 | 0.17 | 0.02 | -0.22 | 0.22 |
| YLD | -0.49 | 1 | 0.16* | 0.13* | 0.14* | 0.89** | 0.35** | 0.23* | 0.29* | 0.22* | 0.95* | 0.04 |
| GP | -0.08 | 0.21* | 1 | 0.82** | 0 | 0.13 | 0.09 | -0.14 | -0.11 | 0.14 | 0.12 | 0.06 |
| FGP | -0.02 | 0.19 | 0.72** | 1 | -0.03 | 0.06 | 0.04 | -0.07 | -0.14 | 0.01 | 0.02 | 0.01 |
| TGW | 0.01 | 0.41** | 0.3 | 0.17 | 1 | 0.12 | 0.16 | 0.06 | 0.18 | -0.05 | 0.16 | -0.1 |
| PW | -0.4 | 0.85** | -0.15 | -0.22 | 0.42** | 1 | 0.37 | 0.15 | 0.3 | 0.23* | 0.92** | 0.11 |
| PH | -0.2 | 0.62** | -0.03 | -0.04 | 0.55** | 0.51** | 1 | 0.37** | 0.29** | 0.07 | 0.34** | 0.14 |
| FLL | 0.07 | 0.52** | -0.29 | -0.16 | 0.17 | 0.45** | 0.57** | 1 | 0.33** | 0.08 | 0.21 | 0.03 |
| FLW | 0.09 | 0.44** | 0.03 | -0.15 | 0.3 | 0.31** | 0.5** | 0.28** | 1 | 0.02 | 0.31** | 0.36** |
| PT | -0.45 | 0.42** | 0.08 | 0.24** | 0.31** | 0.47** | 0.23** | 0.16* | -0.05 | 1 | 0.23* | 0.09 |
| PL | -0.49 | 0.85** | -0.08 | -0.05 | 0.41** | 0.85** | 0.62** | 0.52** | 0.44* | 0.42* | 1 | 0.05 |
| BM | 0.15 | 0.03 | 0.08 | -0.13 | 0.28** | 0.3** | -0.11 | -0.14 | 0.35** | 0.12 | 0.03 | 1 |

DFF-Days to 50% percent flowering; YLD-Total grain yield/plant(g); GP-Total Grains/panicle; FGP-Fertile grains/panicle; TGW-Test (1000) grain weight (g); PW-Panicle weight (g);

PH-Plant height (cm); FLL-Flag leaf length (cm); FLW-Flag leaf width (cm); PT-Number of productive tillers; PL-Panicle length; BM-Biomass. * significant at 5% level of significance,

** significant at 1% level of significance

In 105RILs, strong and positive phenotypic correlation was observed between yield and its crucial allied parameters namely total grains per panicle (GP), fertile grains per panicle (FGP), panicle weight (PW, g), panicle height (PH, cm), flag leaf length (FLL, cm), flag leaf width (FLW, cm), number of productive tillers (PT), thousand (1,000) grain weight (TGW, g), plant height (PH, cm) and panicle length (PL, cm). Similarly, a strong and positive genotypic correlation was observed between YLD and traits GP, TGW, PW, PH, FLL, FLW, PT, PL.

| **Supplementary Table 5**: Correlation among the agro-morphological traits in 24 selected recombinant inbred lines (RIL) | | | | | | | | | | | |
| --- | --- | --- | --- | --- | --- | --- | --- | --- | --- | --- | --- |
|  | DFF | GP | FGP | TGW | PW | PH | FLL | FLW | PT | PL | BM |
| DFF |  |  |  |  |  |  |  |  |  |  |  |
| GP | -0.08 |  |  |  |  |  |  |  |  |  |  |
| FGP | -0.09 | 0.74** |  |  |  |  |  |  |  |  |  |
| TGW | -0.07 | 0.23** | 0.13** |  |  |  |  |  |  |  |  |
| PW | -0.40 | 0.02 | 0.06 | 0.41* |  |  |  |  |  |  |  |
| PH | -0.19 | -0.25 | -0.23 | 0.50* | 0.37 |  |  |  |  |  |  |
| FLL | 0.05 | -0.32 | -0.29 | 0.13 | 0.42* | 0.58** |  |  |  |  |  |
| FLW | 0.04 | 0.10 | -0.01 | 0.24 | 0.50* | 0.43* | 0.30 |  |  |  |  |
| PT | -0.48* | 0.05* | 0.06** | 0.37 | 0.46* | 0.25 | 0.20 | -0.05 |  |  |  |
| PL | -0.49* | -0.06 | -0.09 | 0.43* | 0.81** | 0.60** | 0.54** | 0.57** | 0.41* |  |  |
| BM | 0.33 | -0.08 | -0.04 | -0.01 | -0.31 | -0.14 | -0.31 | 0.08 | -0.13 | -0.41* |  |
| YLD | -0.51* | 0.27** | 0.33** | 0.43* | 0.83** | 0.39 | 0.38* | 0.56** | 0.39** | 0.88** | -0.37 |

DFF-Days to 50% percent flowering; YLD-Total grain yield/plant(g); GP-Total Grains/panicle; FGP-Fertile grains/panicle; TGW-Test (1000) grain weight (g); PW-Panicle weight (g); PH-Plant height (cm); FLL-Flag leaf length (cm); FLW-Flag leaf width (cm); PT-Number of productive tillers; PL-Panicle length; BM-Biomass. * significant at 5% level of significance, ** significant at 1% level of significance

**Supplementary Table 6:**  Sequences of SSR primers used in the study

| S.No | SSR Marker name | Chr no. | Expected amplicon size (bp) | Observed amplicon size (bp) | Chr. Posn. (Mb) | Forward Primer's Sequence | Reverse Primer's Sequence |
| --- | --- | --- | --- | --- | --- | --- | --- |
| 1 | RM10009 | 1 | 290 | 270 | 0.18 | GATGCTCCGGAATAACTAGATTGG | GGAATTACAGCTGTCTTGGAAGG |
| 2 | RM495 | 1 | 178 | 160 | 0.21 | ATGATGATGGACGACGACAACG | TGAATCCAAGGTGCAGAGATGG |
| 3 | RM3252 | 1 | 163 | 180 | 0.3 | ATGCAAGCATCTGCTTATGG | GTTGGTAACTTTGTTCCCATGC |
| 4 | RM4554 | 1 | 142 | 200 | 0.38 | CCGTACCAAACACCAACACTGC | CGGCCGATCATCTAATCTAATCTACC |
| 5 | RM4959 | 1 | 388 | 350 | 0.45 | GATCGGATCCTTCCAAATTGC | TATTTCCTCGCTAGCTCCAACC |
| 6 | WR1.1-1 | 1 | 300 | 250 | 1.1 | CGGTTTCAGAGCGTTTTTAG | AGGAAATAAAGCTTCGGTAG |
| 7 | RM10115 | 1 | 246 | 225 | 2.15 | ACAAGACGAGGTAACACGCAAGC | GCGAAGGATCAACGATGATATGG |
| 8 | RM6324 | 1 | 147 | 140 | 2.37 | CTGTACAAGAACGGCAGCAACC | GCACCACCAAACAGAGACAGAGG |
| 9 | RM10167 | 1 | 253 | 200 | 3.39 | GTGCCTAAGATTCAGCGCTGTGG | GCGGGACATTCATGTACACATTGC |
| 10 | RM10189 | 1 | 286 | 200 | 3.76 | CCCAGCGACTGACATCGAACC | TTATCTTACACGGCTCTGTGATGTGC |
| 11 | RM3604 | 1 | 167 | 175 | 5.13 | CAGGAACCAACCTTCTTCTTGACC | GTCAGACTCCGATCTGGGATGG |
| 12 | RM312 | 1 | 295 | 200 | 10.98 | GTATGCATATTTGATAAGAG | AAGTCACCGAGTTTACCTTC |
| 13 | RM493 | 1 | 178 | 220 | 12.26 | GTACGTAAACGCGGAAGGTGACG | CGACGTACGAGATGCCGATCC |
| 14 | RM12434 | 2 | 188 | 450 | 2.07 | TTGGTCATCTTTGGTTGGTTCAGG | GGACAATTCACACAGCAGAATTGG |
| 15 | RM12725 | 2 | 240 | 200 | 6.7 | GCCTATAGCCACATTATTGC | ACAACTCCTTCATGTGATGC |
| 16 | RM13630 | 2 | 96 | 100 | 25.14 | GCCGCTGATCTCTTACCTGTTACGG | GTGCTTCGGCACAGGTCAATGC |
| 17 | RM263 | 2 | 235 | 210 | 25.88 | AATCTATGGACCTGGGAGGAACC | TGACGAGAGTGCTACGTTTGAGC |
| 18 | RM13709 | 2 | 145 | 145 | 26.5 | GTGGCTTGATTTCTGCAACTTCC | GCTGGTACCTACCAAGTATCATTTCG |
| 19 | RM517 | 3 | 190 | 175 | 6.13 | CAGCTCCTTCCTATCCGTCTCC | TCAGATCTAGCCGAGAAATCAAGG |
| 20 | RM15326 | 3 | 249 | 260 | 20.6 | TGAATCTACCGCTCTACTTGTGG | AAACAGTGCATCCTTCTTGTGG |
| 21 | JGT03-26.8 | 3 | 227 | 250 | 26.8 | GAGCGTTTGTAGTAAGTTTCATGGAC | GGCCCAACCCAAACACAAAT |
| 22 | RM15679 | 3 | 392 | 240 | 26.87 | TAGATGTATGAGTCGGAATGGAGTCG | CAGACGCAGTGTGTGTATGAAGTTCC |
| 23 | RM422 | 3 | 400 | 390 | 33.5 | TGCTATACCCAAATGTCCAAGC | ATCTTCTGAGTGCTGCCAAGC |
| 24 | RM448 | 3 | 158 | 200 | 34.49 | TCTGATCTTGATGCAGGAC | TCTCCCGATTTGGACAGATC |
| 25 | RM16238 | 3 | 197 | 200 | 36.06 | GCAGCGCATCATTGTATTAAGG | GGACACTAAATCAGAAACCCATGC |
| 26 | RM16606 | 4 | 276 | 280 | 11.62 | TGCACTTCTTTAGAGTAGGAGGAAGC | CATGCATGTGTCCAAAGATTCG |
| 27 | RM16868 | 4 | 199 | 200 | 19.47 | ATATTAGAGCATCTCCGACAGC | AATCTTGGAGCCCATACTCC |
| 28 | RM2565 | 4 | 152 | 180 | 20.93 | CCTGTGTGCATAGGATATGTTGC | TTATTTGCACGGATGGAACC |
| 29 | RM16973 | 4 | 379 | 380 | 20.94 | CACGTTAGCCGTACATGTGTATTCC | GGTCCTGGATAAAGCCTCAGTAGC |
| 30 | RM3524 | 4 | 251 | 200 | 22.86 | CTGTCTCCGTCTTCCTCACTCG | TGGAGAAATCTCCCTTCCTGAGC |
| 31 | RM17162 | 4 | 330 | 330 | 24.44 | GATGTACCAGTCCAGTTACAAAGACC | CCTTCAGAGTCTGCACACAGG |
| 32 | RM1388 | 4 | 190 | 280 | 25.19 | GATCGTTGCTGCTTTCAATGAGG | AGTGTTTCACCTTGGACCCATGC |
| Supplementary Table 6 (continued) | | | | | | | |
| S.No | SSR Marker name | Chr. no. | Expected amplicon size (bp) | Observed amplicon size (bp) | Chr. Posn. (Mb) | Forward Primer's Sequence | Reverse Primer's Sequence |
| 33 | RM252 | 4 | 99 | 220 | 24.02 | TTCGCTGACGTGATAGGTTG | ATGACTTGATCCCGAGAACG |
| 34 | RM6748 | 4 | 255 | 255 | 30.9 | AGAGAAGCAGCTGGTGATTAGCC | CAACGATGTACCAGTTGAATACCC |
| 35 | RM5511 | 4 | 250 | 240 | 31.14 | GAGTTCGTCCCTGACAAACAGAAACG | GTGAGCGAGCGAGTGAGTGAGC |
| 36 | RM6909 | 4 | 361 | 400 | 32.09 | TTTGGGATAAATGGGAGAGG | TCAGCTGGTTATTATGGAGAGG |
| 37 | RM18414 | 5 | 490 | 180 | 15.65 | CAATCCGGGACTAAAGATCG | TTCTGAGCTAGCGACTACTACTACG |
| 38 | RM18516 | 5 | 189 | 200 | 17.35 | CTTTCGTCCTGTACGTAAACC | TCAAACTACCCTCACATTCTCC |
| 39 | RM586 | 6 | 295 | 253 | 1.47 | TGCCATCTCATAAACCCACTAACC | CTGAGATACGCCAACGAGATACC |
| 40 | RM19410 | 6 | 208 | 150 | 2.91 | TGCTGATTGCTCACTACTTCATCC | GCGGGATACCATGGTCTAAAGG |
| 41 | RM19429 | 6 | 196 | 200 | 3.41 | TATGTGGTTGGCTTGCCTAGTGG | TGCCCATATGGTCTGGATGTGC |
| 42 | RM276 | 6 | 141 | 150 | 6.24 | GTCCTCCATCGAGCAGTATCAGC | CTAGCAAGACATGGACCTCAACG |
| 43 | RM19660 | 6 | 348 | 400 | 6.74 | TTTGTCCCTGCCGTACTTGC | AGCCACGTTGGGTGAAATTAGC |
| 44 | JGT06-6.81 | 6 | ------- | 400 | 6.81 | GAGCGTTTGTAGGAAGTTTCATGGAT | GGACAACCACAAGCACACCACTCT |
| 45 | JGT06-6.9 | 6 | ------- | 350 | 6.9 | TGGAGAGGAGTGGATGCGTACCTA | TCTATTGGAGATGAACCAACCACC |
| 46 | RM7023 | 6 | 392 | 185 | 6.98 | GACAGATCTTGGGCATTTGTTAGC | TGTACACTTGCATGCTTCTCTACAGG |
| 47 | ESSR06-7.1 | 6 | ------- | 130 | 7.10 | ACACCCTGACACCTCCACTC | GGTGGAAGCCATGGATGA |
| 48 | Chr 6-9.0 | 6 | 313 | 400 | 9.00 | AACCAATGCACACTTATCTGTGC | CAACTGTAGAGGTTGGAATGATCT |
| 49 | RM20098 | 6 | 212 | 200 | 17.22 | GGATACGCGATACGAGGATACG | TCAGCGTGAATTAGCTACAAGAGG |
| 50 | Chr 6-21.4 | 6 | 389 | 400 | 21.4 | ACAAGGGCAACTCACTGACC | ACGATAGCAATACCAGTTCAGG |
| 51 | RM20265 | 6 | 389 | 155 | 21.49 | ACAAGGGCAACTCACTGACC | ACGATAGCAATACCAGTTCAGG |
| 52 | RM20775 | 7 | 182 | 175 | 0.05 | GGTGTTCATCTTTGTTGAGTCAGG | AGGATACAGCGCACCAGATTAGC |
| 53 | RM20818 | 7 | 375 | 175 | 0.45 | AGATGCAGATAGATGCATGTCACG | ACCGATCATCCACGATCCTACG |
| 54 | RM6697 | 7 | 307 | 430 | 1.18 | TATTCCCGGGAGATCCAACAGC | AAGATCCAGTCGATTTGGTTCAGG |
| 55 | Chr 7-1.2 | 7 | 254 | 260 | 1.20 | GCTAGTAAGAGTGTGCAGTCATGG | CATGTGCCTCTTCTGAGTACTGG |
| 56 | RM20948 | 7 | 317 | 370 | 2.52 | GCAAGCTGGAAGAACATCGTACC | TGCTTATGGTTCTGGTCACTTCG |
| 57 | RM21103 | 7 | 99 | 100 | 4.59 | ACAAGTCCACAAGGACCACAACC | TGCTCCACCCAAAGATACAGAGC |
| 58 | RM21349 | 7 | 486 | 400 | 9.90 | AACACAATTCACCGGTCCTTAGC | GATCGATCTTTAGTCCCGGTTGG |
| 59 | RM21392 | 7 | 189 | 180 | 11.04 | GCAAGTGAGAGTTAACAAGTGG | GATCAGGTGACATACACATCG |
| 60 | RM21539 | 7 | 395 | 320 | 16.44 | GCCCAACTACTTCGACAGCTTCC | CAATGACCTGAGTAGCATCCAAGG |
| 61 | RM21565 | 7 | 395 | 250 | 17.04 | TATAGGTGGTAGCAACGCTGACG | GGTACCTCTCAAGCCAACAAGTATCC |
| 62 | RM21649 | 7 | 322 | 320 | 18.81 | CCCTGTCTAAGTCTGTTTGACG | GCCGGTTTATATTCTTGACTGG |
| 63 | RM21693 | 7 | 463 | 380 | 19.45 | GCACAGACCAGAACTTTCTTCG | TGGCGAGTGTAGATGTAATTGG |
| 64 | RM21842 | 7 | 266 | 300 | 22.72 | GAACGGGAGGAGGAGTTGTAGG | GACTTCATTTCAACTCGACGATGG |
| Supplementary Table 6 (continued) | | | | | | | |
| 65 | RM1132 | 7 | 88 | 100 | 23.93 | TCAAGGTCGACATGTTAGGTATGC | AACCCTATCACCTGAGAAACATCC |
| 66 | RM21941 | 7 | 208 | 200 | 24.72 | TACCTATTGGATGGTGGATCAGTGG | TGGGACGGAACGTGTACTACTCC |
| 67 | JGT07-25.2 | 7 | --------- | 200 | 25.2 | AGCTGTTGTGCATGATGTTCGTA | GGCGAGGGGTACAAGGACTT |
| 68 | RM21975 | 7 | 239 | 240 | 25.48 | GCCATGAGGTAGGAAATTCATCG | ACTAGTACACTGCAGATCACGTAGGG |
| 69 | RM21976 | 7 | 195 | 210 | 25.53 | CTTCCTCCTACCTTCCTCCATCC | GCACCATCACCTCCATCTCTAGC |
| 70 | RM21992 | 7 | 296 | 200 | 25.8 | ATGGGTGAGGCTTTAAACACAGG | TAAGCCAGTCCATTGGCAAACC |
| 71 | RM22171 | 7 | 392 | 205 | 29.43 | TAGTACCGCCATTACCATTCATCC | GACGGTGGGACTCCTAATTACAGC |
| 72 | RM6925 | 8 | 298 | 150 | 0.64 | GAATGAGAGGACGCTTGAAGAGG | GCATTCAGTCCCAGCTTGTATCG |
| 73 | RM22266 | 8 | 286 | 175 | 0.76 | GGGAGAAGCTGTAGCAGTTAGAAGC | CAAATGCAGAAGCCATTGTCC |
| 74 | RM22502 | 8 | 272 | 225 | 4.63 | AATCTTCACGCACCCATTCC | GCAGATACACACATTGCATACCC |
| 75 | RM22523 | 8 | 438 | 440 | 5.1 | CGTGCTTAATTACCTGGCTTCG | CGTGCTTAATTACCTGGCTTCG |
| 76 | RM22554 | 8 | 283 | 175 | 5.58 | TTGTCAAGATCATCCTCGTAGC | GTCATTCTGCAACCTGAGATCC |
| 77 | RM22565 | 8 | 277 | 280 | 5.79 | TCCACGCGTTGTCGTAGAAATTTAGC | AGCCCGAGCACCATGAAACACC |
| 78 | RM22622 | 8 | 277 | 200 | 7.08 | TAGGCCGTTCTGACGTAATACCC | CAGTGATGGTGATGCGATTTAGC |
| 79 | RM22732 | 8 | 616 | 500 | 9.88 | TCTTTGAAGGTCATTCCTGGAACC | CGCCCTTAGCTGTGTTATTGTAATCG |
| 80 | RM1384 | 8 | 360 | 360 | 11.84 | TGGTACGGGAGAACTGGTACGC | AATCGAGCCAGCCTAGCAAGC |
| 81 | RM22837 | 8 | 277 | 260 | 12.36 | ACCTGGGTCAGATGTCTGTTTGG | GGTAGAGCTCCATCCATCTTAGTGC |
| 82 | SSR-8-13 | 8 | 199 | 150 | 13 | TGCTTGTTCTTCCTCCCAAGC | CTTGCCCGTATTGTGGTCTCG |
| 83 | RM22918 | 8 | 411 | 200 | 15.58 | ACTCCAACTGTAGTGTTCTGTGC | CCATTTGTCAGTGTGTTGATCC |
| 84 | SSR-8-18 | 8 | ------ | 400 | 18 | GGTGAATTCTACTACGACGCATCG | GGTGCTGCTCATGACAATTAAGG |
| 85 | RM23029 | 8 | 345 | 200 | 18.51 | GGTGAATTCTACTACGACGGATCG | GGTCGTGCTCATGACAATTAAGG |
| 86 | RM23310 | 8 | 363 | 310 | 23.54 | GATGAGGCCCAGATTTACATTTCC | CTAAGACTTCCTGACGATGTCAATGC |
| 87 | RM23350 | 8 | 347 | 380 | 24.18 | ACAGCATGCATAGAGAAGAGTTGG | AGATCAGGACCACATGACAATGC |
| 88 | RM23736 | 9 | 199 | 200 | 2.41 | GGCGATACCTGCCATAGTTTCC | CCGAAAGCAATCTATGAGACACC |
| 89 | RM23791 | 9 | 329 | 390 | 4.26 | TCACCAACAAGTGGAGTACTTAGGC | CCTTACCTCAGGAGTGTTCGATCC |
| 90 | RM23959 | 9 | 144 | 140 | 7.94 | TGCCAAAGCTAGCTACACACTCC | TGCATCATCTTCATCTCTCCATCC |
| 91 | RM23958 | 9 | 89 | 150 | 7.94 | CTACCACTGTTTCATTGTGTCTCG | GAATTGAAGGAGAAGCAGGAAGC |
| 92 | RM24217 | 9 | 458 | 190 | 13.04 | CAGAATCCAATAGGCTCCACACG | GTCAACGGCCACTTCAAGCTACC |
| 93 | RM105 | 9 | 134 | 130 | 12.55 | GTCGTCGACCCATCGGAGCCAC | TGGTCGAGGTGGGGATCGGGTC |
| 94 | RM566 | 9 | 143 | 240 | 14.65 | AATATGGTGGCGCGTACATCC | TGATCGAGCCAACAACAACTGG |
| 95 | SSR9-17.2 | 9 | 193 | 178 | 17.2 | GGCCCAGCACTTAATAGACATCG | ACAGCGAACGTGGTGTCTCC |
| 96 | RM107 | 9 | 179 | 189 | 19.77 | TCTTACTGCGTCCTCTGGGTTCC | ATTCTTGCGGCGATTCATCTTCC |
| 97 | RM24718 | 9 | 158 | 200 | 20.9 | TGACGTGGCAAGTTGACTGTGG | TAGCCGATGGAGCCACTAGAAGG |
| 98 | RM24780 | 9 | 158 | 190 | 21.77 | GACTAGCCAGCCAAGGTTTGAGC | TGCTGCATGTGTGTATGTGACTACG |
| 99 | RM25101 | 10 | 148 | 150 | 5.37 | ATCTTGGAGCCCATACTCTATGTAGC | GTGGATGGGAGCAGGAGTACG |
| Supplementary Table 6 (continued) | | | | | | | |
| 100 | RM25147 | 10 | 295 | 350 | 7.15 | GACTTAAGTTCTCCTTGGTCTTGG | CCTGATTCACTTGATTCACTCG |
| 101 | Chr 10-17.9 | 10 | 286 | 255 | 17.9 | CCACATTAACTGAAGAGACCATGC | TTGCCCTCATCCTGTAATATGTCC |
| 102 | RM25735 | 10 | 387 | 380 | 19.56 | AGGCAGGCAAGCAGTAGTTTCG | ATCAAGATCAGGAGCCGCAAGG |
| 103 | RM25754 | 10 | 326 | 290 | 19.75 | TTTCATTCTACCACCCAGGTTGC | GGACCTCTTCTTTCTCATGTTCTGC |
| 104 | RM286 | 11 | 235 | 110 | 0.38 | CTGGCCTCTAGCTACAACCTTGC | AAACTCTCGCTGGATTCGATAGG |
| 105 | Chr 11-0.9 | 11 | 147 | 140 | 0.9 | CGATTGATCCCGTGCAAGTAGG | CATGCTAGTGCATTCTGCGTAGG |
| 106 | RM7203 | 11 | 392 | 120 | 1.08 | CATCTCTGGCCTTCTACTCATGG | TGGAAATTTCTAGCCTCCACAGC |
| 107 | JGT11-16.3 | 11 | ------- | 150 | 16.3 | GGCGGCGTATTAGCGTTGTA | AGGTTCTAGCCCATGTTAAATCTTCT |
| 108 | Chr 11-20.5 | 11 | 298 | 400 | 20.50 | AGAGGATGAACTACAGGGCAAGC | GTGAGGTGGGAACTAATCCATCG |
| 109 | RM209 | 11 | 134 | 170 | 20.55 | ATATGAGTTGCTGTCGTGCG | CAACTTGCATCCTCCCCTCC |
| 110 | RM21 | 11 | 157 | 160 | 22.04 | ACAGTATTCCGTAGGCACGG | GCTCCATGAGGGTGGTAGAG |
| 111 | RM27096 | 11 | 260 | 180 | 23.47 | AGTTAGGATCGCTTCCAGGTTCC | TCCAACTGGAATATCGTCTTGTAGGC |
| 112 | RM224 | 11 | 157 | 125 | 30.45 | ATCGATCGATCTTCACGAGG | TGCTATAAAAGGCATTCGGG |
| 113 | Chr 11-28.1 | 11 | 233 | 200 | 28.1 | ACATATCGACGGTGGATGAGAGC | TCCGTGTGCATACATTCTTGAGC |
| 114 | RM27404 | 12 | 200 | 200 | 0.20 | GTTGCATGGCAACGAGGAGAGG | GTTACGACCGTGCCATCTTGTCC |
| 115 | RM101 | 12 | 154 | 120 | 8.82 | AAGTAGTGGTCGAAGTGTGTATCG | GGTGAATGGTCAAGTGACTTAGG |
| 116 | RM27920 | 12 | 267 | 400 | 10.14 | AAAGCGAGAAATCCGGAGATGG | TCCTCTCTCAAATCTCCTCGAAGC |
| 117 | RM27966 | 12 | 263 | 250 | 12.16 | TCTGAGCCAACAGTAAGAGTCAGG | TGTCACCCGTAGTGTTTGTACGG |
| 118 | RM28085 | 12 | 388 | 380 | 15.38 | CCCGCTGCAGCAGTTTATTGAGG | GATCTGGTACCTGCATGGGTTGC |
| 119 | RM511 | 12 | 143 | 140 | 17.44 | AACGAAAGCGAAGCTGTCTCC | ATTTGTTCCCTTCCTTCGATCC |
| 120 | JGT12-18.6 | 12 | 222 | 200 | 18.6 | ATGGCAGCGTAGGAGCGTTTGTAG | AACGTACCTAGGCAGCAAAATTCT |
| 121 | RM28275 | 12 | 231 | 230 | 19.47 | ATTCCAACCCAACCTCCAAATTCC | GTTGGGCTGTACTGTACGTCTTGACC |
| 122 | RM28277 | 12 | 297 | 260 | 19.49 | TGCACCACCTATTTCAATCCACTCC | CCTTCCTCAAGGGAAATCACAGAAGC |
| 123 | RM519 | 12 | 125 | 125 | 19.97 | AATTTCCGCGAAATCAGCATCC | TCATCTGGACAGTCGAGGTACGC |
| 124 | ESSR12-20.2 | 12 | ------- | 200 | 20.2 | GGTGTTGCAGGCGTCCTACT | TCATGGAATGGAAACAACCA |
| 125 | RM5479 | 12 | 292 | 400 | 24.44 | CTCACCATAGCAATCTCCTGTGC | ACTTCGTTCACTTGCATCATGG |
| 126 | RM235 | 12 | 157 | 160 | 26.17 | AAGCTAGGGCTAACGAACGAACG | TCTCCATCTCCATCTCCATCTCC |

Out of 126 markers used for the study, 92 markers (72.44%) of them were bi-allelic, 26 were tri-allelic (20.47%) and 9 (7.08%) were tetra-allelic

**Supplementary Table 7:** Putative minor effect QTLs for agronomic traits in 105recombinant inbred line (RIL) population derived from the cross of IR58025A × KMR-3R (KRH-2) identified with 126 SSR markers

| Trait^a^ | QTL^b^ | Flanking markers | | Chr | Start position (cM) | End position (cM) | QTL size (cM) | LOD peak^c^ | PVE(%)^d^ | Add^e^ | RSq^f^ |
| --- | --- | --- | --- | --- | --- | --- | --- | --- | --- | --- | --- |
| DFF | *qDFF9-1* | RM23791 | RM24718 | 9 | 208.09 | 265.15 | 56.25 | 2.84 | 6.10 | -1.82 | 22.57 |
|  | *qDFF12-1* | ESSR12-20.2 | RM5479 | 12 | 106.1 | 101.83 | 4.27 | 7.36 | 19.77 | 5.87 | ----- |
| YLD | *qYLD6-1** | RM7023 | ESSR06-7.1 | 6 | 0.00 | 26.47 | 26.47 | 5.55 | 12.35 | 2.46 | 32.92 |
| GP | *qGP8-1*^#^ | RM1384 | SSR-8-13 | 8 | 140.00 | 153.39 | 13.39 | 4.08 | 6.41 | -14.82 | 14.26 |
|  | *qGP12-1* | RM101 | RM27966 | 12 | 109.13 | 118.62 | 9.49 | 3.72 | 13.96 | -13.98 | ----- |
| FGP | *qFGP8-1* | RM22554 | RM1384 | 8 | 132.91 | 140.00 | 7.09 | 3.13 | 14.54 | -14.48 | 11.76 |
| TGW | *qTGW5-1* **^Ɨ^** | RM18414 | RM18516 | 5 | 0.00 | 27.97 | 27.97 | 3.07 | 13.02 | -1.65 | 30.02 |
|  | *qTGW8-1* | SSR-8-18 | RM22837 | 8 | 162.04 | 163.57 | 1.53 | 9.09 | 16.78 | 1.73 | ---- |
|  | *qTGW12-1* | RM28275 | RM28085 | 12 | 32.8 | 69.05 | 36.25 | 2.80 | 10.70 | -0.96 | ---- |
| PW  PH | *qPW6-1** | RM7023 | ESSR06-7.1 | 6 | 0.00 | 26.47 | 26.47 | 6.17 | 13.86 | 2.30 | 35.01 |
|  | *qPW8-1*^#^ | RM1384 | SSR-8-13 | 8 | 140.00 | 153.39 | 13.39 | 2.88 | 6.14 | -1.75 | ---- |
|  | *qPH12-2* | RM27966 | RM235 | 12 | 118.62 | 130.53 | 11.91 | 3.73 | 9.41 | -5.41 | 30.36 |
| FLW | *qFLW4-2* | RM252 | RM6748 | 4 | 69.94 | 55.79 | 14.15 | 6.50 | 19.39 | 0.13 | 18.26 |
| PL | *qPL6-1** | RM7023 | ESSR06-7.1 | 6 | 0.00 | 26.47 | 26.47 | 4.55 | 10.72 | 2.16 | 31.57 |
|  | *qPL9-1* | RM23958 | RM23959 | 9 | 43.37 | 70.29 | 26.92 | 2.53 | 7.96 | -1.89 | ------ |
| BM | *qBM5-1* **^Ɨ^** | RM18414 | RM18516 | 5 | 0.00 | 27.97 | 27.97 | 3.31 | 7.47 | 8.39 | 0 |
|  | *qBM11-1* | RM286 | RM27096 | 11 | 109.77 | 131.87 | 22.1 | 2.76 | 5.08 | -6.13 | ------ |

^a^DFF-Days to 50% percent flowering; YLD-Total grain yield/plant(g); GP-Total grains/panicle; FGP-Fertile grains/panicle; TGW-Test (1000) grain weight (g); PW-Panicle weight (g); PH-Plant height (cm); FLW-Flag leaf width (cm); PL-Panicle length (cm); BM-Biomass (g)

^b^Designation of the QTLs is in accordance with the rules recommended by^60^

^c^Logarithm of the odds ratio (LOD) score of ≥2.5 was set as threshold for this data

^d^Total phenotypic variance (PVE%) in percentage explained by the QTL

^e^Additive effect-Negative additive effect value indicates the direction of favorable allele from donor parent, KMR-3R, that increased the trait value

^f^Phenotypic variation explained by the final regression model

*QTL hotspot on chr 6 for total grain yield/plant (g), panicle weight (g) and panicle length(cm)

^#^QTL hotspot on chr 8 for total grains/panicle, panicle weight (g)

^Ɨ^QTL hotspot on chr 5 for test (1000) grain weight (g) and biomass (g)

Putative identified QTLs for yield and it’s related agronomic traits in 105 recombinant inbred line (RIL) population. Those QTLs whose LOD scores was greater than 2.5 and whose phenotypic variance percentage (PVE%) was greater than 20%, were identified as major effect QTLs. QTL hotspots on chr 5, 6 and 8 were also identified.

**Supplementary Table 8**: Putative minor effect QTLs for agronomic traits in 105 recombinant inbred line (RIL) population derived from the cross of IR58025A × KMR-3R (KRH-2) and those identified from previous studies

| Trait^a^ | QTL^b^ | Flanking markers | | Chr. | QTL position(s) in this study  (cM/Mb) | Published QTLs names in previous study | Flanking marker(s) in previous study | | | Published QTL position(s) in previous study  (cM/Mb) | Previous study (Reference number) |
| --- | --- | --- | --- | --- | --- | --- | --- | --- | --- | --- | --- |
| DFF | *qDFF9-1* | RM23791 | RM24718 | 9 | 4.26-20.9 Mb | *qDFF9.1* | RM566 | RM434 | | 14.7-15.6Mb | 112 |
|  | *qDFF12-1* | ESSR12-20.2 | RM5479 | 12 | 20.2-24.44 Mb | qDF_12.1_ | id12006721 | 12884952 | | 20 Mb | 85 |
| YLD | *qYLD6-1* | RM7023 | ESSR06-7.1 | 6 | 0-26.47 cM | *qGYD-6-1* | RZ398 | RM204 | | 15-25.1 cM | 113 |
|  |  |  |  |  | 6.98-7.1 Mb | qYLD_6.1_ | 5895767 | 5901730 | | 0-5 Mb | 85 |
| GP | *qGP8-1* | RM1384 | SSR-8-13 | 8 | 11.83-13 Mb | *qSNP-8* | RM483 | RM137 | | 21.3-1.1 Mb | 114 |
|  | *qGP12-1* | RM101 | RM27966 | 12 | 8.82-12.16 Mb | qSNP-12 | RM7626 | RM1337 | | 12.04 Mb | 88 |
| FGP | *qFGP8-1* | RM22554 | RM1384 | 8 | 5.58-11.84 Mb | *nfg8.1* | RM38 | RM223 | | 2.1-20.65 Mb | 92 |
| TGW | *qTGW5-1* | RM18414 | RM18516 | 5 | 0-27.97 cM | *tgwt5b* | -RG573- | | | 13.6-19.7 cM | 115 |
|  |  |  |  |  | 0-27.97 cM | ---------- | RM122 | | | 0 cM | 82 |
|  | *qTGW8-1* | SSR-8-18 | RM22837 | 8 | 18-12.36 Mb | *hgw8* | RM223 | | | 20.65 Mb | 116 |
|  |  |  |  |  |  | *qtgw8* | RM502 | | RM264 | 26.4-29.3 Mb | 61 |
|  | *qTGW12-1* | RM28275 | RM28085 | 12 | 19.47-15.38 Mb | *qTGW12* | RM28597 | | RM17 | 24.5-25.9 Mb | 117 |
| PW  PH | *qPW6-1* | RM7023 | ESSR06-7.1 | 6 | 0-26.47 cM | ---------- | ---------- | | ---------- | ---------- | ---------- |
|  | *qPW8-1* | RM1384 | SSR-8-13 | 8 | 140.00-153.39 cM | ---------- | ---------- | | ---------- | ---------- | ---------- |
|  | *qPH12-2* | RM27966 | RM235 | 12 | 12.16-26.17Mb | *qtl12.1* | RM28048 | | RM28166 | 14.1-17.6 Mb | 90 |
| FLW | *qFLW4-2* | RM252 | RM6748 | 4 | 30.99-32.71 Mb | QFlw4 | RM255 | | RM349 | 24.0-30.9 Mb | 89 |
| PL | *qPL6-1* | RM7023 | ESSR06-7.1 | 6 | 5.2–7.9 Mb | *qPL6.1* | YJInDel-207 | | YJInDel-208 | 6.98-7.1 Mb | 118 |
|  | *qPL9-1* | RM23958 | RM23959 | 9 | 43.37-70.29 cM | *qPL9* | RM5632 | | H90 | 72.21 cM | 119 |
| BM | *qBM5-1* | RM18414 | RM18516 | 5 | 15.65-17.35 Mb | qBI-5 | RM440 | | RM5140 | 19.8-13.4 Mb | 88 |
|  | *qBM11-1* | RM286 | RM27096 | 11 | 0.38-23.47 Mb | *bm11.1* | RM209 | | RM21 | 17.8-22 Mb | 92 |

^a^DFF-Days to 50% percent flowering; YLD-Total grain yield/plant(g); TGW-Test (1000) grain weight (g); GP-Total grains/panicle; FGP-Fertile grains/ panicle ; PW-Panicle weight (g); PH-Plant height (cm); FLL-Flag leaf length (cm); FLW-Flag leaf width (cm); PL-Panicle length (cm); BM-Biomass (g)

^b^Designation of the QTLs is in accordance with the rules recommended by McCouch and CGSNL (McCouch 2008)

| Trait Name^a^ | First  Chr. | Position 1 (cM) | LeftMarker1 | RightMarker1 | Second  Chr. | Position2 (cM) | LeftMarker2 | RightMarker2 | LOD  peak^b^ | PVE(%)^c^ | AddbyAdd^d^ | RSq^e^ |
| --- | --- | --- | --- | --- | --- | --- | --- | --- | --- | --- | --- | --- |
| YLD | 3 | 130 | RM517 | RM15679 | 7 | 95 | RM21992 | RM22171 | 7.38 | 20.16 | -5.36 | 32.90 |
| PL | 3 | 0 | RM448 | RM422 | 6 | 5 | RM7023 | ESSR06-7.1 | 5.84 | 20.38 | 2.26 | 31.57 |
| PL | 3 | 125 | RM517 | RM15679 | 7 | 95 | RM21992 | RM22171 | 6.98 | 29.73 | -4.04 | ----- |

**Supplementary Table 9:**  Major effect epistatic interactions between QTLs in 105recombinant inbred lines (RILs)

^a^YLD-Total grain yield/plant(g); PL-Panicle length (cm)

^b^Logarithm of the odds ratio (LOD) score of ≥2.5 was set as threshold for this data.

^c^Total phenotypic variance in percentage explained by the QTL;

^d^Additive by additive effect-Negative additive effect value indicates the direction of favorable allele from donor parent, KMR-3R, that increased the trait value

^e^Phenotypic variation explained by the final regression model.

Epistatic interactions with LOD peak more than 2.5 and PVE% more than 20% were termed as significant interactions. Significant epistatic interactions were observed between the QTLs located on chromosome 3 and chromosome 7 for the trait total grain yield/plant (g) (YLD). This interaction may account for 32.92% of the total phenotypic variance (RSq). Similarly, for the trait the panicle length (PL), significant QTL epistatic interactions were observed between the QTLs located on chromosome 3 and chromosome 6, 7 with total phenotypic variance (RSq) accounting for 31.57%.

| **Supplementary Table 10:** QTL × E interaction table in IR58025A × KMR-3R derived RIL population across three seasons (Kh’16, Rb’17, Kh’17) | | | | | | | | | | | | | |
| --- | --- | --- | --- | --- | --- | --- | --- | --- | --- | --- | --- | --- | --- |
| Major effect QTLs | | | | | | | | | | | RSq | | |
| S.No | Trait name | QTL name | Chr | Flanking markers | | LOD(AbyE) | PVE(AbyE) | AbyE_01 | AbyE_02 | AbyE_03 | Env 1 | Env 2 | Env 3 |
| 1 | YLD | *qYLD3-1* | 3 | RM517 | RM15679 | 0.00 | 0.00 | 0.00 | 0.00 | 0.00 | 34.04 | 34.04 | 34.04 |
| 2 | PW | *qPW3-1* | 3 | RM517 | RM15679 | 0.01 | 0.00 | -0.01 | 0.03 | -0.01 | 24.78 | 26.29 | 24.78 |
| 3 | PH | *qPH12-1* | 12 | RM27404 | RM28275 | 0.07 | 0.00 | 0.01 | -0.02 | 0.01 | 30.29 | 28.01 | 30.03 |
| 4 | FLW | *qFLW4-1* | 4 | RM6909 | RM252 | 0.00 | 0.00 | 0.00 | 0.00 | 0.00 | 17.96 | 17.96 | 17.96 |
| 5 | PL | *qPL3-1* | 3 | RM517 | RM15679 | 0.01 | 0.00 | -0.05 | 0.11 | -0.05 | 0.00 | 0.00 | 0.00 |
| Minor effect QTLs | | | | | | | | | | | RSq | | |
| S.No | Trait name | QTL name | Chr | Flanking markers | | LOD(AbyE) | PVE(AbyE) | AbyE_01 | AbyE_02 | AbyE_03 | Env 1 | Env 2 | Env 3 |
| 6 | DFF | *qDFF9-1* | 9 | RM23791 | RM24718 | 0.00 | 0.01 | 0.07 | -0.15 | 0.079 | 29.53 | 22.57 | 29.53 |
|  | | *qDFF12-1* | 12 | RM5479 | ESSR12-20.2 | 0.01 | 0.15 | -0.38 | 0.77 | -0.38 | 0.00 | 0.00 | 0.00 |
| 7 | YLD | *qYLD3-1* | 3 | RM7023 | ESSR06-7.1 | 0.00 | 0.00 | 0.00 | 0.00 | 0.00 | 0.00 | 0.00 | 0.00 |
| 8 | GP | *qGP8-1* | 8 | RM1384 | SSR-8-13 | 0.00 | 0.00 | 0.00 | 0.00 | 0.00 | 14.25 | 14.25 | 14.25 |
|  | | *qGP12-1* | 12 | RM101 | RM27966 | 0.00 | 0.00 | 0.00 | 0.00 | 0.00 | 0.00 | 0.00 | 0.00 |
| 9 | FGP | *qFGP8-1* | 8 | RM22554 | RM1384 | 0.00 | 0.00 | 0.00 | 0.00 | 0.00 | 11.75 | 11.75 | 11.75 |
| 10 | TGW | *qTGW5-1* | 5 | RM18414 | RM18516 | 0.00 | 0.00 | -0.00 | -0.00 | 0.00 | 30.64 | 29.81 | 31.2 |
|  | | *qTGW8-1* | 8 | SSR-8-18 | RM22837 | 0.00 | 0.00 | 0.00 | 0.00 | -0.00 | 0.00 | 0.00 | 0.00 |
| 11 | PW | *qPW6-1* | 6 | RM7023 | ESSR06-7.1 | 0.00 | 0.01 | -0.04 | 0.09 | -0.04 | 24.78 | 26.69 | 24.78 |
|  | | *qPW8-1* | 8 | RM1384 | SSR-8-13 | 0.00 | 0.00 | -0.04 | 0.09 | -0.04 | 0.00 | 0.00 | 0.00 |
| 12 | PH | *qPH12-2* | 12 | RM27966 | RM235 | 0.01 | 0.00 | -0.11 | 0.24 | -0.13 | 0.00 | 0.00 | 0.00 |
| 13 | FLW | *qFLW4-2* | 4 | RM252 | RM6748 | 0.01 | 0.00 | 0.00 | 0.16 | 0.09 | 0.00 | 0.00 | 0.00 |
| 14 | PL | *qPL6-1* | 6 | RM7023 | ESSR06-7.1 | 0.01 | 0.00 | 0.00 | 0.00 | 0.00 | 0.00 | 0.00 | 0.00 |
|  |  | *qPL9-1* | 9 | RM23958 | RM23959 | 0.01 | 0.00 | 0.00 | 0.00 | 0.00 | 0.00 | 0.00 | 0.00 |
| 15 | BM | *qBM5-1* | 5 | RM18414 | RM18516 | 0.00 | 0.00 | 0.00 | 0.00 | 0.00 | 30.64 | 29.81 | 31.2 |
|  | | *qBM11-1* | 5 | RM286 | RM27096 | 0.00 | 0.00 | 0.00 | 0.00 | 0.00 | 0.00 | 0.00 | 0.00 |

YLD-Total grain yield/plant(g); PW-Panicle weight (g); PH-Plant height (cm); FLW-Flag leaf width (cm); PL-Panicle length (cm); DFF-Days to 50% percent flowering; GP-Total grains/panicle; FGP-Fertile grains/panicle; TGW-Test (1,000) grain weight (g); BM-Biomass (g); LOD(AbyE)-LOD score for additive and dominance by environment effects; RSq-Phenotypic variation explained by the final regression model, PVE(AbyE)-Phenotypic variation explained by additive and dominance by environment effect at the current scanning position, AbyE_01-Additive and dominance by environment effect at the current scanning position, Env 1, Env 2, Env 3-Environments 1, 2 and 3.

**Supplementary Table 11**: Putative major/minor effect QTLs identified with 1,082 SNPs in 24 selected KRH-2 derived recombinant inbred line (RILs)

| S.No | QTL name^a^ | Major/minor effect | Chr | Physical posn (Mb) | QTL size (Mb) | | LOD^b^ | PVE^c^(%) | Add^d^ | RSq^e^ | Novel/Reported/Resolved QTL |
| --- | --- | --- | --- | --- | --- | --- | --- | --- | --- | --- | --- |
| Trait name: Total grain yield per plant (YLD) | | | | | | | | | | | |
| 1 | *qYLD2-1*^$^ | Major effect | 2 | 5.35-8.22 | 2.87 | | 10.82 | 30.75 | 8.35 | 81.6513 | Novel |
| 2 | *qYLD1-1* | Minor effect | 1 | 2.85-4.19 | 1.34 | | 4.10 | 3.43 | -6.75 | 0 | *qYLD1-1*overlapped with the QTL reported by^120^(1.66-4.88 Mb) with better resolution |
|  | | | | | | | | | | |  |
| 3 | *qYLD2-2* | Minor effect | 2 | 5.26-35.11 | 29.85 | | 3.90 | 3.93 | -5.26 | 0 | *qYLD2-2*reported by^84, 121, 116, 122, 123, 117, 124^ |
|  | | | | | | | | | | |  |
| 4 | *qYLD2-3* | Minor effect | 2 | 20.56-20.22 | 0.34 | | 2.68 | 3.95 | -5.25 | 0 | *qYLD2-3*detected with better resolution than earlier reported QTLs by^84, 121, 116, 122, 4, 123, 117, 125^ |
|  | | | | | | | | | | |  |
| 5 | *qYLD3-1* | Minor effect | 3 | 6.13-13.47 | 7.34 | | 3.91 | 3.43 | -6.75 | 0 | *qYLD3-1*was a part of QTL reported by^120^ (28.67-8.63 Mb) with better resolution |
|  | | | | | | | | | | |  |
| 6 | *qYLD4-1** | Minor effect | 4 | 7.95-6.25 | 1.7 | | 3.83 | 3.94 | -5.25 | 0 | *qYLD4-1*was resolved to 6.54 Mb by^82^ |
| 7 | *qYLD6-1* | Minor effect | 6 | 0.08-21.12 | 21.04 | | 4.61 | 3.94 | -5.25 | 0 | *qYLD6-1*was reported by^84, 81, 124, 85^ |
|  | | | | | | | | | | |  |
| 8 | *qYLD8-1* | Minor effect | 8 | 13.42-21.83 | 8.41 | | 3.24 | 3.94 | -5.25 | 0 | *qYLD8-1* was a part of QTLs reported by^125, 126^ |
| 9 | *qYLD10-1*^#^ | Minor effect | 10 | 20.69-9.62 | 11.04 | | 9.68 | 14.43 | -6.45 | 0 | *qYLD10-1*overlapped with QTLs reported by^117^(17.93-18.81 Mb) and by^113^(22.24 Mb) |
|  | | | | | | | | | | |  |
| 10 | *qYLD10-2* | Minor effect | 10 | 6.02-18.82 | 12.8 | | 3.91 | 3.43 | -6.75 | 0 | *qYLD10-2*overlapped with QTL reported by^117^(17.93-18.81 Mb) |
| Trait name: Days to fifty percent flowering | | | | | | | | | | | |
| 11 | *qDFF2-1* | Minor effect | 2 | 2.90-2.38 | 0.52 | | 3.3195 | 3.34 | -8.37 | 35.66 | Novel QTL |
| 12 | *qDFF3-1* | Minor effect | 3 | 34.78-34.76 | 0.02 | | 3.217 | 3.34 | -8.37 | 0 | *qDFF3-1*was earlier reported by^4^(34.76-35.27 Mb) |
| 13 | *qDFF3-2* | Minor effect | 3 | 34.76-35.22 | 0.46 | | 3.21 | 3.34 | -8.37 | 0 | *qDFF3-2*was earlier reported by^4^(34.76-35.27 Mb) |
| 14 | *qDFF4-1** | Minor effect | 4 | 7.95-6.25 | 1.7 | | 2.78 | 3.28 | -9.13 | 0 | Novel QTL |
| 15 | *qDFF6-1* | Minor effect | 6 | 5.85-0.07 | 5.78 | | 3.14 | 3.28 | -9.07 | 0 | *qDFF6-1*was earlier reported by^85^ at 1 Mb and by^4^ at 1.76 Mb |
|  | | | | | | | | | | |  |
| 16 | *qDFF6-2* | Minor effect | 6 | 0.07-15.81 | 15.74 | | 3.12 | 3.28 | -9.11 | 0 | *qDFF6-2*was earlier reported by^85^ at 1 Mb; by^4^ at 1.76 Mb and ^87^(7.68 Mb) |
|  | | | | | | | | | | |  |
| Supplementary Table 11 (continued) | | | | | | | | | | | |
| S.No | QTL name^a^ | Major/minor effect | Chr | Physical posn (Mb) | | QTL size (Mb) | LOD^b^ | PVE^c^(%) | Add^d^ | RSq^e^ | Novel/Reported/Resolved QTL |
| Trait name: Test grain weight (TGW) | | | | | | | | | | | |
| 17 | *qTGW11-1* | Major effect | 11 | 1.12-0.96 | | 0.16 | 3.36 | 29.47 | 2.57 | 74.56 | *qTGW11-1*overlapped with QTL *kgw11.1*reported by^91^(0.47 Mb-2.84 Mb) |
|  | | | | | | | | | | |  |
| Trait name: Panicle weight (PW) | | | | | | | | | | | |
| 18 | *qPW2-1*^$^ | Major effect | 2 | 5.35-8.22 | 2.87 | | 6.49 | 52.88 | 6.21 | 74.35 | Novel QTL, *qPW2-1* overlapped between 4.41-5.20 Mb reported by^88^ |
| 19 | *qPW10-1*^#^ | Major effect | 10 | 20.69-9.62 | 11.07 | | 5.971 | 31.77 | -4.63 | 0 | Novel QTL |
| Trait name: Panicle length (PL) | | | | | | | | | | | |
| 20 | *qPL2-1*^$^ | Major effect | 2 | 5.35-8.22 | 2.87 | | 17.81 | 61.75 | 8.06 | 96.74 | *qPL2-1* overlapped with QTL reported by^4^with better resolution |
|  | | | | | | | | | | |  |
| 21 | *qPL10-1*^#^ | Major effect | 10 | 20.69-9.62 | 11.04 | | 14.45 | 29.94 | -7.10 | 0 | Novel QTL |
| 22 | *qPL3-1* | Minor effect | 3 | 33.43-34.24 | 0.81 | | 4.58 | 2.75 | -2.90 | 0 | Novel QTL |
| Trait name: Plant height (PH) | | | | | | | | | | | |
| 23 | *qPH2-1*^$^ | Major effect | 2 | 5.35-8.22 | 2.87 | | 2.72 | 42.71 | 4.49 | 68.69 | *qPH2-1* overlapped with QTL reported by^92^(4.63-35.77 Mb) with better resolution |
|  | | | | | | | | | | |  |
| Trait name: Flag leaf length (FLL) | | | | | | | | | | | |
| 24 | *qFLL6-1* | Major effect | 6 | 0.08-0.05 | 0.03 | | 2.86 | 42.38 | 2.81 | 39.66 | Novel QTL |
| Trait name: Fertile grains per plant (FGP) | | | | | | | | | | | |
| 25 | *qFGP5-1* | Major effect | 5 | 29.80-19.21 | 10.59 | | 2.51 | 35.51 | -16.07 | 64.54 | *qFGP5-1*resolved to 20.94 Mb by^84^ |
| 26 | *qFGP5-2* | Major effect | 5 | 3.36-29.95 | 26.59 | | 2.80 | 42.22 | 16.22 | 0 | *qFGP5-2 r*esolved to 20.94 Mb by^84^ |

^a^YLD-Total grain yield/plant (g), DFF-Days to fifty percent flowering, TGW-Test (1,000) grain weight (g), PW-Panicle weight (g), PL-Panicle length (PL) (cm), PH-Plant height (cm), FLL-Flag leaf length (cm), FGP-Fertile grain per panicle

^b^Logarithm of the odds ratio (LOD) score of ≥2.5 was set as threshold for this data.

^c^Total phenotypic variance in percentage explained by the QTL;

^d^Additive by additive effect-Negative additive effect value indicates the direction of favorable allele from donor parent, KMR-3R, that increased the trait value

^e^Phenotypic variation explained by the final regression model.

$-QTL hotspot on chromosome 2 for traits total grain yield per plant, panicle weight, panicle length and plant height

*- QTL hotspot on chromosome 4 for traits total grain yield per plant, days to fifty percent flowering

#- QTL hotspot on chromosome 10 for traits total grain yield per plant, panicle weight and panicle length

**Supplementary Table 12**: List of commonly detected QTLs with 105 RILs-126 SSRs and 24 selected RILs-1,082 SNPs

| QTLs observed in 24 RILs-1,082 SNPs | | | | | | | | | QTLs observed in 105 RILs-126 SSRs | | | | | | | |
| --- | --- | --- | --- | --- | --- | --- | --- | --- | --- | --- | --- | --- | --- | --- | --- | --- |
| SNo | QTL name | Chr | Physical posn (Mb) | QTL size (Mb) | LOD^a^ | PVE(%)^b^ | Add^c^ | RSq^d^ | QTL name | Chr | Physical posn (Mb) | QTL size (Mb) | LOD^a^ | PVE(%)^b^ | Add^c^ | RSq^d^ |
| 1 | *qYLD3-1* | 3 | 6.13-13.47 | 7.34 | 3.91 | 3.43 | -6.75 | 81.65 | *qYLD3-1* | 3 | 6.13-26.87 | 20.74 | 10.59 | 22.76 | -6.44 | 32.92 |
| 2 | *qPL3-1* | 3 | 33.43-34.24 | 0.81 | 4.58 | 2.75 | -2.90 | 96.74 | *qPL3-1* | 3 | 6.13-26.87 | 20.74 | 10.47 | 22.7 | -6.14 | 31.57 |

YLD-Total grain yield/plant (g), PL-Panicle length (cm)

^a^Logarithm of the odds ratio (LOD) score of ≥2.5 was set as threshold for this data.

^b^Total phenotypic variance in percentage explained by the QTL;

^c^Additive effect-Negative additive effect value indicates the direction of favorable allele from donor parent, KMR-3R, that increased the trait value

^d^Phenotypic variation explained by the final regression model.

**Supplementary Table 13:** Major effect epistatic interactions between QTLs in 24 selected recombinant inbred lines (RILs) when mapped with 1,082 SNPs

| S.No | Trait name^a^ | Chr | Posn (cM) | Left marker 1 | Right marker 1 | Chr | Posn (cM) | Left marker 2 | Right marker 2 | LOD^b^ | PVE(%)^c^ | Add by Add^d^ | RSq(%)^e^ |
| --- | --- | --- | --- | --- | --- | --- | --- | --- | --- | --- | --- | --- | --- |
| 1 | TGW | 1 | 120 | S1_30478141 | S1_242656 | 5 | 110 | S5_29905083 | S5_29885191 | 5.17 | 25.06 | -2.47 | 61.48 |
| 2 | TGW | 5 | 70 | S5_29805372 | S5_29844731 | 5 | 110 | S5_29905083 | S5_29885191 | 5.75 | 25.52 | 2.38 | ------ |
| 3 | PW | 2 | 45 | S2_5359418 | S2_8229921 | 8 | 55 | S8_26977980 | S8_5780637 | 5.01 | 43.52 | 1.82 | 74.35 |
| 4 | PW | 4 | 75 | S4_10183781 | S4_15489934 | 12 | 40 | S12_14963329 | S12_8961198 | 5.09 | 29.36 | 3.66 | ------ |
| 5 | FLL | 6 | 10 | S6_87110 | S6_56355 | 12 | 40 | S12_14963329 | S12_8961198 | 5.34 | 38.84 | 4.68 | 39.66 |
| 6 | FLW | 5 | 145 | S5_29950375 | S5_20289439 | 12 | 10 | S12_1576092 | S12_26810779 | 6.93 | 32.73 | -0.14 | 52.86 |

^a^TGW-Test (1000) grain weight (g), PW-Panicle weight (g), FLL-Flag leaf length (cm), FLW-Flag leaf width (cm)

^b^Logarithm of the odds ratio (LOD) score of ≥2.5 was set as threshold for this data.

^c^Total phenotypic variance in percentage explained by the QTL;

^d^Additive by additive effect-Negative additive effect value indicates the direction of favorable allele from donor parent, KMR-3R, that increased the trait value

^e^Phenotypic variation explained by the final regression model.

**Supplementary Table 14**: List of putative candidate genes identified in commonly detected QTLs with 105 RILs-126 SSRs and 24 selected RILs-1,082 SNPs

| S.No | QTL name | Chr | Significantly associated SNP marker | SNP marker's position (Mb) | RAP-DB Locus ID | Physical position of candidate gene (Mb) | Biological function of the candidate gene |
| --- | --- | --- | --- | --- | --- | --- | --- |
| 1 | *qYLD3-1* | 3 | S3_13473323 | 13.47 | Os03t0263600-01 | 8.66 | Regulation of anther development and pollen wall formation |
|  | | | | | Os03t0308200-01 | 10.97 | Regulation of male fertility and seed size |
|  |  |  |  |  | Os03t0333200-01 | 12.30-12.31 | Control of reproductive growth and development |
|  |  |  |  |  |  |  |  |
| 2 | *qPL3-1* | 3 | S3_33431112 | 33.43 | Os03t0764900-01 | 31.66 | Regulation of panicle architecture |
|  | | | | | Os03t0809900-01 | 33.89 | Embryonic organogenesis and development |
|  |  |  |  |  | Os03t0815700-01 | 34.19 | Flowering time control |

YLD-Total grain yield/plant (g), PL-Panicle length (cm)

**Supplementary Table 15**: List of putative candidate genes identified in novel QTLs identified with 105 RILs-126 SSRs and 24 selected RILs-1,082 SNPs

| List of candidate genes identified in novel QTL regions detected with 105 RILs-126 SSRs | | | | | | | | | | |
| --- | --- | --- | --- | --- | --- | --- | --- | --- | --- | --- |
| S.No | QTL name | Chr | QTL region (Mb) | QTL size (Mb) | | RAP-DB Locus ID | | Physical position of candidate gene (Mb) | | Biological function of the candidate gene |
| 1 | *qYLD3-1* | 3 | 6.1-26.8 | 20.7 | | Os03t0669200-01 | | 26.39-26.40 | | Seed fertility |
| 2 | *qPW6-1* | 6 | 6.98-7.1 | 0.12 | | Os06t0234100-01 | | 6.90-6.98 | | Peptidase S1C, HrtA/DegP2/Q/S family protein |
|  | | | |  | | Os06t0234150-00 | | 6.90-6.98 | | Non-protein coding transcript |
|  |  |  |  |  | | Os06t0234100-02 | | 6.90-6.98 | | Similar to DEGP9 (DEGP PROTEASE 9); serine-type peptidase/ trypsin |
| 3 | *qPW8-1* | No significantly associated SNPs-candidate genes observed | | | | | | | | |
|  |  |  |  | |  | |  | |  |  |
| List of candidate genes identified in novel QTL regions detected with 24 selected RILs-1,082 SNPs | | | | | | | | | | |
| S.No | QTL name | Chr | QTL region (Mb) | QTL size (Mb) | | Locus ID | | Physical position of candidate gene (Mb) | | Biological function of the candidate gene |
| 1 | *qYLD2-1* | 2 | 5.35-8.22 | 2.87 | | Os02t0202400-01 | | 5.73 | | Starch synthesis during seed development |
|  | | | | | | Os02t0214900-01 | | 6.42-6.43 | | Reproductive development, seed morphology |
|  |  |  |  |  |  | Os02t0234200-01 | | 7.59 | | Determination of panicle architecture, grain shape and grain weight |
|  |  |  |  |  |  | Os02t0244300-01 | | 8.13-8.14 | | Positive regulation of grain width and size |
| 2 | *qDFF2-1* | 2 | 2.90-2.38 | 0.52 | | Os02t0150800-01 | | 2.79-2.80 | | Circadian clock associated-component |
|  | | | | | | Os02t0152500-01 | | 2.87-2.88 | | Positive regulator of flowering, regulation of leaf angle |
| 3 | *qDFF4-1* | 4 | 7.95-6.25 | 1.7 | | Os04t0213100-01 | | 7.55 | | Anther development |
| 4 | *qPW10-1* | 10 | 20.69-9.62 | 11.07 | | Os10t0478000-01 | | 17.88 | | Regulation of inflorescence development |
| 5 | *qPL10-1* | 10 | 20.69-9.62 | 11.07 | | Os10t0478200-01 | | 17.91 | | Starch synthesis, seed development |
|  | | | | | | Os10t0498600-01 | | 19-19.01 | | Regulation of starch biosynthesis |
|  |  |  |  |  |  | Os10t0508100-01 | | 19.46 | | Regulation of grain-filling rate |
| 6 | *qPL3-1* | 3 | 33.43-34.24 | 0.81 | | Os03t0809900-01 | | 33.89 | | Embryonic organogenesis and development |
|  | | | | | | Os03t0815700-01 | | 34.19-34.19 | | Flowering time control |
| 7 | *qFLL6-1* | 6 | 0.08-0.05 | 0.03 | | Os06t0101600-01 | | 0.14-0.15 | | Plastocyanin, chloroplast precursor |
|  | | | | | | Os06t0103600-01 | | 0.23-0.24 | | Chloroplast development, establishment of the plastidic ribosome system |

YLD-Total grain yield/plant(g); DFF-Days to fifty percent flowering; PW-Panicle weight (g); PL-Panicle length (cm); FLL-Flag leaf length (cm)

**Supplementary Table 16:** SNP allelic changes identified in minor effect QTLs

| Minor effect QTLs identified in 105 RILs | | | | | |  |  | High yielding RILs | | | | | | | | | |  | Low yielding RILs | | | | |
| --- | --- | --- | --- | --- | --- | --- | --- | --- | --- | --- | --- | --- | --- | --- | --- | --- | --- | --- | --- | --- | --- | --- | --- |
| QTL name | Flanking SSR or EST-derived SSR markers | SSR marker position (Mb) | SNP marker | SNP marker position (Mb) | SNP allelic change | p-value | R^2^ value  (%) | RIL1&2 | RIL  3&4 | | RIL  5&6 | | RIL  7&8 | | RIL  9&10 | | RIL-  11&12 | RIL-13&14 | RIL-15&16 | RIL-17&18 | RIL-  19&20 | RIL-  21&22 | RIL  23&24 |
| *qDFF9-1* | RM23791-RM24718 | 4.2-20.9 | S9_22015050 | 22.01 | G/A | 4.44E-04 | 72.4 | GG | GG | | GG | | GG | | GG | | GG | AA | AA | AA | AA | AA | AA |
| *qDFF12-1* | RM5479- ESSR12-20.2 | 24.4-20.2 | S12_18759117 | 18.75 | C/A | 4.44E-04 | ------ | CC | CC | | CC | | CC | | CC | | CC | AA | CC | AA | AA | AA | AA |
| *qYLD6-1* | RM7023- ESSR06-7.1 | 6.9-7.1 | S6_6253559 | 6.25 | G/C | 0.02 | 40.3 | GG | GG | | GG | | GG | | GG | | GG | CC | CC | CC | CC | CC | CC |
| *qGP8-1* | RM101- RM27966 | 8.8-12.1 | S8_12267313 | 12.26 | G/T | 0.17 | 28.4 | GG | NN | | GG | | GG | | GG | | GG | TT | TT | TT | TT | TT | TT |
| *qGP12-1* | RM1384- SSR-8-13 | 11.8-13 | S12_12267313 | 12.26 | G/T | 0.17 | ------ | GG | NN | | GG | | GG | | GG | | GG | TT | TT | TT | TT | TT | TT |
| *qFGP8-1* | RM22554-RM1384 | 5.5-11.8 | S8_5248865 | 5.24 | A/G | 5.58E-08 | 96.7 | AA | AA | | AA | | AA | | NN | | AG | AG | AG | AG | AG | AG | AG |
| *qTGW5-1* | RM18414-RM18516 | 15.6-17.3 | S5_15613303 | 15.61 | T/C | 0.02 | 40.9 | TT | TT | | TT | | TT | | TT | | TT | TC | TC | TC | TC | TC | TC |
| *qTGW8-1* | SSR-8-18-RM22837 | 18-12.3 | S8_16307310 | 16.30 | C/T | 0.01 | 42.8 | CC | CC | | CC | | CC | | NN | | CC | TT | TT | TT | TT | TT | TT |
| *qTGW12-1* | RM28275-RM28085 | 19.4-15.3 | S12_19537089 | 19.53 | G/C | 0.16 | 18.2 | GG | GG | | GG | | GG | | GG | | GG | CC | CC | CC | CC | CC | CC |
| *qPW6-1* | RM7023- ESSR06-7.1 | 6.9-7.1 | S6_6907203 | 6.90 | G/C | 0.13 | 38.6 | GG | GG | | GG | | GG | | GG | | GG | CC | CC | CC | CC | NN | NN |
| *qPW8-1* | RM1384-SSR-8-13 | 11.8-13 | No significantly associated SNP alleles found | | | | | | | | | | | | | | | | | | | | |
| *qPH12-2* | RM27966-RM235 | 12.1-26.1 | S12_14867709 | 14.86 | G/A | 0.04 | 44.8 | GG | | GG | | GG | | GG | | NN | GG | AA | AA | AA | AA | AA | AA |
| *qPL6-1* | RM7023- ESSR06-7.1 | 6.9-7.1 | S6_6253559 | 6.25 | G/C | 0.02 | 40.3 | GG | | GG | | GG | | GG | | GG | GG | CC | CC | CC | CC | CC | CC |
| *qPL9-1* | RM23958- RM23959 | 7.94 | S9_7852553 | 7.85 | C/T | 0.01 | 75.7 | TT | | TT | | NN | | NN | | NN | TT | TC | CC | CC | CC | CC | CC |
| *qFLW4-2* | RM252- RM6748 | 24-30.9 | S4_31643336 | 31.64 | C/G | 0.01 | 66.9 | CC | | CC | | CC | | CC | | NN | CC | CG | GG | GG | GG | GG | GG |
| *qBM5-1* | RM18414- RM18516 | 15.6-17.3 | No significantly associated SNP alleles found | | | | | | | | | | | | | | | | | | | | |
| *qBM11-1* | RM286- RM27096 | 0.3-23.4 | S11_848092 | 0.84 | A/T | 0.01 | 92.8 | TA | | NN | | TA | | TA | | TA | TT | TT | TT | TT | TT | TT | TT |

N= Any nucleotide; DFF-Days to 50% percent flowering; YLD-Total grain yield/plant(g); GP-Total grains/panicle; FGP-Fertile grains/panicle; TGW-Test (1000) grain weight (g); PW-Panicle weight (g); PH-Plant height (cm); PL-Panicle length (cm); FLW-Flag leaf width (cm); BM-Biomass (g); R^2^ value (%)-the cumulative phenotypic variance (%)

**Supplementary Table 17:**  A brief account of involvement of identified SNP loci associated with minor effect QTLs in regulating different traits

| Minor effect QTLs identified in 105 RILs | | | | | | Details of earlier reported QTLs/gene(s) from QTARO database | |
| --- | --- | --- | --- | --- | --- | --- | --- |
| QTL name | Flanking SSR or EST-derived SSR markers | SSR marker position (Mb) | SNP marker | SNP marker position (Mb) | Gene(s)/QTL name from previous studies | Reference of previous study | Other prominent functions associated with SNP loci |
| *qDFF9-1* | RM23791-RM24718 | 4.2-20.9 | S9_22015050 | 22.01 | Genes apd9; Sub-1(t); yldp9.1; gy9 | 121, 127, 128, 129 | Albino plantlet differentiation frequency; submergence tolerance; yield per plant; grain yield |
| *qDFF12-1* | RM5479- ESSR12-20.2 | 24.4-20.2 | S12_18759117 | 18.75 | Gene prt12.1 | 130 | Penetrated root thickness, relative root length after 4 weeks stress, conditional Relative root length (4-week/2-week), maximum root length (after 4-weeks stress) |
| *qYLD6-1* | RM7023- ESSR06-7.1 | 6.9-7.1 | S6_6253559 | 6.25 | qLDLJ-6-1,2,3 | 95 | Grain length/width ratio (Ps4 gene), number of late-discoloring leaves per panicle at 25 days after flowering (DAF), amylose content (gene amy6-1), alkali spreading value (gene asv6-1), total number of spikelets/panicle (gene tns6), spikelet density (sd6) |
| *qGP8-1* | RM101- RM27966 | 8.8-12.1 | S8_12267313 | 12.26 | *qDSR 8* | 131 | Dead seedling rate at 62 days, days to heading (gene dth8), root length ratio, heading date (gene hd8), delay in flowering time by drought (DFT), gene hsa2(t) governing F_2_ sterility trait |
| *qGP12-1* | RM1384- SSR-8-13 | 11.8-13 | S12_12267313 | 12.26 | qCGB; *qCTS12*; *qtl12.1* | 132, 133, 90 | Relative tillering ability, relative shoot dry weight, relative root dry weight (earlier reported to be controlled by Pi-4(t) gene under soil stress conditions), eating quality (under the influence of QTL qCGB), thousand grain weight (qCTS12); harvest index, panicle number, flowering delay (*qtl12.1*) |
| *qFGP8-1* | RM22554-RM1384 | 5.5-11.8 | S8_5248865 | 5.24 | qRRE-8; qDSR 8 | 134, 131 | Relative root elongation (for aluminum soil tolerance) and heading date, earlier reported to be controlled by QTL qRRE-8; QTL qDSR8 influencing the dead seedling rate at 62 days under soil stress conditions and grain weight. Gene dth8 controlling the days to heading trait |
| *qTGW5-1* | RM18414-RM18516 | 15.6-17.3 | S5_15613303 | 15.61 | qLTG-5;QKw5 | 135, 136 | Gene yl(t) controlled the yellowish-green leaf character. QTL qLTG-5 was involved in germinability of seed under cold tolerance conditions. QKw5, for 1000 kernel weight |
| *qTGW8-1* | SSR-8-18-RM22837 | 18-12.3 | S8_16307310 | 16.30 | *qALRR-8* | 137 | Gene hd8 controlled the heading date, gene hsa2(t) influenced the F_2_ sterility trait and a QTL, *qALRR-8*, was responsible for root length ratio conferring soil stress tolerance |
| *qTGW12-1* | RM28275-RM28085 | 19.4-15.3 | S12_19537089 | 19.53 | *qCTS12* | 133 | Gene prt12.1 regulated the relative root length after 4 weeks (under soil stress conditions), conditional relative root length between 2-4 weeks and maximum root length after weeks (under soil stress conditions), 1,000 grain weight |
| *qPW6-1* | RM7023- ESSR06-7.1 | 6.9-7.1 | S6_6907203 | 6.90 | qLDLJ-6-1,2,3 | 95 | Grain length/width ratio (Ps4 gene), number of late-discoloring leaves per panicle at 25 days after flowering (DAF), amylose content (gene amy6-1), alkali spreading value (gene asv6-1), total number of spikelets/panicle (gene tns6), spikelet density (sd6) |
| *qPH12-2* | RM27966-RM235 | 12.1-26.1 | S12_14867709 | 14.86 | *qtl12.1* | 90 | Harvest index, panicle number, flowering delay |
|  |  |  |  |  |  |  |  |
| Supplementary Table 17 (continued) | | | | | | | |
| QTL name | Flanking SSR or EST-derived SSR markers | SSR marker position (Mb) | SNP marker | SNP marker position (Mb) | Gene/QTL name | Previous study | Other prominent functions associated with SNP |
| *qPW8-1* | RM1384-SSR-8-13 | 11.8-13 | No significantly associated SNP alleles found | | | | |
| *qPL9-1* | RM23958- RM23959 | 7.94 | S9_7852553 | 7.85 | Genes brt9.1; clr9; apd9; Sub-1(t) | 127, 128, 137, 138 | Gene brt9.1 conferred drought tolerance by promoting maximum root length. Gene clr9 promoted reduction in the ratio of culm length which conferred cold tolerance. Differentiation of albino plant frequency was controlled by gene apd9. Relative water content was regulated by gene Sub-1(t) which conferred drought tolerance |
| *qFLW4-2* | RM252- RM6748 | 24-30.9 | S4_31643336 | 31.64 | *qSC-4* | 139 | Gene pss4.1 influenced the percent seed set, gene rfwa regulated the root fresh weight drought tolerance conditions, *Xa2* conferred bacterial blight resistance, OsAOX1a gene was involved in cold tolerance may be through the synthesis of alternative oxidase. Gene, Gm7, was involved in gall midge resistance and in regulation of chlorophyll content. Genes, phs4.1 and phc4.1 were observed to influence the plant height under stress and control conditions, respectively. *qSC-4* was identified to be associated with panicle flower trait (sink capacity) and in influencing the root thickness. |
| *qBM5-1* | RM18414- RM18516 | 15.6-17.3 | No significantly associated SNP alleles found | | | | |
| *qBM11-1* | RM286- RM27096 | 0.3-23.4 | S11_848092 | 0.84 | *qDLR11* | 131 | Trait number of florets/branch and dead leaf rate at 20 days under soil stress conditions |

**Supplementary Table 18**: Fertility restoration potential of high-yielding RILs

| **S.No** | **Material/**  **Generation (F_8_)** | **Cross description** | **Nature of RIL** | **R*f*3/R*f*4 status** | **Spikelet**  **fertility (%)** | **Restoration status of RIL** |
| --- | --- | --- | --- | --- | --- | --- |
|  |  |  |  |  |  |  |
| 1 | RIL-1 | IR58025A  ×  KMR-3R | High yielding | *Rf3Rf3*/*Rf4Rf4* | 80.97 | Complete restorer |
| 2 | RIL-2 |  |  | *Rf3Rf3*/*rf4rf4* | 63.98 | Partial restorer |
| 3 | RIL-3 |  |  | *Rf3Rf3*/*rf4rf4* | 60.77 | Partial restorer |
| 4 | RIL-4 |  |  | *Rf3Rf3*/*Rf4Rf4* | 83.33 | Complete restorer |
| 5 | RIL-5 |  |  | *Rf3Rf3*/*rf4rf4* | 65.07 | Partial restorer |
| 6 | RIL-6 |  |  | *Rf3Rf3*/*Rf4Rf4* | 70.98 | Complete restorer |
| 7 | RIL-7 |  |  | *Rf3Rf3*/*rf4rf4* | 61.88 | Partial restorer |
| 8 | RIL-8 |  |  | *Rf3Rf3*/*Rf4Rf4* | 83.94 | Complete restorer |
| 9 | RIL-9 |  |  | *Rf3Rf3*/*Rf4Rf4* | 82.92 | Complete restorer |
| 10 | RIL-10 |  |  | *Rf3Rf3*/*rf4rf4* | 67.41 | Partial restorer |
| 11 | RIL-11 |  |  | *Rf3Rf3*/*rf4rf4* | 61.84 | Partial restorer |
| 12 | RIL-12 |  |  | *Rf3Rf3*/*Rf4Rf4* | 75.54 | Complete restorer |

| **Supplementary Table 19**: Details of non-redundant and filtered 1, 082 single nucleotide polymorphic (SNP) markers used for QTL mapping | | | |
| --- | --- | --- | --- |
| S.No | SNP Marker name | Chromosome .no | Physical position (Mb) |
| 1 | S1_242656 | 1 | 0.24 |
| 2 | S1_424861 | 1 | 0.42 |
| 3 | S1_438902 | 1 | 0.44 |
| 4 | S1_439336 | 1 | 0.44 |
| 5 | S1_854371 | 1 | 0.85 |
| 6 | S1_854372 | 1 | 0.85 |
| 7 | S1_854408 | 1 | 0.85 |
| 8 | S1_854780 | 1 | 0.85 |
| 9 | S1_1974991 | 1 | 1.97 |
| 10 | S1_1998887 | 1 | 2.00 |
| 11 | S1_2080373 | 1 | 2.08 |
| 12 | S1_2389018 | 1 | 2.39 |
| 13 | S1_2840176 | 1 | 2.84 |
| 14 | S1_2852608 | 1 | 2.85 |
| 15 | S1_2978860 | 1 | 2.98 |
| 16 | S1_3055761 | 1 | 3.06 |
| 17 | S1_3124640 | 1 | 3.12 |
| 18 | S1_3407755 | 1 | 3.41 |
| 19 | S1_3599057 | 1 | 3.60 |
| 20 | S1_8259930 | 1 | 8.26 |
| 21 | S1_12423863 | 1 | 12.42 |
| 22 | S1_14615154 | 1 | 14.62 |
| 23 | S1_16766499 | 1 | 16.77 |
| 24 | S1_19134717 | 1 | 19.13 |
| 25 | S1_20363714 | 1 | 20.36 |
| 26 | S1_22234246 | 1 | 22.23 |
| 27 | S1_26923081 | 1 | 26.92 |
| 28 | S1_27291965 | 1 | 27.29 |
| 29 | S1_29213876 | 1 | 29.21 |
| 30 | S1_30157628 | 1 | 30.16 |
| 31 | S1_30359793 | 1 | 30.36 |
| 32 | S1_30461153 | 1 | 30.46 |
| 33 | S1_30658639 | 1 | 30.66 |
| 34 | S1_30933825 | 1 | 30.93 |
| 35 | S1_31560212 | 1 | 31.56 |
| 36 | S1_31811187 | 1 | 31.81 |
| 37 | S1_32109172 | 1 | 32.11 |
| 38 | S1_32113968 | 1 | 32.11 |
| 39 | S1_32146755 | 1 | 32.15 |
| 40 | S1_32807560 | 1 | 32.81 |
| 41 | S1_32956705 | 1 | 32.96 |
| 42 | S1_33055101 | 1 | 33.06 |
| 43 | S1_33330967 | 1 | 33.33 |
| 44 | S1_33540765 | 1 | 33.54 |
| 45 | S1_33629990 | 1 | 33.63 |
| 46 | S1_33732799 | 1 | 33.73 |
| 47 | S2_463582 | 2 | 0.46 |
| 48 | S2_576644 | 2 | 0.58 |
| 49 | S2_672630 | 2 | 0.67 |
| 50 | S2_940772 | 2 | 0.94 |
| Supplementary Table 19 (continued) | | | |
| S.No | SNP Marker name | Chromosome .no | Physical position (Mb) |
| 51 | S2_981470 | 2 | 0.98 |
| 52 | S2_1106686 | 2 | 1.11 |
| 53 | S2_1337341 | 2 | 1.34 |
| 54 | S2_1346470 | 2 | 1.35 |
| 55 | S2_1757214 | 2 | 1.76 |
| 56 | S2_1838595 | 2 | 1.84 |
| 57 | S2_1864135 | 2 | 1.86 |
| 58 | S2_1914079 | 2 | 1.91 |
| 59 | S2_2344911 | 2 | 2.34 |
| 60 | S2_2387051 | 2 | 2.39 |
| 61 | S2_2426938 | 2 | 2.43 |
| 62 | S2_2546481 | 2 | 2.55 |
| 63 | S2_2653118 | 2 | 2.65 |
| 64 | S2_2731576 | 2 | 2.73 |
| 65 | S2_2907132 | 2 | 2.91 |
| 66 | S2_2948202 | 2 | 2.95 |
| 67 | S2_2948508 | 2 | 2.95 |
| 68 | S2_2948526 | 2 | 2.95 |
| 69 | S2_2961842 | 2 | 2.96 |
| 70 | S2_2983521 | 2 | 2.98 |
| 71 | S2_2983522 | 2 | 2.98 |
| 72 | S2_2983524 | 2 | 2.98 |
| 73 | S2_2983934 | 2 | 2.98 |
| 74 | S2_3037922 | 2 | 3.04 |
| 75 | S2_3037929 | 2 | 3.04 |
| 76 | S2_3037944 | 2 | 3.04 |
| 77 | S2_3038145 | 2 | 3.04 |
| 78 | S2_3050619 | 2 | 3.05 |
| 79 | S2_3050636 | 2 | 3.05 |
| 80 | S2_3086604 | 2 | 3.09 |
| 81 | S2_3094543 | 2 | 3.09 |
| 82 | S2_3121922 | 2 | 3.12 |
| 83 | S2_3126357 | 2 | 3.13 |
| 84 | S2_3169145 | 2 | 3.17 |
| 85 | S2_4056976 | 2 | 4.06 |
| 86 | S2_4075635 | 2 | 4.08 |
| 87 | S2_4288727 | 2 | 4.29 |
| 88 | S2_4559875 | 2 | 4.56 |
| 89 | S2_4937255 | 2 | 4.94 |
| 90 | S2_5262174 | 2 | 5.26 |
| 91 | S2_5262516 | 2 | 5.26 |
| 92 | S2_5359418 | 2 | 5.36 |
| 93 | S2_6058563 | 2 | 6.06 |
| 94 | S2_6278645 | 2 | 6.28 |
| 95 | S2_6452455 | 2 | 6.45 |
| 96 | S2_8229921 | 2 | 8.23 |
| 97 | S2_9923657 | 2 | 9.92 |
| 98 | S2_9923685 | 2 | 9.92 |
| 99 | S2_9975962 | 2 | 9.98 |
| 100 | S2_13706741 | 2 | 13.71 |
| 101 | S2_16789618 | 2 | 16.79 |
| 102 | S2_17981703 | 2 | 17.98 |
| 103 | S2_19594426 | 2 | 19.59 |
| 104 | S2_20227574 | 2 | 20.23 |
| 105 | S2_20565910 | 2 | 20.57 |
| 106 | S2_23482918 | 2 | 23.48 |
| 107 | S2_23944127 | 2 | 23.94 |
| 108 | S2_23964581 | 2 | 23.96 |
| 109 | S2_24096140 | 2 | 24.10 |
| 110 | S2_24249727 | 2 | 24.25 |
| 111 | S2_24278397 | 2 | 24.28 |
| 112 | S2_25369610 | 2 | 25.37 |
| 113 | S2_25460419 | 2 | 25.46 |
| 114 | S2_25654891 | 2 | 25.65 |
| 115 | S2_26477186 | 2 | 26.48 |
| 116 | S2_28723031 | 2 | 28.72 |
| 117 | S2_29073012 | 2 | 29.07 |
| 118 | S2_29331093 | 2 | 29.33 |
| 119 | S2_29579060 | 2 | 29.58 |
| 120 | S2_31093457 | 2 | 31.09 |
| 121 | S2_31666003 | 2 | 31.67 |
| 122 | S2_31725636 | 2 | 31.73 |
| 123 | S2_32166043 | 2 | 32.17 |
| 124 | S2_32249957 | 2 | 32.25 |
| 125 | S2_35261285 | 2 | 35.26 |
| 126 | S3_446043 | 3 | 0.45 |
| 127 | S3_656451 | 3 | 0.66 |
| 128 | S3_665830 | 3 | 0.67 |
| 129 | S3_774624 | 3 | 0.77 |
| 130 | S3_878982 | 3 | 0.88 |
| 131 | S3_1143301 | 3 | 1.14 |
| 132 | S3_1395479 | 3 | 1.40 |
| 133 | S3_1920953 | 3 | 1.92 |
| 134 | S3_3095743 | 3 | 3.10 |
| 135 | S3_3265120 | 3 | 3.27 |
| 136 | S3_3268064 | 3 | 3.27 |
| 137 | S3_3276056 | 3 | 3.28 |
| 138 | S3_4539636 | 3 | 4.54 |
| 139 | S3_4675885 | 3 | 4.68 |
| 140 | S3_4699308 | 3 | 4.70 |
| 141 | S3_6109552 | 3 | 6.11 |
| 142 | S3_6134304 | 3 | 6.13 |
| 143 | S3_6806897 | 3 | 6.81 |
| 144 | S3_6863874 | 3 | 6.86 |
| 145 | S3_6981976 | 3 | 6.98 |
| 146 | S3_6982005 | 3 | 6.98 |
| 147 | S3_7090046 | 3 | 7.09 |
| 148 | S3_7128653 | 3 | 7.13 |
| 149 | S3_7245581 | 3 | 7.25 |
| 150 | S3_7307878 | 3 | 7.31 |
| 151 | S3_7481368 | 3 | 7.48 |
| 152 | S3_7566599 | 3 | 7.57 |
| Supplementary Table 19 (continued) | | | |
| S.No | SNP Marker name | Chromosome .no | Physical position (Mb) |
| 153 | S3_7788603 | 3 | 7.79 |
| 154 | S3_7812695 | 3 | 7.81 |
| 155 | S3_7832733 | 3 | 7.83 |
| 156 | S3_7845032 | 3 | 7.85 |
| 157 | S3_7884776 | 3 | 7.88 |
| 158 | S3_8076545 | 3 | 8.08 |
| 159 | S3_8076560 | 3 | 8.08 |
| 160 | S3_8090654 | 3 | 8.09 |
| 161 | S3_10973562 | 3 | 10.97 |
| 162 | S3_11128087 | 3 | 11.13 |
| 163 | S3_13473282 | 3 | 13.47 |
| 164 | S3_13478207 | 3 | 13.48 |
| 165 | S3_13587616 | 3 | 13.59 |
| 166 | S3_14144714 | 3 | 14.14 |
| 167 | S3_14266372 | 3 | 14.27 |
| 168 | S3_14623306 | 3 | 14.62 |
| 169 | S3_26885698 | 3 | 26.89 |
| 170 | S3_26886015 | 3 | 26.89 |
| 171 | S3_27516811 | 3 | 27.52 |
| 172 | S3_27704104 | 3 | 27.70 |
| 173 | S3_28608794 | 3 | 28.61 |
| 174 | S3_28664885 | 3 | 28.66 |
| 175 | S3_28817372 | 3 | 28.82 |
| 176 | S3_28996950 | 3 | 29.00 |
| 177 | S3_29019589 | 3 | 29.02 |
| 178 | S3_29138042 | 3 | 29.14 |
| 179 | S3_29184661 | 3 | 29.18 |
| 180 | S3_29326984 | 3 | 29.33 |
| 181 | S3_29421235 | 3 | 29.42 |
| 182 | S3_29475685 | 3 | 29.48 |
| 183 | S3_29742394 | 3 | 29.74 |
| 184 | S3_29775682 | 3 | 29.78 |
| 185 | S3_29889254 | 3 | 29.89 |
| 186 | S3_29986460 | 3 | 29.99 |
| 187 | S3_31008889 | 3 | 31.01 |
| 188 | S3_31028827 | 3 | 31.03 |
| 189 | S3_31033330 | 3 | 31.03 |
| 190 | S3_31317986 | 3 | 31.32 |
| 191 | S3_31471067 | 3 | 31.47 |
| 192 | S3_31528278 | 3 | 31.53 |
| 193 | S3_31565334 | 3 | 31.57 |
| 194 | S3_31694234 | 3 | 31.69 |
| 195 | S3_31750285 | 3 | 31.75 |
| 196 | S3_31943730 | 3 | 31.94 |
| 197 | S3_32814970 | 3 | 32.81 |
| 198 | S3_33060454 | 3 | 33.06 |
| 199 | S3_33349537 | 3 | 33.35 |
| 200 | S3_33431112 | 3 | 33.43 |
| 201 | S3_33510684 | 3 | 33.51 |
| 202 | S3_33661275 | 3 | 33.66 |
| Supplementary Table 19 (continued) | | | |
| S.No | SNP Marker name | Chromosome .no | Physical position (Mb) |
| 203 | S3_33688871 | 3 | 33.69 |
| 204 | S3_33779823 | 3 | 33.78 |
| 205 | S3_33803875 | 3 | 33.80 |
| 206 | S3_34241104 | 3 | 34.24 |
| 207 | S3_34332642 | 3 | 34.33 |
| 208 | S3_34431671 | 3 | 34.43 |
| 209 | S3_34491593 | 3 | 34.49 |
| 210 | S3_34519750 | 3 | 34.52 |
| 211 | S3_34666402 | 3 | 34.67 |
| 212 | S3_34763865 | 3 | 34.76 |
| 213 | S3_34773920 | 3 | 34.77 |
| 214 | S3_34786863 | 3 | 34.79 |
| 215 | S3_35054373 | 3 | 35.05 |
| 216 | S3_35277451 | 3 | 35.28 |
| 217 | S3_36064855 | 3 | 36.06 |
| 218 | S4_411524 | 4 | 0.41 |
| 219 | S4_1791821 | 4 | 1.79 |
| 220 | S4_1816991 | 4 | 1.82 |
| 221 | S4_3411049 | 4 | 3.41 |
| 222 | S4_3635666 | 4 | 3.64 |
| 223 | S4_4513315 | 4 | 4.51 |
| 224 | S4_4588939 | 4 | 4.59 |
| 225 | S4_4595535 | 4 | 4.60 |
| 226 | S4_4739773 | 4 | 4.74 |
| 227 | S4_5162078 | 4 | 5.16 |
| 228 | S4_5379415 | 4 | 5.38 |
| 229 | S4_5544411 | 4 | 5.54 |
| 230 | S4_6091418 | 4 | 6.09 |
| 231 | S4_6251080 | 4 | 6.25 |
| 232 | S4_6593508 | 4 | 6.59 |
| 233 | S4_7141609 | 4 | 7.14 |
| 234 | S4_7923954 | 4 | 7.92 |
| 235 | S4_7956048 | 4 | 7.96 |
| 236 | S4_8484646 | 4 | 8.48 |
| 237 | S4_9029814 | 4 | 9.03 |
| 238 | S4_10183781 | 4 | 10.18 |
| 239 | S4_10247581 | 4 | 10.25 |
| 240 | S4_10247600 | 4 | 10.25 |
| 241 | S4_11062780 | 4 | 11.06 |
| 242 | S4_11575519 | 4 | 11.58 |
| 243 | S4_11917507 | 4 | 11.92 |
| 244 | S4_12002281 | 4 | 12.00 |
| 245 | S4_12397212 | 4 | 12.40 |
| 246 | S4_13523390 | 4 | 13.52 |
| 247 | S4_13914413 | 4 | 13.91 |
| 248 | S4_14135184 | 4 | 14.14 |
| 249 | S4_14209970 | 4 | 14.21 |
| 250 | S4_14210003 | 4 | 14.21 |
| 251 | S4_14921118 | 4 | 14.92 |
| 252 | S4_14935611 | 4 | 14.94 |
| Supplementary Table 19 (continued) | | | |
| S.No | SNP Marker name | Chromosome .no | Physical position (Mb) |
| 253 | S4_15067196 | 4 | 15.07 |
| 254 | S4_15067298 | 4 | 15.07 |
| 255 | S4_15489934 | 4 | 15.49 |
| 256 | S4_17868178 | 4 | 17.87 |
| 257 | S4_17868180 | 4 | 17.87 |
| 258 | S4_17868182 | 4 | 17.87 |
| 259 | S4_17868183 | 4 | 17.87 |
| 260 | S4_17868188 | 4 | 17.87 |
| 261 | S4_17908884 | 4 | 17.91 |
| 262 | S4_17908902 | 4 | 17.91 |
| 263 | S4_17908909 | 4 | 17.91 |
| 264 | S4_17909110 | 4 | 17.91 |
| 265 | S4_17909119 | 4 | 17.91 |
| 266 | S4_18215032 | 4 | 18.22 |
| 267 | S4_18215053 | 4 | 18.22 |
| 268 | S4_18215443 | 4 | 18.22 |
| 269 | S4_18470767 | 4 | 18.47 |
| 270 | S4_18470773 | 4 | 18.47 |
| 271 | S4_18470859 | 4 | 18.47 |
| 272 | S4_18470866 | 4 | 18.47 |
| 273 | S4_18470882 | 4 | 18.47 |
| 274 | S4_18470901 | 4 | 18.47 |
| 275 | S4_18471095 | 4 | 18.47 |
| 276 | S4_18763820 | 4 | 18.76 |
| 277 | S4_18935406 | 4 | 18.94 |
| 278 | S4_19001963 | 4 | 19.00 |
| 279 | S4_19001987 | 4 | 19.00 |
| 280 | S4_19001994 | 4 | 19.00 |
| 281 | S4_19002011 | 4 | 19.00 |
| 282 | S4_19009273 | 4 | 19.01 |
| 283 | S4_23173224 | 4 | 23.17 |
| 284 | S4_23345327 | 4 | 23.35 |
| 285 | S4_23508890 | 4 | 23.51 |
| 286 | S4_23508984 | 4 | 23.51 |
| 287 | S4_23512981 | 4 | 23.51 |
| 288 | S4_23964601 | 4 | 23.96 |
| 289 | S4_23964604 | 4 | 23.96 |
| 290 | S4_24002530 | 4 | 24.00 |
| 291 | S4_24002558 | 4 | 24.00 |
| 292 | S4_24030970 | 4 | 24.03 |
| 293 | S4_24156867 | 4 | 24.16 |
| 294 | S4_24164038 | 4 | 24.16 |
| 295 | S4_24265926 | 4 | 24.27 |
| 296 | S4_24266140 | 4 | 24.27 |
| 297 | S4_24302396 | 4 | 24.30 |
| 298 | S4_27568586 | 4 | 27.57 |
| 299 | S4_29297032 | 4 | 29.30 |
| 300 | S4_29297062 | 4 | 29.30 |
| 301 | S4_29355978 | 4 | 29.36 |
| 302 | S4_29501423 | 4 | 29.50 |
| Supplementary Table 19 (continued) | | | |
| S.No | SNP Marker name | Chromosome .no | Physical position (Mb) |
| 303 | S4_29969421 | 4 | 29.97 |
| 304 | S4_29973768 | 4 | 29.97 |
| 305 | S4_29973798 | 4 | 29.97 |
| 306 | S4_29974033 | 4 | 29.97 |
| 307 | S4_30161350 | 4 | 30.16 |
| 308 | S4_30483194 | 4 | 30.48 |
| 309 | S4_30690751 | 4 | 30.69 |
| 310 | S4_30790151 | 4 | 30.79 |
| 311 | S4_30790171 | 4 | 30.79 |
| 312 | S4_31643336 | 4 | 31.64 |
| 313 | S4_31643362 | 4 | 31.64 |
| 314 | S4_31661222 | 4 | 31.66 |
| 315 | S4_31661652 | 4 | 31.66 |
| 316 | S4_31780323 | 4 | 31.78 |
| 317 | S4_31973372 | 4 | 31.97 |
| 318 | S4_32059735 | 4 | 32.06 |
| 319 | S4_32142246 | 4 | 32.14 |
| 320 | S4_32255682 | 4 | 32.26 |
| 321 | S4_32411119 | 4 | 32.41 |
| 322 | S4_34300927 | 4 | 34.30 |
| 323 | S4_34626250 | 4 | 34.63 |
| 324 | S4_34843167 | 4 | 34.84 |
| 325 | S4_34899460 | 4 | 34.90 |
| 326 | S5_232201 | 5 | 0.23 |
| 327 | S5_686983 | 5 | 0.69 |
| 328 | S5_936187 | 5 | 0.94 |
| 329 | S5_1114939 | 5 | 1.11 |
| 330 | S5_1129294 | 5 | 1.13 |
| 331 | S5_3361011 | 5 | 3.36 |
| 332 | S5_5190370 | 5 | 5.19 |
| 333 | S5_6419737 | 5 | 6.42 |
| 334 | S5_6967783 | 5 | 6.97 |
| 335 | S5_14948395 | 5 | 14.95 |
| 336 | S5_15050022 | 5 | 15.05 |
| 337 | S5_15613303 | 5 | 15.61 |
| 338 | S5_18319758 | 5 | 18.32 |
| 339 | S5_18405433 | 5 | 18.41 |
| 340 | S5_18428560 | 5 | 18.43 |
| 341 | S5_18701956 | 5 | 18.70 |
| 342 | S5_18937139 | 5 | 18.94 |
| 343 | S5_18942588 | 5 | 18.94 |
| 344 | S5_19057186 | 5 | 19.06 |
| 345 | S5_19215973 | 5 | 19.22 |
| 346 | S5_19258476 | 5 | 19.26 |
| 347 | S5_19504715 | 5 | 19.50 |
| 348 | S5_19548916 | 5 | 19.55 |
| 349 | S5_19805411 | 5 | 19.81 |
| 350 | S5_19830042 | 5 | 19.83 |
| 351 | S5_20289439 | 5 | 20.29 |
| 352 | S5_20538166 | 5 | 20.54 |
| Supplementary Table 19 (continued) | | | |
| S.No | SNP Marker name | Chromosome .no | Physical position (Mb) |
| 353 | S5_20972075 | 5 | 20.97 |
| 354 | S5_21392610 | 5 | 21.39 |
| 355 | S5_21454464 | 5 | 21.45 |
| 356 | S5_21930900 | 5 | 21.93 |
| 357 | S5_24242729 | 5 | 24.24 |
| 358 | S5_25954805 | 5 | 25.95 |
| 359 | S5_26595923 | 5 | 26.60 |
| 360 | S5_27356817 | 5 | 27.36 |
| 361 | S5_27787554 | 5 | 27.79 |
| 362 | S5_27838477 | 5 | 27.84 |
| 363 | S5_29804308 | 5 | 29.80 |
| 364 | S5_29804311 | 5 | 29.80 |
| 365 | S5_29821300 | 5 | 29.82 |
| 366 | S5_29831189 | 5 | 29.83 |
| 367 | S5_29831200 | 5 | 29.83 |
| 368 | S5_29835169 | 5 | 29.84 |
| 369 | S5_29844731 | 5 | 29.84 |
| 370 | S5_29845124 | 5 | 29.85 |
| 371 | S5_29846143 | 5 | 29.85 |
| 372 | S5_29849125 | 5 | 29.85 |
| 373 | S5_29850561 | 5 | 29.85 |
| 374 | S5_29850901 | 5 | 29.85 |
| 375 | S5_29851293 | 5 | 29.85 |
| 376 | S5_29851307 | 5 | 29.85 |
| 377 | S5_29851336 | 5 | 29.85 |
| 378 | S5_29852050 | 5 | 29.85 |
| 379 | S5_29853834 | 5 | 29.85 |
| 380 | S5_29856421 | 5 | 29.86 |
| 381 | S5_29885191 | 5 | 29.89 |
| 382 | S5_29890927 | 5 | 29.89 |
| 383 | S5_29891219 | 5 | 29.89 |
| 384 | S5_29905083 | 5 | 29.91 |
| 385 | S5_29905116 | 5 | 29.91 |
| 386 | S5_29905122 | 5 | 29.91 |
| 387 | S5_29905135 | 5 | 29.91 |
| 388 | S5_29906178 | 5 | 29.91 |
| 389 | S5_29906179 | 5 | 29.91 |
| 390 | S5_29906194 | 5 | 29.91 |
| 391 | S5_29906212 | 5 | 29.91 |
| 392 | S5_29906223 | 5 | 29.91 |
| 393 | S5_29906255 | 5 | 29.91 |
| 394 | S5_29920907 | 5 | 29.92 |
| 395 | S5_29921938 | 5 | 29.92 |
| 396 | S5_29922007 | 5 | 29.92 |
| 397 | S5_29923667 | 5 | 29.92 |
| 398 | S5_29923679 | 5 | 29.92 |
| 399 | S5_29923683 | 5 | 29.92 |
| 400 | S5_29923691 | 5 | 29.92 |
| 401 | S5_29923698 | 5 | 29.92 |
| 402 | S5_29923699 | 5 | 29.92 |
| Supplementary Table 19 (continued) | | | |
| S.No | SNP Marker name | Chromosome .no | Physical position (Mb) |
| 403 | S5_29923700 | 5 | 29.92 |
| 404 | S5_29923712 | 5 | 29.92 |
| 405 | S5_29923714 | 5 | 29.92 |
| 406 | S5_29923718 | 5 | 29.92 |
| 407 | S5_29924037 | 5 | 29.92 |
| 408 | S5_29924058 | 5 | 29.92 |
| 409 | S5_29924066 | 5 | 29.92 |
| 410 | S5_29924366 | 5 | 29.92 |
| 411 | S5_29924373 | 5 | 29.92 |
| 412 | S5_29924378 | 5 | 29.92 |
| 413 | S5_29924392 | 5 | 29.92 |
| 414 | S5_29924398 | 5 | 29.92 |
| 415 | S5_29924402 | 5 | 29.92 |
| 416 | S5_29924411 | 5 | 29.92 |
| 417 | S5_29924418 | 5 | 29.92 |
| 418 | S5_29924419 | 5 | 29.92 |
| 419 | S5_29924718 | 5 | 29.92 |
| 420 | S5_29924769 | 5 | 29.92 |
| 421 | S5_29924771 | 5 | 29.92 |
| 422 | S5_29925080 | 5 | 29.93 |
| 423 | S5_29925086 | 5 | 29.93 |
| 424 | S5_29926131 | 5 | 29.93 |
| 425 | S5_29926185 | 5 | 29.93 |
| 426 | S5_29926201 | 5 | 29.93 |
| 427 | S5_29926202 | 5 | 29.93 |
| 428 | S5_29926203 | 5 | 29.93 |
| 429 | S5_29926205 | 5 | 29.93 |
| 430 | S5_29926207 | 5 | 29.93 |
| 431 | S5_29926211 | 5 | 29.93 |
| 432 | S5_29926225 | 5 | 29.93 |
| 433 | S5_29926239 | 5 | 29.93 |
| 434 | S5_29926240 | 5 | 29.93 |
| 435 | S5_29926241 | 5 | 29.93 |
| 436 | S5_29926244 | 5 | 29.93 |
| 437 | S5_29926246 | 5 | 29.93 |
| 438 | S5_29926247 | 5 | 29.93 |
| 439 | S5_29927259 | 5 | 29.93 |
| 440 | S5_29927262 | 5 | 29.93 |
| 441 | S5_29929339 | 5 | 29.93 |
| 442 | S5_29929342 | 5 | 29.93 |
| 443 | S5_29929351 | 5 | 29.93 |
| 444 | S5_29931082 | 5 | 29.93 |
| 445 | S5_29933496 | 5 | 29.93 |
| 446 | S5_29933506 | 5 | 29.93 |
| 447 | S5_29936001 | 5 | 29.94 |
| 448 | S5_29936008 | 5 | 29.94 |
| 449 | S5_29938437 | 5 | 29.94 |
| 450 | S5_29938447 | 5 | 29.94 |
| 451 | S5_29938470 | 5 | 29.94 |
| 452 | S5_29940199 | 5 | 29.94 |
| Supplementary Table 19 (continued) | | | |
| S.No | SNP Marker name | Chromosome .no | Physical position (Mb) |
| 453 | S5_29940223 | 5 | 29.94 |
| 454 | S5_29940226 | 5 | 29.94 |
| 455 | S5_29940230 | 5 | 29.94 |
| 456 | S5_29943392 | 5 | 29.94 |
| 457 | S5_29943443 | 5 | 29.94 |
| 458 | S5_29943448 | 5 | 29.94 |
| 459 | S5_29944514 | 5 | 29.94 |
| 460 | S5_29945592 | 5 | 29.95 |
| 461 | S5_29946286 | 5 | 29.95 |
| 462 | S5_29946288 | 5 | 29.95 |
| 463 | S5_29946297 | 5 | 29.95 |
| 464 | S5_29946298 | 5 | 29.95 |
| 465 | S5_29946299 | 5 | 29.95 |
| 466 | S5_29946300 | 5 | 29.95 |
| 467 | S5_29946304 | 5 | 29.95 |
| 468 | S5_29946319 | 5 | 29.95 |
| 469 | S5_29946320 | 5 | 29.95 |
| 470 | S5_29950375 | 5 | 29.95 |
| 471 | S6_19627 | 6 | 0.02 |
| 472 | S6_19641 | 6 | 0.02 |
| 473 | S6_19642 | 6 | 0.02 |
| 474 | S6_25237 | 6 | 0.03 |
| 475 | S6_30560 | 6 | 0.03 |
| 476 | S6_30561 | 6 | 0.03 |
| 477 | S6_30563 | 6 | 0.03 |
| 478 | S6_30564 | 6 | 0.03 |
| 479 | S6_30602 | 6 | 0.03 |
| 480 | S6_31624 | 6 | 0.03 |
| 481 | S6_32264 | 6 | 0.03 |
| 482 | S6_32302 | 6 | 0.03 |
| 483 | S6_32686 | 6 | 0.03 |
| 484 | S6_32706 | 6 | 0.03 |
| 485 | S6_47371 | 6 | 0.05 |
| 486 | S6_47374 | 6 | 0.05 |
| 487 | S6_47395 | 6 | 0.05 |
| 488 | S6_47422 | 6 | 0.05 |
| 489 | S6_56355 | 6 | 0.06 |
| 490 | S6_56401 | 6 | 0.06 |
| 491 | S6_56709 | 6 | 0.06 |
| 492 | S6_60262 | 6 | 0.06 |
| 493 | S6_60303 | 6 | 0.06 |
| 494 | S6_67852 | 6 | 0.07 |
| 495 | S6_68912 | 6 | 0.07 |
| 496 | S6_72619 | 6 | 0.07 |
| 497 | S6_77578 | 6 | 0.08 |
| 498 | S6_77586 | 6 | 0.08 |
| 499 | S6_77588 | 6 | 0.08 |
| 500 | S6_77606 | 6 | 0.08 |
| 501 | S6_79694 | 6 | 0.08 |
| 502 | S6_79707 | 6 | 0.08 |
| Supplementary Table 19 (continued) | | | |
| S.No | SNP Marker name | Chromosome .no | Physical position (Mb) |
| 503 | S6_79720 | 6 | 0.08 |
| 504 | S6_79737 | 6 | 0.08 |
| 505 | S6_79742 | 6 | 0.08 |
| 506 | S6_79745 | 6 | 0.08 |
| 507 | S6_81807 | 6 | 0.08 |
| 508 | S6_81827 | 6 | 0.08 |
| 509 | S6_81839 | 6 | 0.08 |
| 510 | S6_81852 | 6 | 0.08 |
| 511 | S6_81861 | 6 | 0.08 |
| 512 | S6_83292 | 6 | 0.08 |
| 513 | S6_83303 | 6 | 0.08 |
| 514 | S6_83312 | 6 | 0.08 |
| 515 | S6_83313 | 6 | 0.08 |
| 516 | S6_83314 | 6 | 0.08 |
| 517 | S6_83315 | 6 | 0.08 |
| 518 | S6_83319 | 6 | 0.08 |
| 519 | S6_83321 | 6 | 0.08 |
| 520 | S6_83333 | 6 | 0.08 |
| 521 | S6_83337 | 6 | 0.08 |
| 522 | S6_84989 | 6 | 0.08 |
| 523 | S6_84993 | 6 | 0.08 |
| 524 | S6_85001 | 6 | 0.09 |
| 525 | S6_85008 | 6 | 0.09 |
| 526 | S6_85017 | 6 | 0.09 |
| 527 | S6_85042 | 6 | 0.09 |
| 528 | S6_85063 | 6 | 0.09 |
| 529 | S6_85071 | 6 | 0.09 |
| 530 | S6_85098 | 6 | 0.09 |
| 531 | S6_85111 | 6 | 0.09 |
| 532 | S6_85422 | 6 | 0.09 |
| 533 | S6_86130 | 6 | 0.09 |
| 534 | S6_86132 | 6 | 0.09 |
| 535 | S6_86137 | 6 | 0.09 |
| 536 | S6_86164 | 6 | 0.09 |
| 537 | S6_87110 | 6 | 0.09 |
| 538 | S6_87112 | 6 | 0.09 |
| 539 | S6_87115 | 6 | 0.09 |
| 540 | S6_87116 | 6 | 0.09 |
| 541 | S6_87128 | 6 | 0.09 |
| 542 | S6_87133 | 6 | 0.09 |
| 543 | S6_87136 | 6 | 0.09 |
| 544 | S6_87140 | 6 | 0.09 |
| 545 | S6_87154 | 6 | 0.09 |
| 546 | S6_87162 | 6 | 0.09 |
| 547 | S6_87164 | 6 | 0.09 |
| 548 | S6_87464 | 6 | 0.09 |
| 549 | S6_87493 | 6 | 0.09 |
| 550 | S6_87505 | 6 | 0.09 |
| 551 | S6_87510 | 6 | 0.09 |
| 552 | S6_91548 | 6 | 0.09 |
| Supplementary Table 19 (continued) | | | |
| S.No | SNP Marker name | Chromosome .no | Physical position (Mb) |
| 553 | S6_91552 | 6 | 0.09 |
| 554 | S6_91589 | 6 | 0.09 |
| 555 | S6_96961 | 6 | 0.10 |
| 556 | S6_96979 | 6 | 0.10 |
| 557 | S6_97004 | 6 | 0.10 |
| 558 | S6_97038 | 6 | 0.10 |
| 559 | S6_953152 | 6 | 0.95 |
| 560 | S6_953153 | 6 | 0.95 |
| 561 | S6_1096463 | 6 | 1.10 |
| 562 | S6_1138013 | 6 | 1.14 |
| 563 | S6_4916869 | 6 | 4.92 |
| 564 | S6_5667602 | 6 | 5.67 |
| 565 | S6_5667712 | 6 | 5.67 |
| 566 | S6_5857298 | 6 | 5.86 |
| 567 | S6_5857307 | 6 | 5.86 |
| 568 | S6_6149846 | 6 | 6.15 |
| 569 | S6_6253105 | 6 | 6.25 |
| 570 | S6_6486511 | 6 | 6.49 |
| 571 | S6_6486525 | 6 | 6.49 |
| 572 | S6_6884257 | 6 | 6.88 |
| 573 | S6_6907203 | 6 | 6.91 |
| 574 | S6_6950356 | 6 | 6.95 |
| 575 | S6_7843390 | 6 | 7.84 |
| 576 | S6_8957266 | 6 | 8.96 |
| 577 | S6_8981719 | 6 | 8.98 |
| 578 | S6_9289669 | 6 | 9.29 |
| 579 | S6_9316469 | 6 | 9.32 |
| 580 | S6_9334468 | 6 | 9.33 |
| 581 | S6_9371579 | 6 | 9.37 |
| 582 | S6_9911594 | 6 | 9.91 |
| 583 | S6_10692972 | 6 | 10.69 |
| 584 | S6_10740891 | 6 | 10.74 |
| 585 | S6_10757246 | 6 | 10.76 |
| 586 | S6_11503558 | 6 | 11.50 |
| 587 | S6_11616180 | 6 | 11.62 |
| 588 | S6_11672144 | 6 | 11.67 |
| 589 | S6_11800454 | 6 | 11.80 |
| 590 | S6_11889981 | 6 | 11.89 |
| 591 | S6_11976054 | 6 | 11.98 |
| 592 | S6_12056157 | 6 | 12.06 |
| 593 | S6_14422811 | 6 | 14.42 |
| 594 | S6_15578248 | 6 | 15.58 |
| 595 | S6_15816689 | 6 | 15.82 |
| 596 | S6_16011513 | 6 | 16.01 |
| 597 | S6_16391459 | 6 | 16.39 |
| 598 | S6_16607928 | 6 | 16.61 |
| 599 | S6_17170461 | 6 | 17.17 |
| 600 | S6_19569952 | 6 | 19.57 |
| 601 | S6_19828074 | 6 | 19.83 |
| 602 | S6_19828080 | 6 | 19.83 |
| Supplementary Table 19 (continued) | | | |
| S.No | SNP Marker name | Chromosome .no | Physical position (Mb) |
| 603 | S6_20192610 | 6 | 20.19 |
| 604 | S6_20540783 | 6 | 20.54 |
| 605 | S6_21128911 | 6 | 21.13 |
| 606 | S6_21829938 | 6 | 21.83 |
| 607 | S6_23451809 | 6 | 23.45 |
| 608 | S6_23590329 | 6 | 23.59 |
| 609 | S6_28046030 | 6 | 28.05 |
| 610 | S6_28674461 | 6 | 28.67 |
| 611 | S6_28734369 | 6 | 28.73 |
| 612 | S6_28873556 | 6 | 28.87 |
| 613 | S6_28908220 | 6 | 28.91 |
| 614 | S6_29447082 | 6 | 29.45 |
| 615 | S6_29541114 | 6 | 29.54 |
| 616 | S6_29577905 | 6 | 29.58 |
| 617 | S6_29849321 | 6 | 29.85 |
| 618 | S6_29849353 | 6 | 29.85 |
| 619 | S6_29886656 | 6 | 29.89 |
| 620 | S6_29886688 | 6 | 29.89 |
| 621 | S6_29891345 | 6 | 29.89 |
| 622 | S6_30787265 | 6 | 30.79 |
| 623 | S7_40650 | 7 | 0.04 |
| 624 | S7_48868 | 7 | 0.05 |
| 625 | S7_61463 | 7 | 0.06 |
| 626 | S7_213298 | 7 | 0.21 |
| 627 | S7_236267 | 7 | 0.24 |
| 628 | S7_328183 | 7 | 0.33 |
| 629 | S7_497658 | 7 | 0.50 |
| 630 | S7_497704 | 7 | 0.50 |
| 631 | S7_533196 | 7 | 0.53 |
| 632 | S7_533214 | 7 | 0.53 |
| 633 | S7_533544 | 7 | 0.53 |
| 634 | S7_738540 | 7 | 0.74 |
| 635 | S7_743758 | 7 | 0.74 |
| 636 | S7_926618 | 7 | 0.93 |
| 637 | S7_926621 | 7 | 0.93 |
| 638 | S7_2848432 | 7 | 2.85 |
| 639 | S7_3121346 | 7 | 3.12 |
| 640 | S7_3218071 | 7 | 3.22 |
| 641 | S7_5435858 | 7 | 5.44 |
| 642 | S7_17687938 | 7 | 17.69 |
| 643 | S7_18240854 | 7 | 18.24 |
| 644 | S7_19752468 | 7 | 19.75 |
| 645 | S7_21763650 | 7 | 21.76 |
| 646 | S7_22370841 | 7 | 22.37 |
| 647 | S7_22494966 | 7 | 22.49 |
| 648 | S7_23399061 | 7 | 23.40 |
| 649 | S7_23401818 | 7 | 23.40 |
| 650 | S7_25019431 | 7 | 25.02 |
| 651 | S7_27143992 | 7 | 27.14 |
| 652 | S7_27144435 | 7 | 27.14 |
| Supplementary Table 19 (continued) | | | |
| S.No | SNP Marker name | Chromosome .no | Physical position (Mb) |
| 653 | S7_28274256 | 7 | 28.27 |
| 654 | S7_28409850 | 7 | 28.41 |
| 655 | S7_28410168 | 7 | 28.41 |
| 656 | S8_72375 | 8 | 0.07 |
| 657 | S8_138827 | 8 | 0.14 |
| 658 | S8_185506 | 8 | 0.19 |
| 659 | S8_472040 | 8 | 0.47 |
| 660 | S8_663969 | 8 | 0.66 |
| 661 | S8_1637310 | 8 | 1.64 |
| 662 | S8_2772767 | 8 | 2.77 |
| 663 | S8_2816670 | 8 | 2.82 |
| 664 | S8_2890468 | 8 | 2.89 |
| 665 | S8_3092487 | 8 | 3.09 |
| 666 | S8_3229935 | 8 | 3.23 |
| 667 | S8_3301635 | 8 | 3.30 |
| 668 | S8_3681053 | 8 | 3.68 |
| 669 | S8_3741943 | 8 | 3.74 |
| 670 | S8_5239102 | 8 | 5.24 |
| 671 | S8_5700537 | 8 | 5.70 |
| 672 | S8_5780637 | 8 | 5.78 |
| 673 | S8_5811019 | 8 | 5.81 |
| 674 | S8_6274619 | 8 | 6.27 |
| 675 | S8_7166061 | 8 | 7.17 |
| 676 | S8_7660857 | 8 | 7.66 |
| 677 | S8_7783462 | 8 | 7.78 |
| 678 | S8_8286821 | 8 | 8.29 |
| 679 | S8_9071396 | 8 | 9.07 |
| 680 | S8_9629078 | 8 | 9.63 |
| 681 | S8_9780177 | 8 | 9.78 |
| 682 | S8_10025829 | 8 | 10.03 |
| 683 | S8_10678527 | 8 | 10.68 |
| 684 | S8_11781764 | 8 | 11.78 |
| 685 | S8_12267313 | 8 | 12.27 |
| 686 | S8_13422920 | 8 | 13.42 |
| 687 | S8_13739663 | 8 | 13.74 |
| 688 | S8_14327460 | 8 | 14.33 |
| 689 | S8_14930927 | 8 | 14.93 |
| 690 | S8_16383401 | 8 | 16.38 |
| 691 | S8_16469851 | 8 | 16.47 |
| 692 | S8_17646816 | 8 | 17.65 |
| 693 | S8_17828746 | 8 | 17.83 |
| 694 | S8_18446977 | 8 | 18.45 |
| 695 | S8_19719271 | 8 | 19.72 |
| 696 | S8_20144837 | 8 | 20.14 |
| 697 | S8_20389809 | 8 | 20.39 |
| 698 | S8_20433459 | 8 | 20.43 |
| 699 | S8_20649459 | 8 | 20.65 |
| 700 | S8_21539311 | 8 | 21.54 |
| 701 | S8_21834730 | 8 | 21.83 |
| 702 | S8_22924035 | 8 | 22.92 |
| Supplementary Table 19 (continued) | | | |
| S.No | SNP Marker name | Chromosome .no | Physical position (Mb) |
| 703 | S8_22924077 | 8 | 22.92 |
| 704 | S8_22924085 | 8 | 22.92 |
| 705 | S8_25532723 | 8 | 25.53 |
| 706 | S8_26422710 | 8 | 26.42 |
| 707 | S8_26882452 | 8 | 26.88 |
| 708 | S8_26977980 | 8 | 26.98 |
| 709 | S8_27037428 | 8 | 27.04 |
| 710 | S8_27176044 | 8 | 27.18 |
| 711 | S8_27420356 | 8 | 27.42 |
| 712 | S8_27641077 | 8 | 27.64 |
| 713 | S8_27746905 | 8 | 27.75 |
| 714 | S8_27919024 | 8 | 27.92 |
| 715 | S8_28426756 | 8 | 28.43 |
| 716 | S9_412246 | 9 | 0.41 |
| 717 | S9_1170092 | 9 | 1.17 |
| 718 | S9_1863532 | 9 | 1.86 |
| 719 | S9_1863559 | 9 | 1.86 |
| 720 | S9_3370701 | 9 | 3.37 |
| 721 | S9_3801844 | 9 | 3.80 |
| 722 | S9_5038692 | 9 | 5.04 |
| 723 | S9_5140423 | 9 | 5.14 |
| 724 | S9_5432884 | 9 | 5.43 |
| 725 | S9_5645317 | 9 | 5.65 |
| 726 | S9_5871856 | 9 | 5.87 |
| 727 | S9_5920442 | 9 | 5.92 |
| 728 | S9_6332889 | 9 | 6.33 |
| 729 | S9_6332923 | 9 | 6.33 |
| 730 | S9_6746282 | 9 | 6.75 |
| 731 | S9_6746570 | 9 | 6.75 |
| 732 | S9_7408580 | 9 | 7.41 |
| 733 | S9_7508836 | 9 | 7.51 |
| 734 | S9_7508872 | 9 | 7.51 |
| 735 | S9_7852474 | 9 | 7.85 |
| 736 | S9_7852553 | 9 | 7.85 |
| 737 | S9_8512396 | 9 | 8.51 |
| 738 | S9_8520088 | 9 | 8.52 |
| 739 | S9_8520107 | 9 | 8.52 |
| 740 | S9_8568722 | 9 | 8.57 |
| 741 | S9_8891118 | 9 | 8.89 |
| 742 | S9_8891120 | 9 | 8.89 |
| 743 | S9_8891125 | 9 | 8.89 |
| 744 | S9_8891134 | 9 | 8.89 |
| 745 | S9_9188125 | 9 | 9.19 |
| 746 | S9_10329832 | 9 | 10.33 |
| 747 | S9_10524448 | 9 | 10.52 |
| 748 | S9_11781837 | 9 | 11.78 |
| 749 | S9_11782003 | 9 | 11.78 |
| 750 | S9_12370174 | 9 | 12.37 |
| 751 | S9_12915373 | 9 | 12.92 |
| 752 | S9_13456275 | 9 | 13.46 |
| Supplementary Table 19 (continued) | | | |
| S.No | SNP Marker name | Chromosome .no | Physical position (Mb) |
| 753 | S9_13456282 | 9 | 13.46 |
| 754 | S9_13456299 | 9 | 13.46 |
| 755 | S9_15438410 | 9 | 15.44 |
| 756 | S9_15829836 | 9 | 15.83 |
| 757 | S9_15829837 | 9 | 15.83 |
| 758 | S9_16588200 | 9 | 16.59 |
| 759 | S9_18312728 | 9 | 18.31 |
| 760 | S9_20095752 | 9 | 20.10 |
| 761 | S9_20713606 | 9 | 20.71 |
| 762 | S9_21502125 | 9 | 21.50 |
| 763 | S9_22015050 | 9 | 22.02 |
| 764 | S9_22015057 | 9 | 22.02 |
| 765 | S9_22015062 | 9 | 22.02 |
| 766 | S9_22015091 | 9 | 22.02 |
| 767 | S9_22567431 | 9 | 22.57 |
| 768 | S9_22650237 | 9 | 22.65 |
| 769 | S9_22650418 | 9 | 22.65 |
| 770 | S9_22650427 | 9 | 22.65 |
| 771 | S9_22650438 | 9 | 22.65 |
| 772 | S9_22650443 | 9 | 22.65 |
| 773 | S10_301759 | 10 | 0.30 |
| 774 | S10_430683 | 10 | 0.43 |
| 775 | S10_432687 | 10 | 0.43 |
| 776 | S10_472488 | 10 | 0.47 |
| 777 | S10_502193 | 10 | 0.50 |
| 778 | S10_683250 | 10 | 0.68 |
| 779 | S10_707340 | 10 | 0.71 |
| 780 | S10_977414 | 10 | 0.98 |
| 781 | S10_1050010 | 10 | 1.05 |
| 782 | S10_1469464 | 10 | 1.47 |
| 783 | S10_1674710 | 10 | 1.67 |
| 784 | S10_5443002 | 10 | 5.44 |
| 785 | S10_5809390 | 10 | 5.81 |
| 786 | S10_5828161 | 10 | 5.83 |
| 787 | S10_5930103 | 10 | 5.93 |
| 788 | S10_5930105 | 10 | 5.93 |
| 789 | S10_6017408 | 10 | 6.02 |
| 790 | S10_6017573 | 10 | 6.02 |
| 791 | S10_6019584 | 10 | 6.02 |
| 792 | S10_6029095 | 10 | 6.03 |
| 793 | S10_6259967 | 10 | 6.26 |
| 794 | S10_6260126 | 10 | 6.26 |
| 795 | S10_7540378 | 10 | 7.54 |
| 796 | S10_8258652 | 10 | 8.26 |
| 797 | S10_8258685 | 10 | 8.26 |
| 798 | S10_9233857 | 10 | 9.23 |
| 799 | S10_9627559 | 10 | 9.63 |
| 800 | S10_9627906 | 10 | 9.63 |
| 801 | S10_9865313 | 10 | 9.87 |
| 802 | S10_10014597 | 10 | 10.01 |
| Supplementary Table 19 (continued) | | | |
| S.No | SNP Marker name | Chromosome .no | Physical position (Mb) |
| 803 | S10_10110674 | 10 | 10.11 |
| 804 | S10_10859476 | 10 | 10.86 |
| 805 | S10_11129803 | 10 | 11.13 |
| 806 | S10_14874544 | 10 | 14.87 |
| 807 | S10_15445068 | 10 | 15.45 |
| 808 | S10_16152054 | 10 | 16.15 |
| 809 | S10_17420023 | 10 | 17.42 |
| 810 | S10_18828909 | 10 | 18.83 |
| 811 | S10_19890971 | 10 | 19.89 |
| 812 | S10_20266477 | 10 | 20.27 |
| 813 | S10_20693837 | 10 | 20.69 |
| 814 | S10_20820442 | 10 | 20.82 |
| 815 | S10_20902358 | 10 | 20.90 |
| 816 | S10_21133925 | 10 | 21.13 |
| 817 | S10_21203353 | 10 | 21.20 |
| 818 | S10_21323384 | 10 | 21.32 |
| 819 | S10_21449027 | 10 | 21.45 |
| 820 | S10_21480940 | 10 | 21.48 |
| 821 | S10_21656019 | 10 | 21.66 |
| 822 | S10_21803569 | 10 | 21.80 |
| 823 | S10_22091320 | 10 | 22.09 |
| 824 | S10_22169744 | 10 | 22.17 |
| 825 | S10_22776834 | 10 | 22.78 |
| 826 | S10_23089807 | 10 | 23.09 |
| 827 | S11_244950 | 11 | 0.24 |
| 828 | S11_668438 | 11 | 0.67 |
| 829 | S11_795737 | 11 | 0.80 |
| 830 | S11_848092 | 11 | 0.85 |
| 831 | S11_848123 | 11 | 0.85 |
| 832 | S11_965658 | 11 | 0.97 |
| 833 | S11_965670 | 11 | 0.97 |
| 834 | S11_1125465 | 11 | 1.13 |
| 835 | S11_1125501 | 11 | 1.13 |
| 836 | S11_1126003 | 11 | 1.13 |
| 837 | S11_1206915 | 11 | 1.21 |
| 838 | S11_1206968 | 11 | 1.21 |
| 839 | S11_1639897 | 11 | 1.64 |
| 840 | S11_1639898 | 11 | 1.64 |
| 841 | S11_1639909 | 11 | 1.64 |
| 842 | S11_1639931 | 11 | 1.64 |
| 843 | S11_1640318 | 11 | 1.64 |
| 844 | S11_1704633 | 11 | 1.70 |
| 845 | S11_1937049 | 11 | 1.94 |
| 846 | S11_2426246 | 11 | 2.43 |
| 847 | S11_2843111 | 11 | 2.84 |
| 848 | S11_2843488 | 11 | 2.84 |
| 849 | S11_2843489 | 11 | 2.84 |
| 850 | S11_5350244 | 11 | 5.35 |
| 851 | S11_9766936 | 11 | 9.77 |
| 852 | S11_9766937 | 11 | 9.77 |
| Supplementary Table 19 (continued) | | | |
| S.No | SNP Marker name | Chromosome .no | Physical position (Mb) |
| 853 | S11_9766961 | 11 | 9.77 |
| 854 | S11_10912910 | 11 | 10.91 |
| 855 | S11_10918301 | 11 | 10.92 |
| 856 | S11_11436519 | 11 | 11.44 |
| 857 | S11_11436520 | 11 | 11.44 |
| 858 | S11_11436522 | 11 | 11.44 |
| 859 | S11_11436523 | 11 | 11.44 |
| 860 | S11_11779858 | 11 | 11.78 |
| 861 | S11_11938423 | 11 | 11.94 |
| 862 | S11_11938455 | 11 | 11.94 |
| 863 | S11_11938474 | 11 | 11.94 |
| 864 | S11_12077123 | 11 | 12.08 |
| 865 | S11_12852354 | 11 | 12.85 |
| 866 | S11_14322941 | 11 | 14.32 |
| 867 | S11_14429769 | 11 | 14.43 |
| 868 | S11_14555418 | 11 | 14.56 |
| 869 | S11_14787010 | 11 | 14.79 |
| 870 | S11_15005465 | 11 | 15.01 |
| 871 | S11_15462589 | 11 | 15.46 |
| 872 | S11_15562961 | 11 | 15.56 |
| 873 | S11_16200411 | 11 | 16.20 |
| 874 | S11_16223250 | 11 | 16.22 |
| 875 | S11_16223255 | 11 | 16.22 |
| 876 | S11_16282411 | 11 | 16.28 |
| 877 | S11_16422103 | 11 | 16.42 |
| 878 | S11_16511045 | 11 | 16.51 |
| 879 | S11_16511087 | 11 | 16.51 |
| 880 | S11_16560027 | 11 | 16.56 |
| 881 | S11_16560301 | 11 | 16.56 |
| 882 | S11_16752173 | 11 | 16.75 |
| 883 | S11_16752178 | 11 | 16.75 |
| 884 | S11_16779579 | 11 | 16.78 |
| 885 | S11_16849388 | 11 | 16.85 |
| 886 | S11_16849389 | 11 | 16.85 |
| 887 | S11_16910367 | 11 | 16.91 |
| 888 | S11_17575511 | 11 | 17.58 |
| 889 | S11_17575691 | 11 | 17.58 |
| 890 | S11_17575694 | 11 | 17.58 |
| 891 | S11_17575701 | 11 | 17.58 |
| 892 | S11_17575725 | 11 | 17.58 |
| 893 | S11_17582613 | 11 | 17.58 |
| 894 | S11_18426628 | 11 | 18.43 |
| 895 | S11_18427065 | 11 | 18.43 |
| 896 | S11_18428420 | 11 | 18.43 |
| 897 | S11_18428455 | 11 | 18.43 |
| 898 | S11_18431520 | 11 | 18.43 |
| 899 | S11_18431537 | 11 | 18.43 |
| 900 | S11_18431540 | 11 | 18.43 |
| 901 | S11_18431570 | 11 | 18.43 |
| 902 | S11_18562144 | 11 | 18.56 |
| Supplementary Table 19 (continued) | | | |
| S.No | SNP Marker name | Chromosome .no | Physical position (Mb) |
| 903 | S11_19808318 | 11 | 19.81 |
| 904 | S11_19808328 | 11 | 19.81 |
| 905 | S11_19808341 | 11 | 19.81 |
| 906 | S11_20770136 | 11 | 20.77 |
| 907 | S11_20770188 | 11 | 20.77 |
| 908 | S11_20770196 | 11 | 20.77 |
| 909 | S11_21409245 | 11 | 21.41 |
| 910 | S11_21463003 | 11 | 21.46 |
| 911 | S11_21463006 | 11 | 21.46 |
| 912 | S11_21463013 | 11 | 21.46 |
| 913 | S11_21469989 | 11 | 21.47 |
| 914 | S11_22012641 | 11 | 22.01 |
| 915 | S11_22154203 | 11 | 22.15 |
| 916 | S11_22461719 | 11 | 22.46 |
| 917 | S11_22461755 | 11 | 22.46 |
| 918 | S11_23405332 | 11 | 23.41 |
| 919 | S11_23405338 | 11 | 23.41 |
| 920 | S11_23405348 | 11 | 23.41 |
| 921 | S11_23405384 | 11 | 23.41 |
| 922 | S11_23405396 | 11 | 23.41 |
| 923 | S11_23405445 | 11 | 23.41 |
| 924 | S11_23405450 | 11 | 23.41 |
| 925 | S11_23898413 | 11 | 23.90 |
| 926 | S11_23986628 | 11 | 23.99 |
| 927 | S11_23986837 | 11 | 23.99 |
| 928 | S11_24162971 | 11 | 24.16 |
| 929 | S11_25538899 | 11 | 25.54 |
| 930 | S11_25634410 | 11 | 25.63 |
| 931 | S11_25634411 | 11 | 25.63 |
| 932 | S11_25721656 | 11 | 25.72 |
| 933 | S11_25725490 | 11 | 25.73 |
| 934 | S11_25725502 | 11 | 25.73 |
| 935 | S11_25725720 | 11 | 25.73 |
| 936 | S11_25854146 | 11 | 25.85 |
| 937 | S11_25946590 | 11 | 25.95 |
| 938 | S11_26261107 | 11 | 26.26 |
| 939 | S11_26261114 | 11 | 26.26 |
| 940 | S11_26380086 | 11 | 26.38 |
| 941 | S11_26435712 | 11 | 26.44 |
| 942 | S11_26529099 | 11 | 26.53 |
| 943 | S11_26529100 | 11 | 26.53 |
| 944 | S11_26529140 | 11 | 26.53 |
| 945 | S11_26596519 | 11 | 26.60 |
| 946 | S11_26916668 | 11 | 26.92 |
| 947 | S11_27181085 | 11 | 27.18 |
| 948 | S11_27181098 | 11 | 27.18 |
| 949 | S11_27181110 | 11 | 27.18 |
| 950 | S11_27181322 | 11 | 27.18 |
| 951 | S11_27207653 | 11 | 27.21 |
| 952 | S11_27213592 | 11 | 27.21 |
| Supplementary Table 19 (continued) | | | |
| S.No | SNP Marker name | Chromosome .no | Physical position (Mb) |
| 953 | S11_27213616 | 11 | 27.21 |
| 954 | S11_27213755 | 11 | 27.21 |
| 955 | S11_27213851 | 11 | 27.21 |
| 956 | S11_27213852 | 11 | 27.21 |
| 957 | S11_27234782 | 11 | 27.23 |
| 958 | S11_27483001 | 11 | 27.48 |
| 959 | S11_27494377 | 11 | 27.49 |
| 960 | S11_27494387 | 11 | 27.49 |
| 961 | S11_27494406 | 11 | 27.49 |
| 962 | S11_27788787 | 11 | 27.79 |
| 963 | S11_27817978 | 11 | 27.82 |
| 964 | S11_27818463 | 11 | 27.82 |
| 965 | S11_27831425 | 11 | 27.83 |
| 966 | S11_27831441 | 11 | 27.83 |
| 967 | S11_28340561 | 11 | 28.34 |
| 968 | S11_28340579 | 11 | 28.34 |
| 969 | S11_28340587 | 11 | 28.34 |
| 970 | S11_28454253 | 11 | 28.45 |
| 971 | S11_28454286 | 11 | 28.45 |
| 972 | S11_28454300 | 11 | 28.45 |
| 973 | S11_28629598 | 11 | 28.63 |
| 974 | S11_28806774 | 11 | 28.81 |
| 975 | S11_28832752 | 11 | 28.83 |
| 976 | S11_28832902 | 11 | 28.83 |
| 977 | S11_28872149 | 11 | 28.87 |
| 978 | S12_443258 | 12 | 0.44 |
| 979 | S12_1576092 | 12 | 1.58 |
| 980 | S12_1809337 | 12 | 1.81 |
| 981 | S12_5612260 | 12 | 5.61 |
| 982 | S12_5751186 | 12 | 5.75 |
| 983 | S12_6584008 | 12 | 6.58 |
| 984 | S12_7116866 | 12 | 7.12 |
| 985 | S12_7326732 | 12 | 7.33 |
| 986 | S12_7918127 | 12 | 7.92 |
| 987 | S12_8961198 | 12 | 8.96 |
| 988 | S12_8961419 | 12 | 8.96 |
| 989 | S12_9180371 | 12 | 9.18 |
| 990 | S12_9478077 | 12 | 9.48 |
| 991 | S12_9568767 | 12 | 9.57 |
| 992 | S12_9788283 | 12 | 9.79 |
| 993 | S12_9788284 | 12 | 9.79 |
| 994 | S12_9896101 | 12 | 9.90 |
| 995 | S12_10019290 | 12 | 10.02 |
| 996 | S12_10084657 | 12 | 10.08 |
| 997 | S12_10084776 | 12 | 10.08 |
| 998 | S12_10760359 | 12 | 10.76 |
| 999 | S12_10760600 | 12 | 10.76 |
| 1000 | S12_10872658 | 12 | 10.87 |
| 1001 | S12_11870243 | 12 | 11.87 |
| 1002 | S12_12970403 | 12 | 12.97 |
| Supplementary Table 19 (continued) | | | |
| S.No | SNP Marker name | Chromosome .no | Physical position (Mb) |
| 1003 | S12_13115273 | 12 | 13.12 |
| 1004 | S12_13190499 | 12 | 13.19 |
| 1005 | S12_13271524 | 12 | 13.27 |
| 1006 | S12_13297545 | 12 | 13.30 |
| 1007 | S12_13304179 | 12 | 13.30 |
| 1008 | S12_13304208 | 12 | 13.30 |
| 1009 | S12_13304211 | 12 | 13.30 |
| 1010 | S12_13304213 | 12 | 13.30 |
| 1011 | S12_14867709 | 12 | 14.87 |
| 1012 | S12_14963329 | 12 | 14.96 |
| 1013 | S12_14963361 | 12 | 14.96 |
| 1014 | S12_14963535 | 12 | 14.96 |
| 1015 | S12_14963553 | 12 | 14.96 |
| 1016 | S12_15140411 | 12 | 15.14 |
| 1017 | S12_15433199 | 12 | 15.43 |
| 1018 | S12_15433200 | 12 | 15.43 |
| 1019 | S12_17478598 | 12 | 17.48 |
| 1020 | S12_17564366 | 12 | 17.56 |
| 1021 | S12_17920428 | 12 | 17.92 |
| 1022 | S12_17960746 | 12 | 17.96 |
| 1023 | S12_18004640 | 12 | 18.00 |
| 1024 | S12_18005069 | 12 | 18.01 |
| 1025 | S12_18099661 | 12 | 18.10 |
| 1026 | S12_18109176 | 12 | 18.11 |
| 1027 | S12_18109187 | 12 | 18.11 |
| 1028 | S12_18329036 | 12 | 18.33 |
| 1029 | S12_18344256 | 12 | 18.34 |
| 1030 | S12_18759117 | 12 | 18.76 |
| 1031 | S12_19139100 | 12 | 19.14 |
| 1032 | S12_19261269 | 12 | 19.26 |
| 1033 | S12_19261271 | 12 | 19.26 |
| 1034 | S12_19476809 | 12 | 19.48 |
| 1035 | S12_19537089 | 12 | 19.54 |
| 1036 | S12_19598014 | 12 | 19.60 |
| 1037 | S12_19835409 | 12 | 19.84 |
| 1038 | S12_19897344 | 12 | 19.90 |
| 1039 | S12_20086502 | 12 | 20.09 |
| 1040 | S12_20086511 | 12 | 20.09 |
| 1041 | S12_20802707 | 12 | 20.80 |
| 1042 | S12_21221887 | 12 | 21.22 |
| 1043 | S12_21222252 | 12 | 21.22 |
| 1044 | S12_21791447 | 12 | 21.79 |
| 1045 | S12_21791497 | 12 | 21.79 |
| 1046 | S12_22217190 | 12 | 22.22 |
| 1047 | S12_22418443 | 12 | 22.42 |
| 1048 | S12_22461106 | 12 | 22.46 |
| 1049 | S12_22461145 | 12 | 22.46 |
| 1050 | S12_22603668 | 12 | 22.60 |
| 1051 | S12_23272380 | 12 | 23.27 |
| 1052 | S12_23581612 | 12 | 23.58 |
| Supplementary Table 19 (continued) | | | |
| S.No | SNP Marker name | Chromosome .no | Physical position (Mb) |
| 1053 | S12_23608042 | 12 | 23.61 |
| 1054 | S12_23608074 | 12 | 23.61 |
| 1055 | S12_23608293 | 12 | 23.61 |
| 1056 | S12_23608329 | 12 | 23.61 |
| 1057 | S12_23608334 | 12 | 23.61 |
| 1058 | S12_23893583 | 12 | 23.89 |
| 1059 | S12_23893686 | 12 | 23.89 |
| 1060 | S12_24256694 | 12 | 24.26 |
| 1061 | S12_24281348 | 12 | 24.28 |
| 1062 | S12_24319031 | 12 | 24.32 |
| 1063 | S12_24349772 | 12 | 24.35 |
| 1064 | S12_24480049 | 12 | 24.48 |
| 1065 | S12_24509801 | 12 | 24.51 |
| 1066 | S12_26369343 | 12 | 26.37 |
| 1067 | S12_26624527 | 12 | 26.62 |
| 1068 | S12_26804341 | 12 | 26.80 |
| 1069 | S12_26804360 | 12 | 26.80 |
| 1070 | S12_26804366 | 12 | 26.80 |
| 1071 | S12_26804389 | 12 | 26.80 |
| 1072 | S12_26810779 | 12 | 26.81 |
| 1073 | S12_26810808 | 12 | 26.81 |
| 1074 | S12_26810811 | 12 | 26.81 |
| 1075 | S12_26812180 | 12 | 26.81 |
| 1076 | S12_26812203 | 12 | 26.81 |
| 1077 | S12_26812212 | 12 | 26.81 |
| 1078 | S12_26812227 | 12 | 26.81 |
| 1079 | S12_27349817 | 12 | 27.35 |
| 1080 | S12_27349819 | 12 | 27.35 |
| 1081 | S12_27365532 | 12 | 27.37 |
| 1082 | S12_27381197 | 12 | 27.38 |

Supplementary Figure 1: Frequency distribution histograms of the means of 105 RIL populations for traits DFF, YLD, GP, FGP, TGW, PW, PH, FLL, FLW, PT, PL, BM. Increasing order of mean values is shown on X-axis and frequency on Y-axis


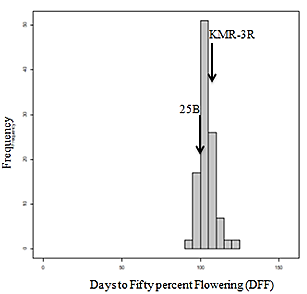

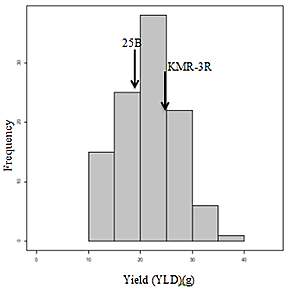

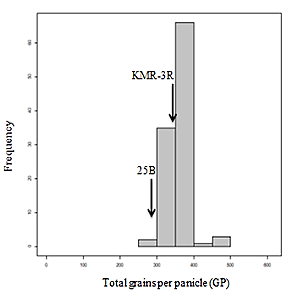

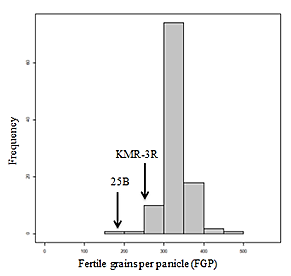

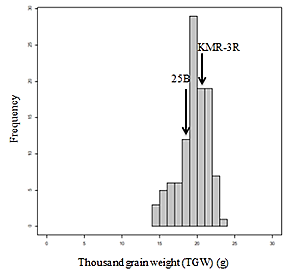

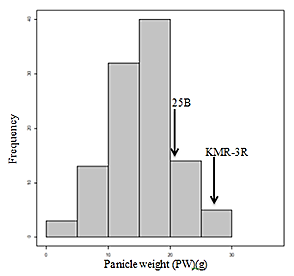

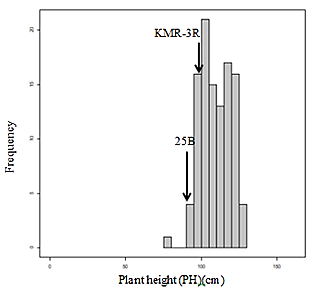

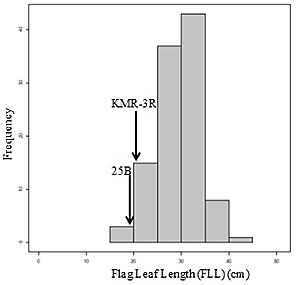

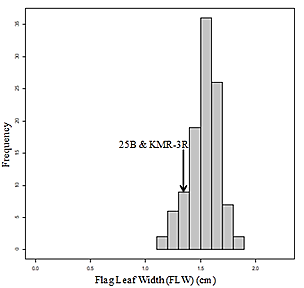

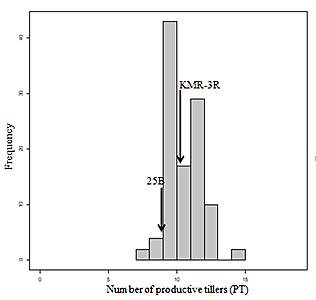

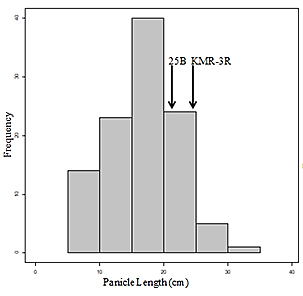

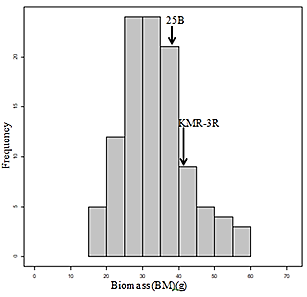


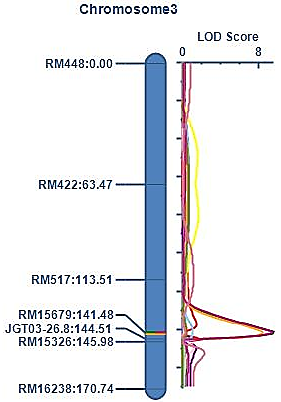

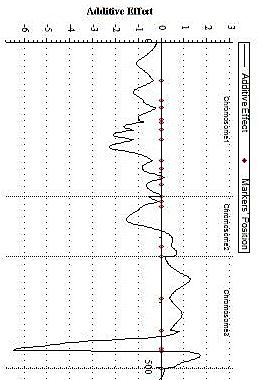

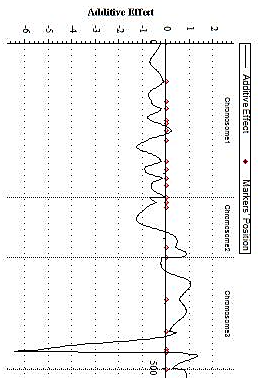


Additive effect of *qPW3-1*

**(II)**

**(I)**

Additive effect of *qYLD3-1* and *qPL3-1*


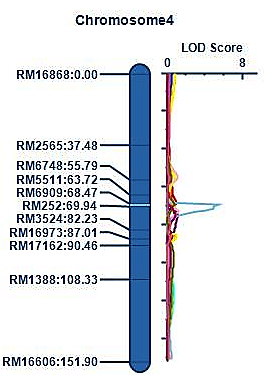

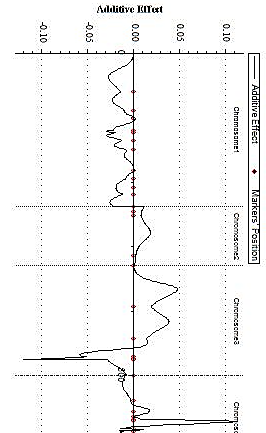

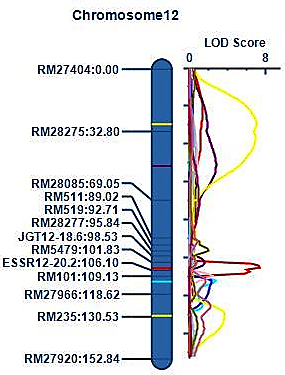

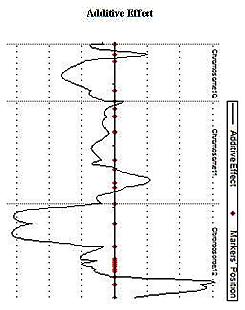


**(IV)**

**(III)**

Additive effect of *qPH12-1*

Additive effect of *qFLW4-1*

Supplementary Figure 2: Additive effect of the yield associated major effect QTLs discovered on chromosome 3 (indicated by asterisk) for the traits total grain yield/plant (g) (YLD; 2(I)), panicle length (cm)(PL; 2(I)) and panicle weight (g)(PW; 2(II)). The additive effect of these QTLs is indicated by an arrow showing their direction from KMR-3R. Also, significant major effect QTLs indicated by asterisk (*) on chromosome 4 & 12 for traits flag leaf width (FLW; 2(III)) & plant height (PH; 2(IV)), respectively, is shown. Additive effect of these QTLs is indicated by additive effect peaks (indicated by black arrow). The inheritance of these QTLs is from the recurrent parent, IR58025A.


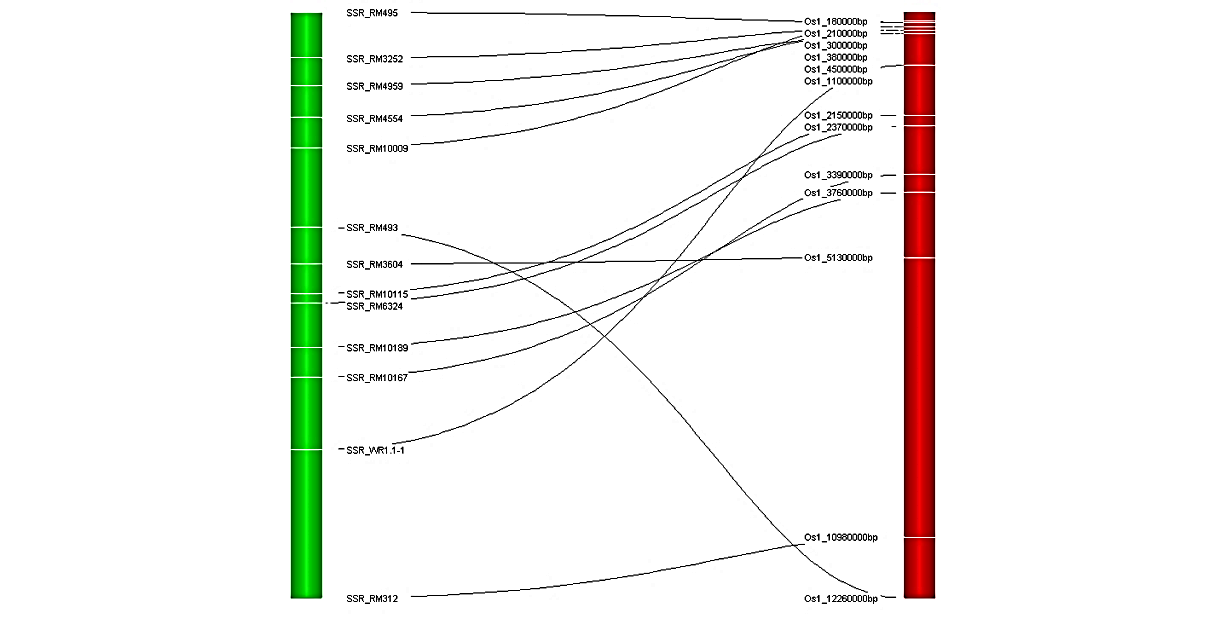


**Genetic map *Os1***


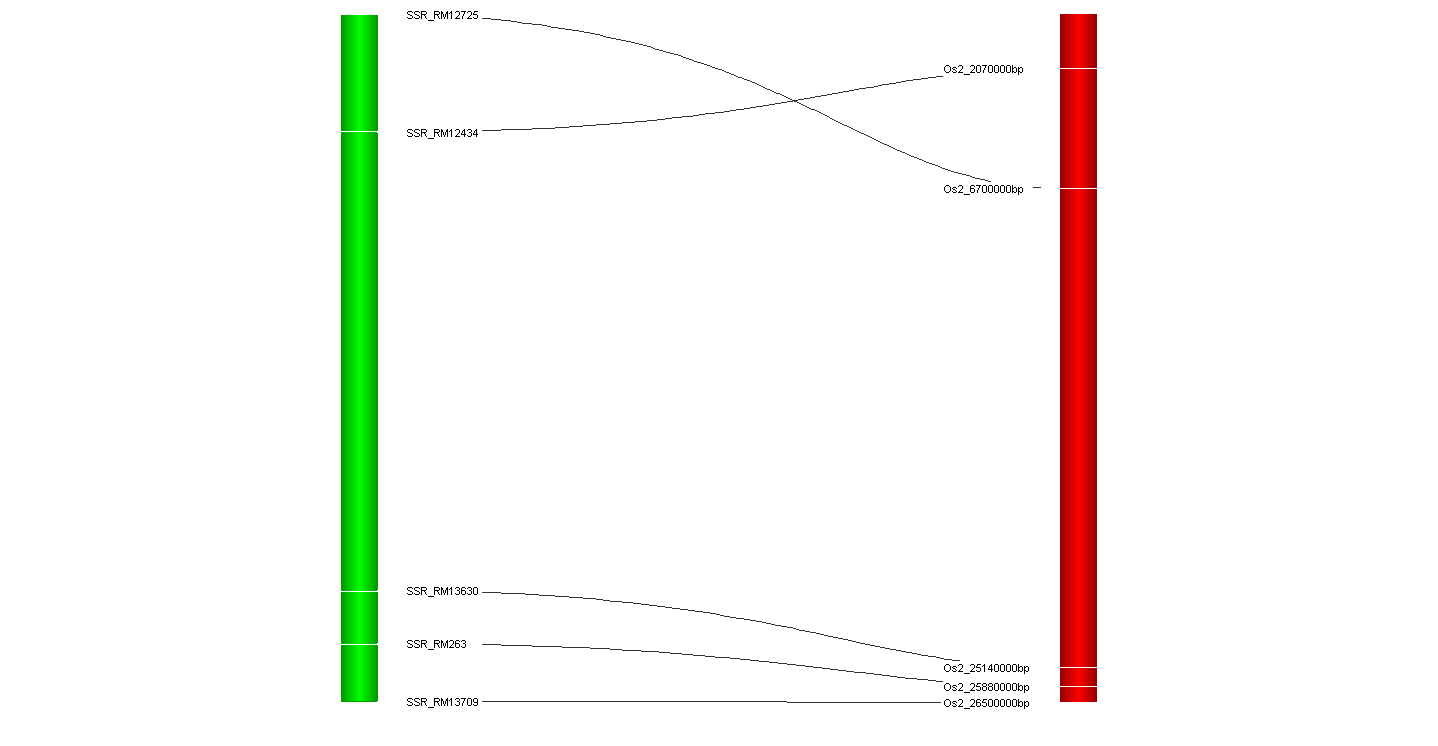


**Physical map *Os1***

**Genetic map *Os2***

**Physical map *Os2***


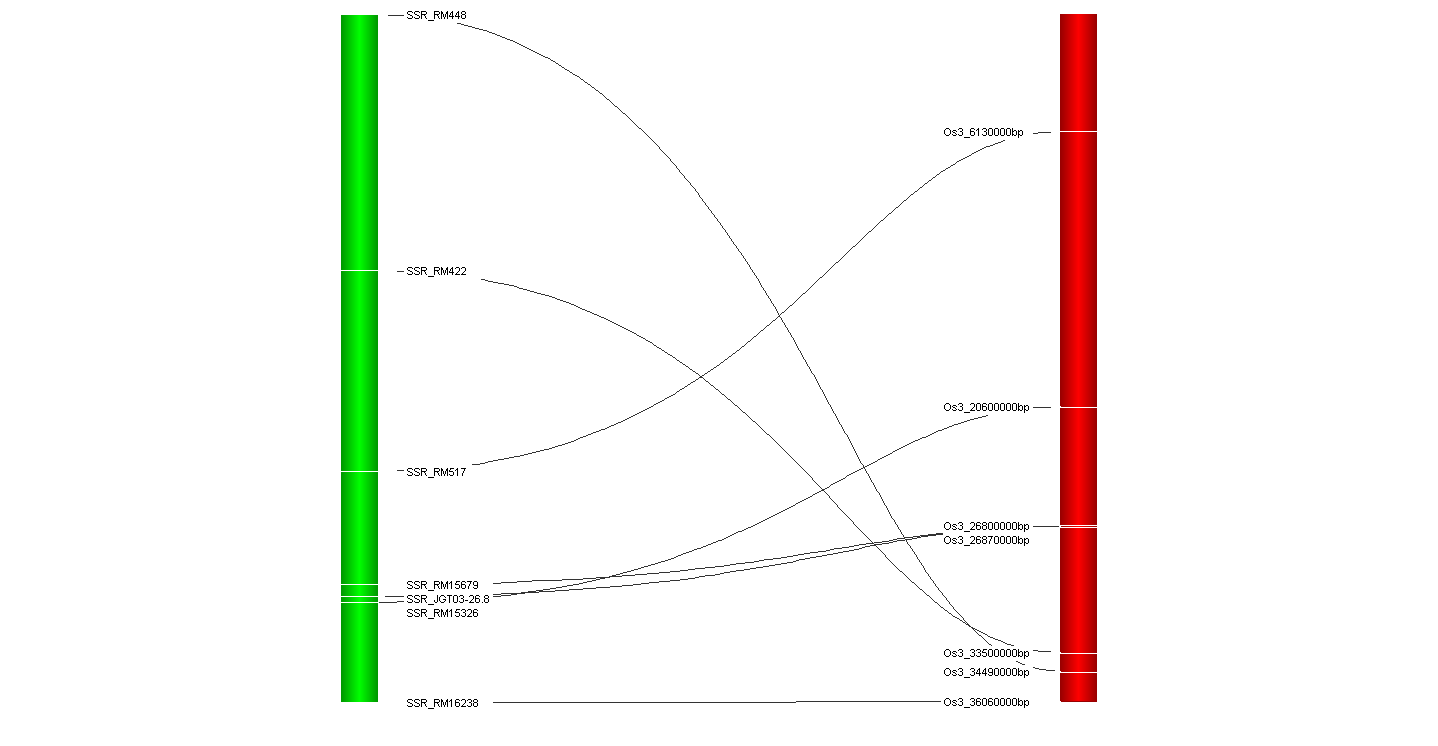


**Genetic map *Os3***


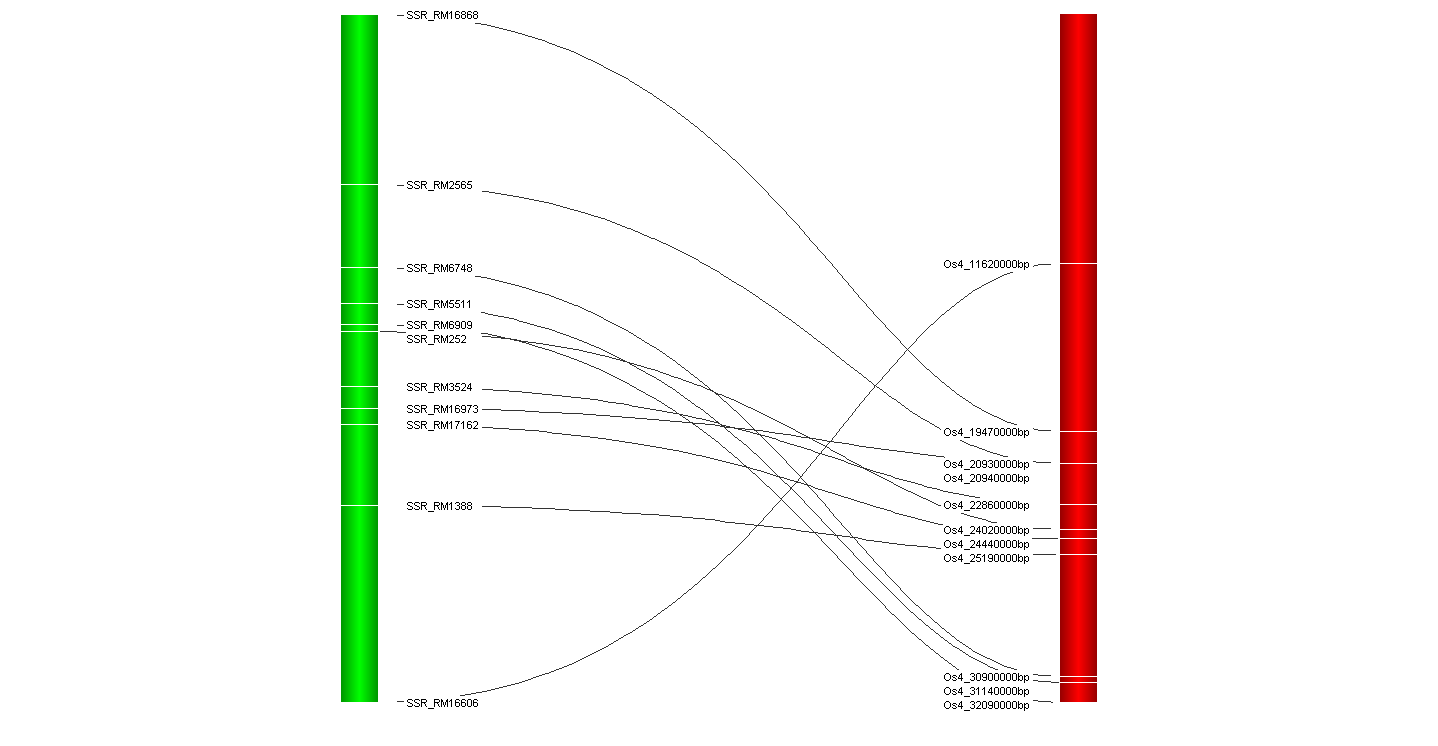


**Physical map *Os3***

**Genetic map *Os4***

**Physical map *Os4***


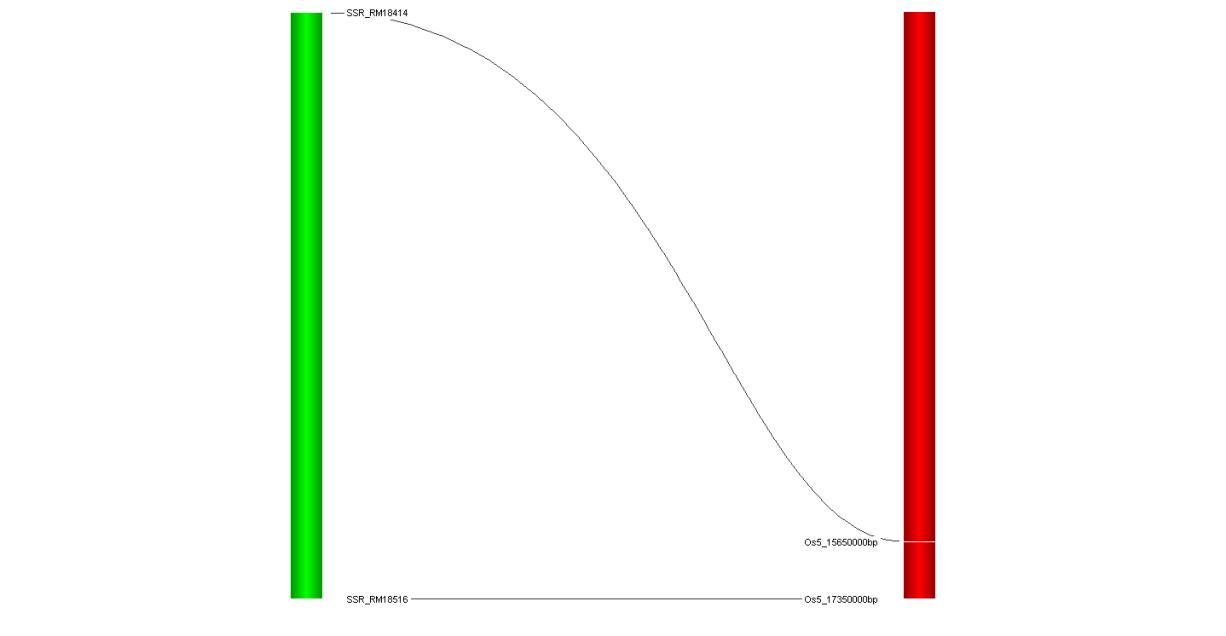


**Genetic map *Os5***

**Physical map *Os5***


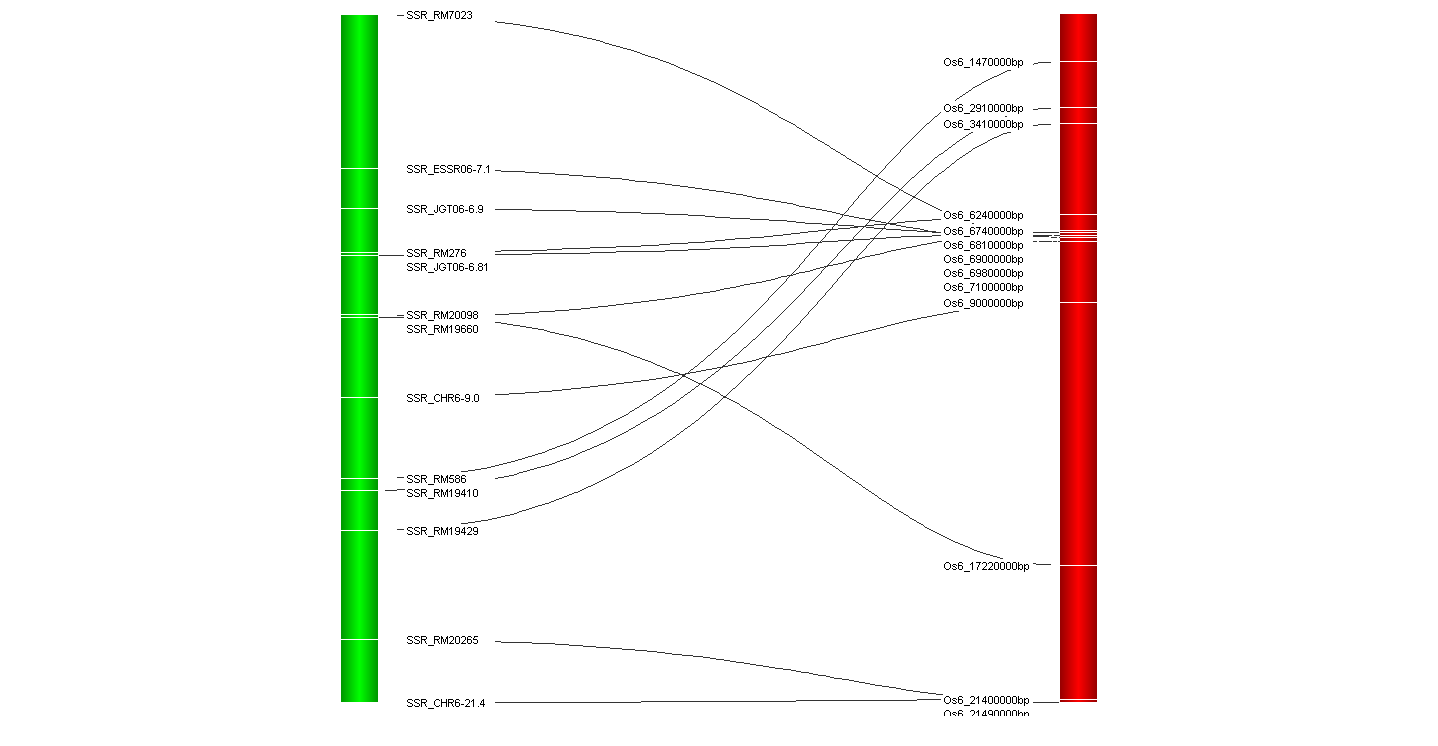


**Genetic map *Os6***

**Physical map *Os6***

**Physical map *Os7***


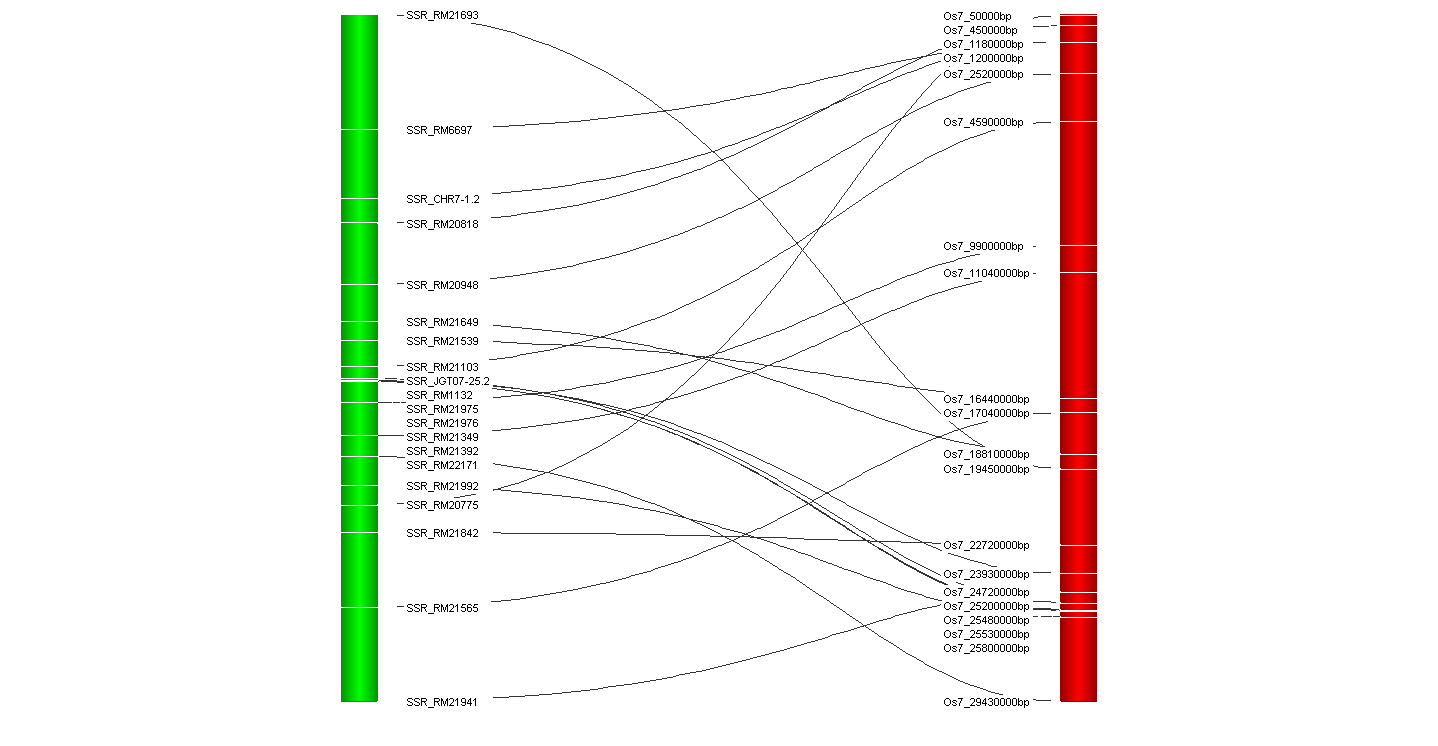


**Genetic map *Os7***


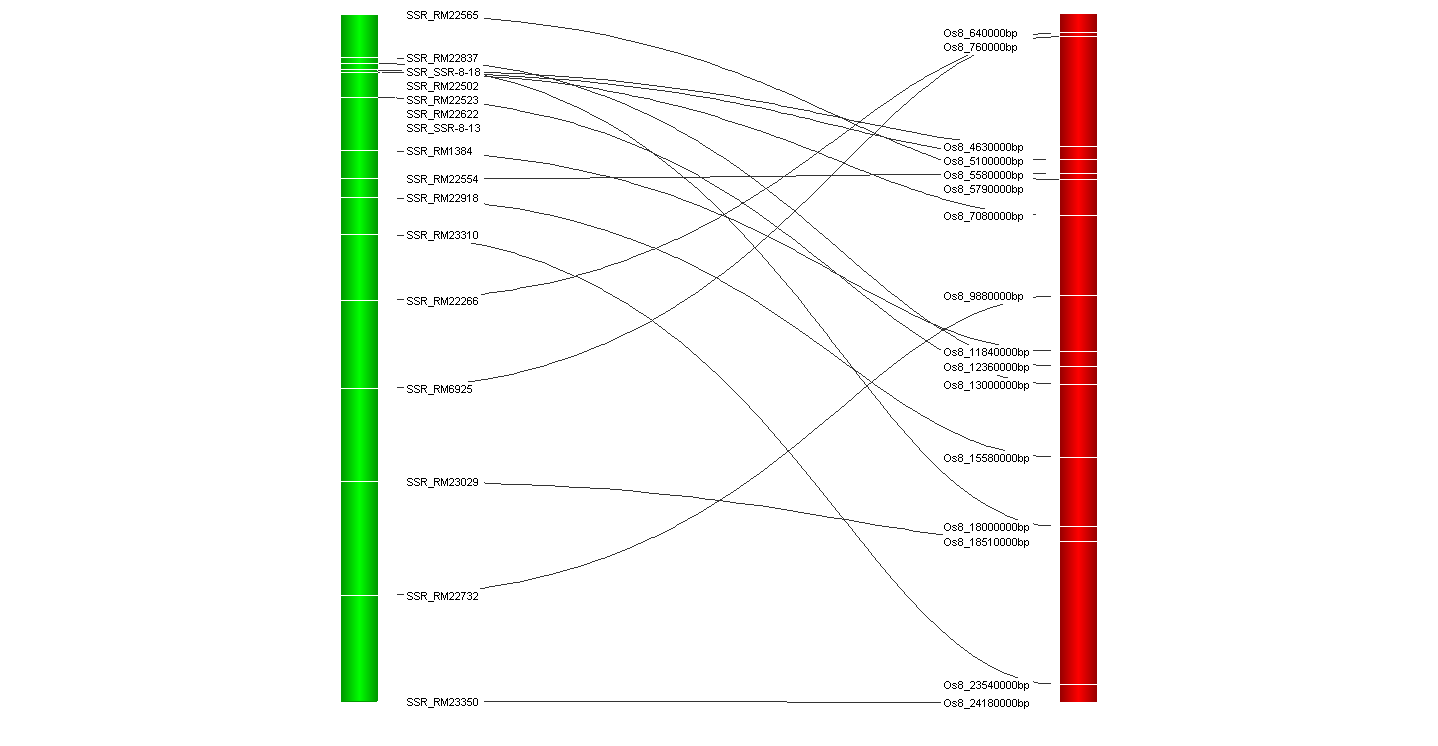


**Genetic map *Os8***

**Physical map *Os8***


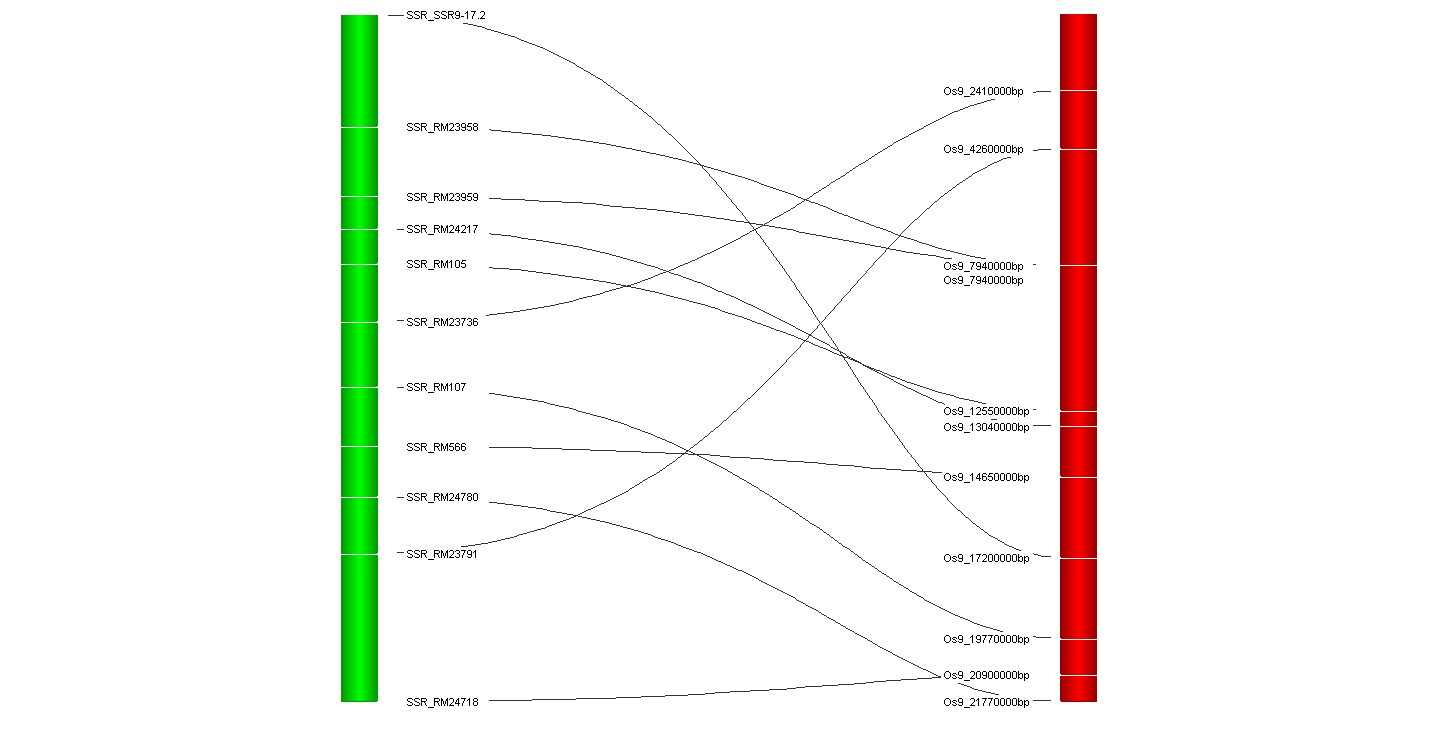


**Genetic map *Os9***

**Physical map *Os9***


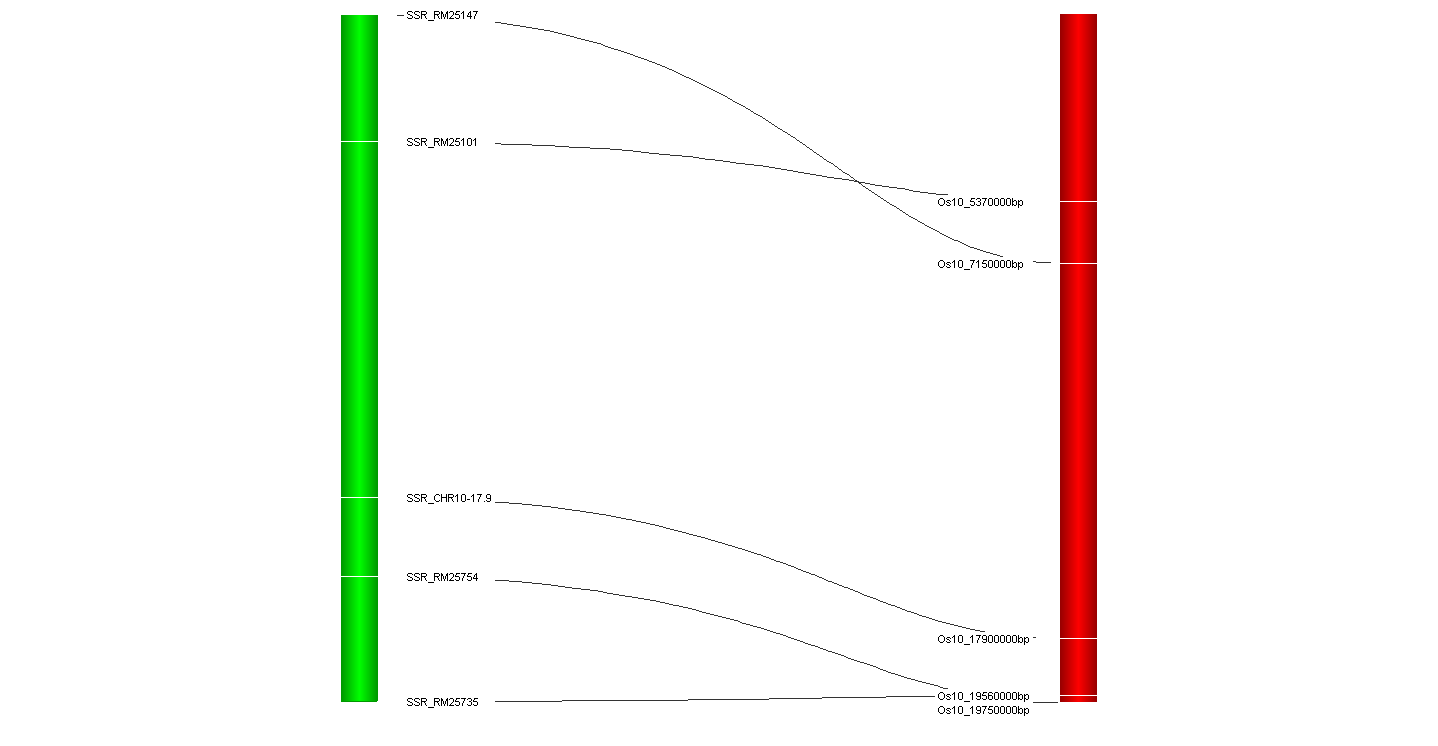


**Genetic map *Os10***

**Physical map *Os10***


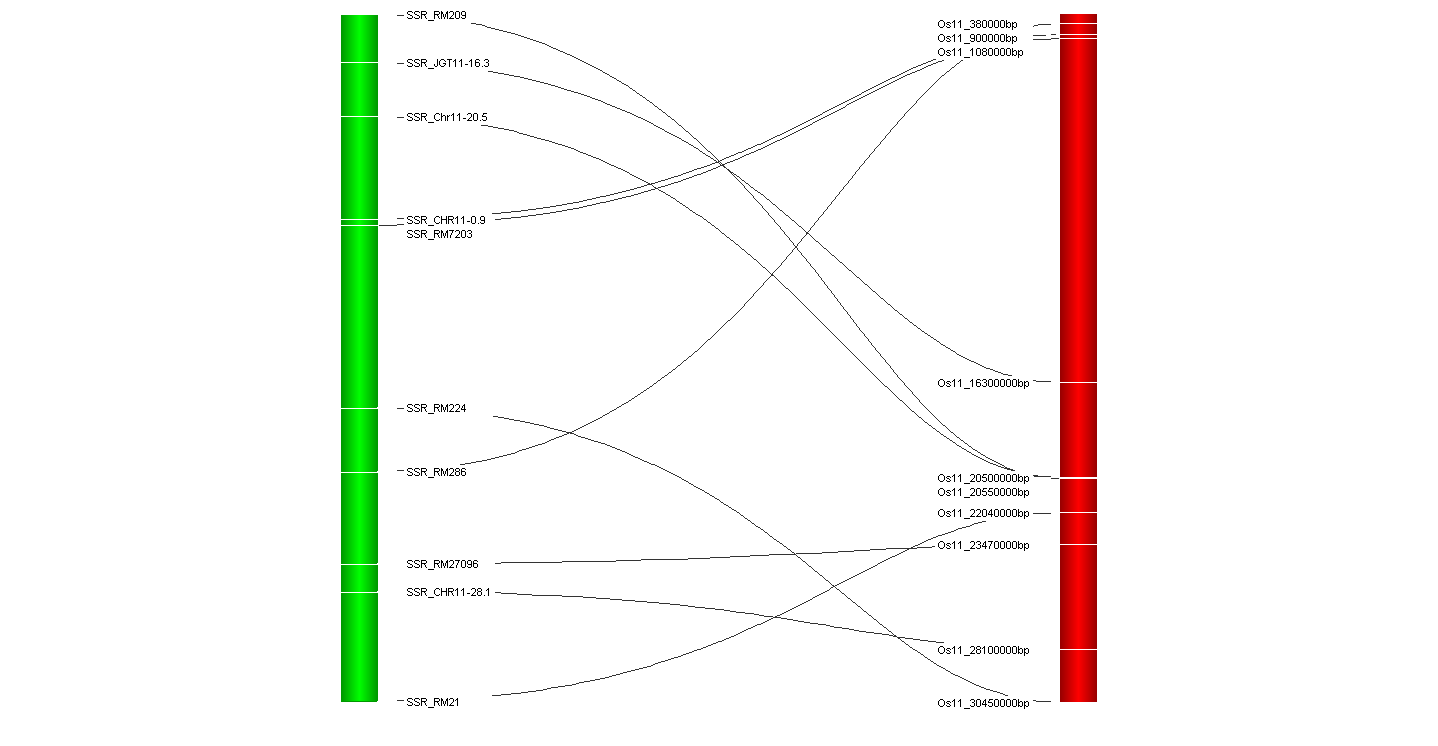


**Genetic map *Os11***

**Physical map *Os11***


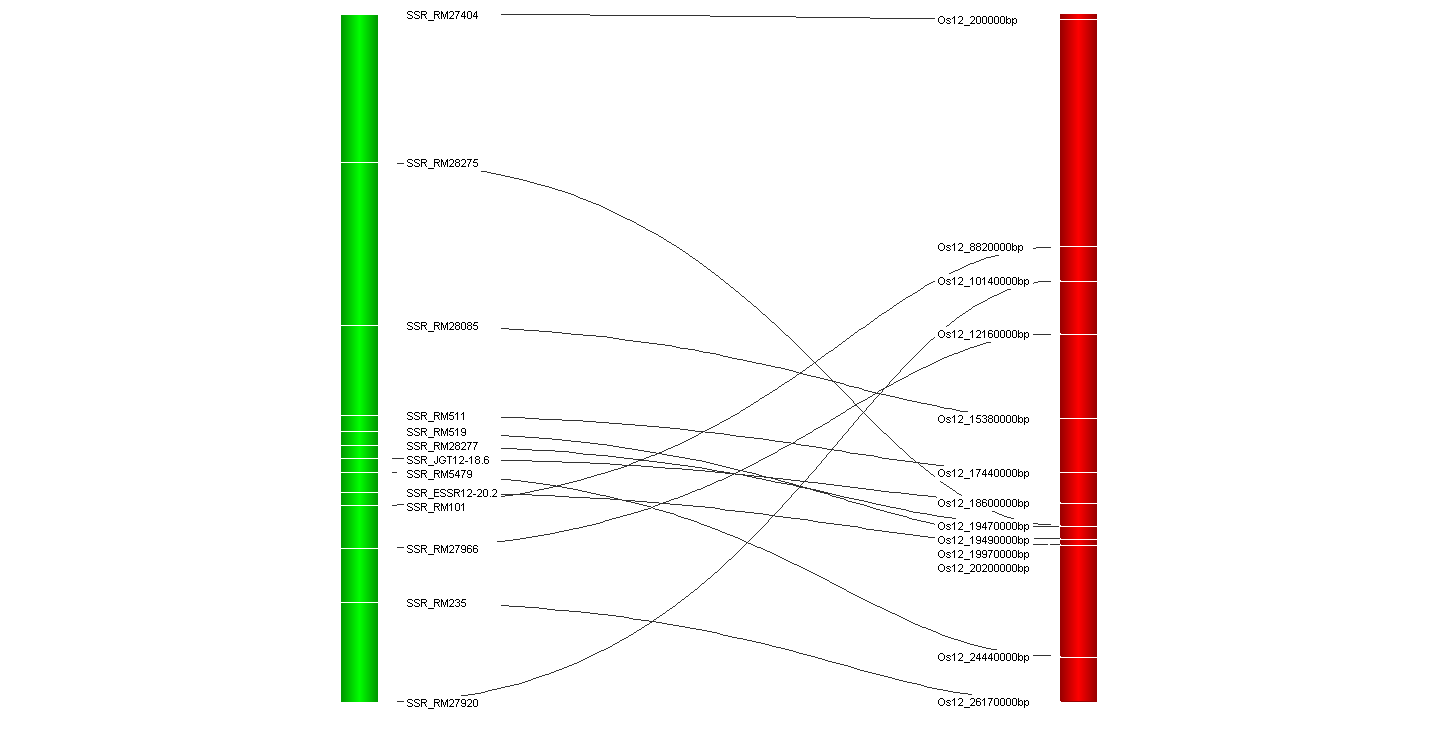


**Genetic map *Os12***

**Physical map *Os12***

Supplementary Figure 3: Collinearity map showing the correlation between the genetic and physical maps for chromosomes 1 to 12; Strudel software, version: 1.15.08.25, https://ics.hutton.ac.uk/strudel/

**(I)**

**(II)**


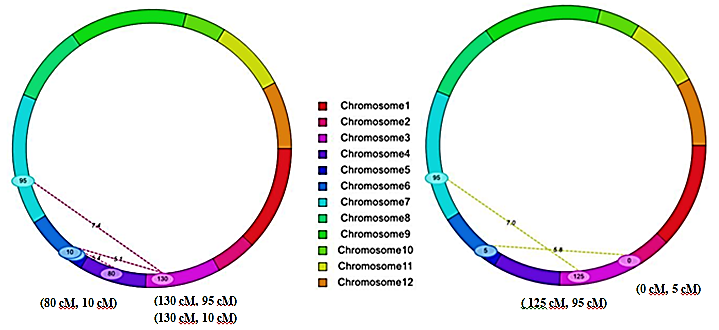


Supplementary Figure 4: In 105 KRH-2 derived RILs-126 SSR marker data set, significant major effect epistatic interactions were observed for the traits grain yield (g) (YLD) and panicle length (cm)(PL). YLD epistatic interaction was noted between chr 3 (130 cM) and chr 5 (10 cM), chr 7 (95 cM). Also, an interaction between chr 4 (80 cM) and chr 5 (10 cM) was noted. For PL trait, epistatic interaction was observed between chr 3(0 cM)-chr 6 (5 cM), chr 3(125 cM)-chr 7 (95 cM). Positions of interactions indicted in brackets.


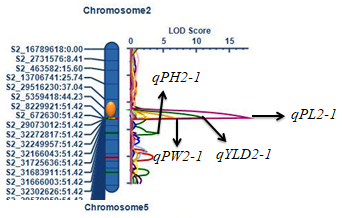

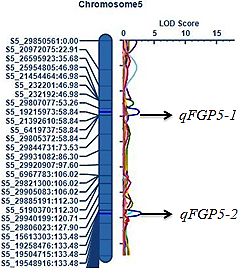

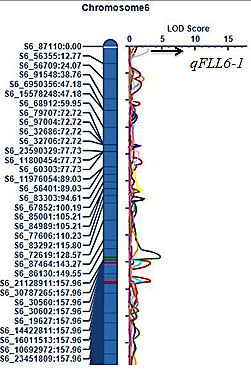

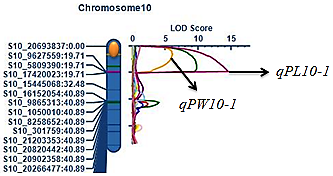

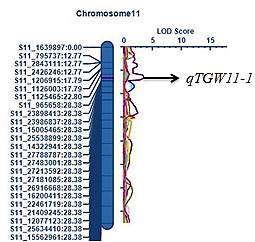


Supplementary Figure 5: Major effect QTLs identified with 24 selected RILs and 1,082 SNPs. QTL hotspot regions indicated with orange bead.


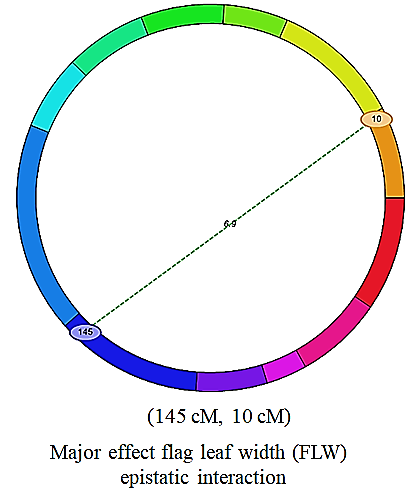

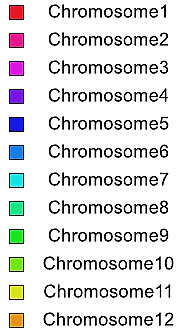

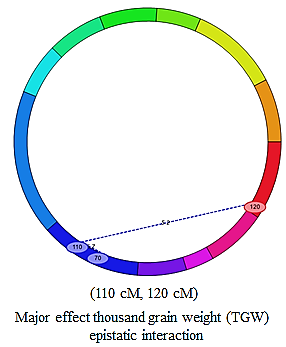


**(II)**

**(I)**

**(III)**


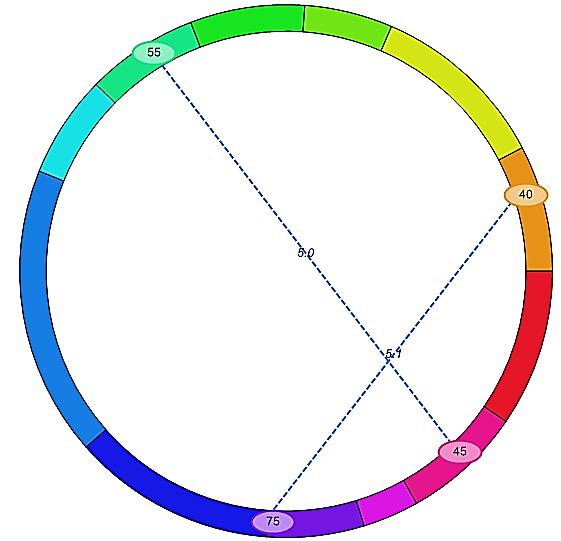

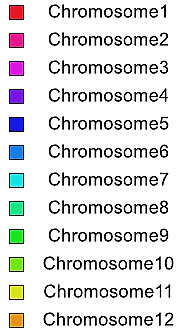

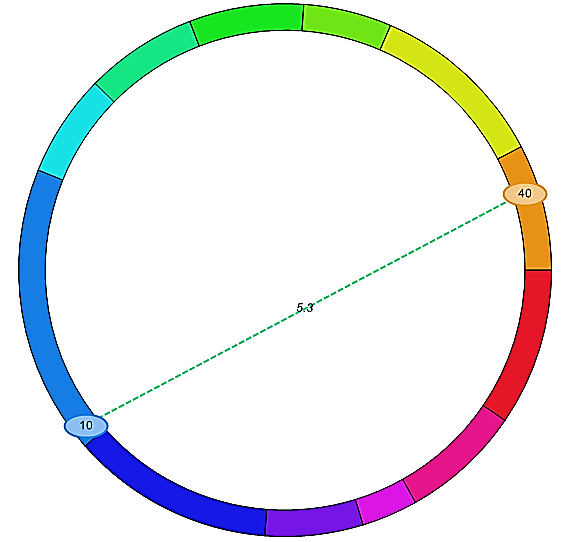


(10 cM, 40 cM)

(75 cM, 40 cM)

(45 cM, 55 cM)

**(IV)**

**(III)**

**(II)**

**(II)**

Major effect flag leaf length (FLL)

epistatic interaction

Major effect panicle weight (PW)

epistatic interaction

Supplementary Figure 6: In 24 selected KRH-2 derived RILs-1,082 SNP marker data set, significant major effect epistatic interactions were observed for the traits (I) thousand grain weight (g) (TGW) and (II) flag leaf width (cm)(FLW). TGW epistatic interaction was noted between chr 1 (120 cM) and chr 5 (110 cM). Also, an interaction between chr 5 (145 cM) and chr 10 (10 cM) was noted for flag leaf width (FLW). Also, significant major effect epistatic interactions were observed for the traits (III) panicle weight (g) (PW) and (IV) flag leaf length (cm)(FLL). PW epistatic interaction was noted between chr 4 (75 cM) and chr 12 (40 cM). Also, an interaction between chr 6 (10 cM) and chr 12 (40 cM) was noted for flag leaf length (FLL). Positions of interactions indicted in cM.
